# The one-humped camel: The animal of future, potential alternative red meat, technological suitability and future perspectives

**DOI:** 10.12688/f1000research.125246.1

**Published:** 2022-09-22

**Authors:** Djamel Djenane, Mohammed Aider

**Affiliations:** 1 Department of Food Science and Technology., University of Mouloud MAMMERI, Tizi-Ouzou, 15000, Algeria; 2 Institute of Nutrition and Functional Foods (INAF), Université Laval, Quebec City, QC, Canada; 3 Department of Soil Sciences and Agri-Food Engineering, Université Laval, Quebec City, QC, Canada

**Keywords:** Camelus dromedarius; future food; health claims; alternative red meat, Comprehensive review, Health food

## Abstract

Camel meat is an ethnic food consumed across the arid regions. For these medicinal and nutritional benefits, it can be a great option for sustainable meat worldwide supply. Consumers can be benefit from the subtle taste of camel meat, flavored with aromatic and medicinal herbs from the arid regions. Research on the camel meat from both an economic and technological aspects is quite recent, which explains the limited information available on this area. Nevertheless, developing new preservation techniques as well the development of various products from camel meat through optimum processing constitute an axis of fu-ture scientific research in order to valorize this product. The camel meat as an alternative source to red meats is also discussed as well as the challenges of its acceptance by consumers. In light of the enthusiasm for this meat, to which many beneficial health effects are attributed, it seemed interesting to conduct this review.

## Introduction

The current enthusiasm for the consumption of camel meat as red meat is based in part on the therapeutic or medicinal virtues that are attributed to it. It is considered lean because it comes from animals fed on natural pastures, and responds to the strong “health claim” trend, which consists of limiting the consumption of red meats and animal fats.

Today, multidisciplinary research is carried out on camel meat for the adoption of efficient production systems, the improvement of its transformation as well as its marketing. In recent years, various researchers have introduced new alternative preservation techniques applicable to all meats to maintain their quality ( Djenane *et al*. 2020). This strategy would extend shelf life while ensuring product safety during distribution and subsequent retail display.

The camel sector is currently facing a gradual opening of borders to animal products. This come from countries where technical and regulatory developments have made it possible to promotion the camel meat industry and improve product quality. The camel sector must adapt to the transition towards a competitive economy to design and implement liberalization reforms in a context marked by agreements concluded between the countries. In arid regions, veterinary control focuses more on animal health compliance for healthy consumption. Despite the efforts made by the veterinary services to ensure safe meat, hygiene conditions remain insufficient ( Yehia *et al*. 2021).

This review discusses the nutritional and qualitative properties of camel meat, explains its versatility and positive effects on human health. The current “health claims” situation of this product and the development prospects through a few technological suitability in progress, and future perspectives are also discussed. In addition, this review brings additional elements which we aim to summarize here. In addition to the knowledge acquired on camel meat, in the view of the most recent works, the current market situation for this product and development prospects will be presented.

## Camel populations (numbers) and distribution

Camel is a polygastric animal, but it is often referred to as “ *pseudo-ruminant*”. Because camels are migratory animals, the number of dromedaries is not known with precision given the drought episodes and the population movements that accompanied it. The world camel population exceeds 35 million heads ( Faye, 2020). *Camelus dromedarius* is the most frequent and widespread domestic camel species composing 90% of the total camel population, whereas the two-humped Bactrian camel ( *Camelus bacterium*) represents the remainder (10%).

Australia plays a special role, as much of the camel herds are kept in the wild. An environmental problem has arisen because of an abundant population that is continuously growing. To solve the problem, the Australian authorities opted for the mass destruction of feral camels. However, another suggested route was the promotion of the meat of this species on local and international markets ( https://www.nintione.com.au).

## Camel meat specifications and consumer perception

For long periods, camel meat was considered a powerful marker of social, ethnic, and religious identities ( Baba *et al*. 2021). Adopted from the beef specifications, the Central Australian Camel Industry Association Inc., presents the camel meat specifications at its website ( http://www.australiancamelindustry.com.au), and some authors have claimed that the adoption of these specifications greatly facilitates the international trade of camel meat ( https://www.nintione.com.au). Many traditional camel meat products characterize Africa and the Middle East regions. However, only a few of them have been reported and characterized scientifically.

SWOT analysis was conducted to identify the camel meat commodity’s strengths, weaknesses, opportunities and threats. The results of this analysis showed that one of the identified weaknesses is the lack of consumer awareness towards camel meat ( Mbaga, 2013). Often, consumers are unaware that camel meat is a healthy product. As a general rule, consumers will not buy any product unless they have heard of it before. Therefore, consumer awareness remains a very important factor. Consumers tend dislike camel meat because they associate meat with the camel itself, and this is often one of the reasons for this disapproval. Under these circumstances, it would be ideal for manufacturers to avoid the use of promotional labels that show the image of the camel itself. The Australian meat industry has been successfully promoted kangaroo meat and lessons can also be drawn from the same approaches ( https://www.awe.gov.au/biosecurity-trade/export/controlled-goods/kangaroo).

Consumer motivations and barriers for buying camel meat were further investigated *via* the survey approach. A previous study is available in the literature regarding small ruminants’ meat consumer preferences ( Brahimi *et al*. 2020). This study was designed to deepen understanding of consumers' perceptions of different meats, as well as the motivations and barriers consumers face when approaching these products. Consumers often mentioned price as an important quality attribute. The health aspect also emerged among participants. Some consumers believed that camel meat had lower fat content compared to other meats, and was particularly suitable for people with diseases.

## Bioactive compounds in camel meat

**Figure 1.**
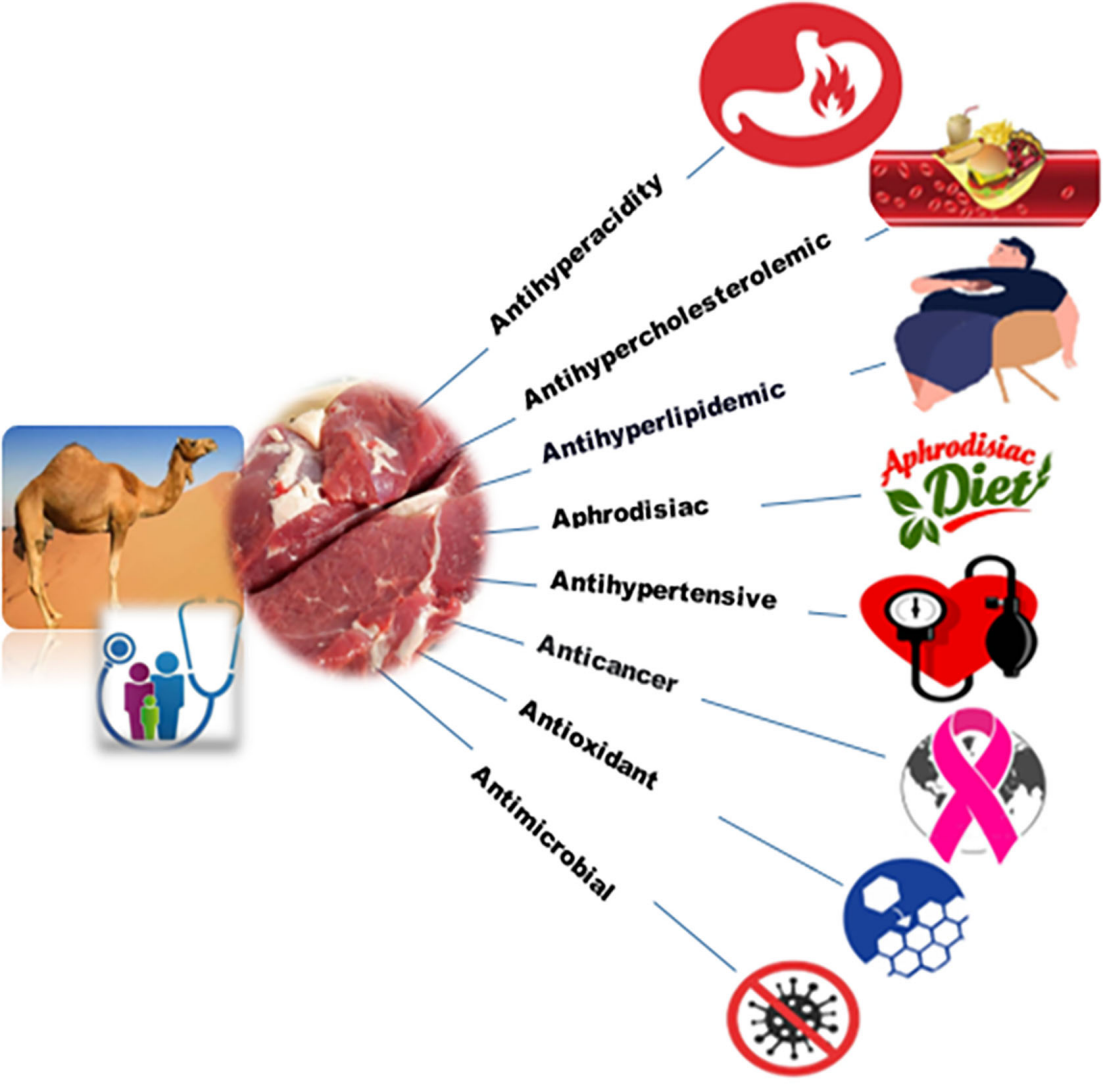
Scheme of the potential medicinal value of camel meat.

Camel meat is characterized by a considerable amount of conjugated linoleic acid (CLA) and monounsaturated fatty acids (MUFAs) ( Popova *et al*. 2021). Scientists found that CLA appeared to reduce tumor growth in several types of cancer and against atherosclerosis ( Kim *et al*. 2016). For this reason, the main objective of new animal feeding strategies is to increase the concentration of polyunsaturated fatty acids (PUFAs) and the levels of CLA in forages. Importantly, the ω6/ω3 ratio in camel meat was lower (≈3) ( Maqsood *et al*. 2015a) than the recommended values of human health diets (≤4.0) by British Department of Health ( BDH 1994). Ayyash *et al*. (2019) claim that camel meat was traditionally used as a remedy for some diseases such as pneumonia, hypertension, hyperacidity, and respiratory disease as well as an aphrodisiac ( Figure 1). Recently, an *in vitro* comparative study, investigated the health-promoting benefits (anticancer) of semi-dry fermented camel sausages fortified by two novel probiotic *Lactiplantibacillus plantarum* compared with fermented beef sausage ( Ayyash *et al*. 2019). Fermented camel sausages showed greater anticancer activity than beef sausages. The same authors found that the antihypertensive *via* inhibition of angiotensin-converting enzyme (ACE), greater cytotoxicity capacities and antioxidant activities in fermented camel sausages were more pronounced than in beef sausages.

Knowledge about camels was traditionally restricted to limited geographical areas, particularly Middle East, Asia, and Africa, but the use of camel’s products as a nutrient for health benefits is currently known worldwide. However, during the 20 ^th^ century, when intercultural migration of people became very important, knowledge about camels and their products began to reach countries beyond Middle East, Asia and Africa. Scientific efforts continue around the world to identify more therapeutic constituents.

Mejri *et al*. (2017a) identified several bioactive molecules in some fermented camel products. The results showed that identified peptides below 3 kDa have an antioxidant and antihypertensive effects. Gheisari and Motamedi (2010) studied the antioxidant enzymes activity in refrigerated camel meat and confirmed its catalase stability. However, glutathione peroxidase (GSH-Px) activity decreased in camel meat during refrigerated storage. L-carnitine (β-hydroxy-γ-trimethyl amino butyric acid) plays a crucial biological role. A study evaluated the concentrations of free carnitine, acylcarnitine and total carnitine from camel meat ( Alhomida *et al*. 1995), and found 5.17, 2.60 and 7.77 μmol/g fresh weight of free carnitine, acylcarnitine and total carnitine, respectively. Similarly, Kadim and Sahi (2018) indicated that camel meat could be a significant source of carnitine. However, a higher proportion of acyl carnitine in plasma and skeletal muscle of the camel than other animal species suggests an adaptive mechanism that could be common to camelids, which may provide energy to various tissues during scarcity of water and feed for long periods.

An important dipeptides such as carnosine (β-alanyl-L-histidine) and its derivative anserine (β-alanyl-L-methyl-L-histidine) are found in high concentration in the muscle of mammalian species. Dromedary camel meat contains 164.9 mg carnosine/100 g and 236.9 mg anserine/100 g fresh weight ( Kadim and Sahi 2018). Carnosine has been proven to act as antioxidant in various meat systems ( Sánchez-Escalante *et al.* 2001; Djenane *et al*. 2004), and also as anticancer actions in various model systems by the restoration of normal cellular homeostasis ( Turner *et al*. 2021). The average levels of carnosine and anserine in camel muscles has 182 and 269 mg/100 g fresh weight, respectively. Little has been known about antioxidant enzymes in camel meat. Chafik *et al*. (2020) suggested a new carbonic anhydrase enzyme that was purified and differentiated from camel liver for atmospheric CO _2_ sequestration. Other endogenous antioxidants enzymes were found in camel muscles such as superoxide dismutase (SOD), coenzyme Q10, catalase and GSH-Px (selenium-containing enzyme) contribute to oxidative defense and stimulates the reduction of harmful free radicals ( Gheisari and Motamedi 2010).

## Organoleptic properties of camel meat

To enhance the image of camel meat, and the search for new outlets for this meat, the camel sector must promote these organoleptic qualities in the same way as other red meats ( Halagarda and Wójciak 2022), but also, strengthen its image with additional nutritional benefits.

The ultimate pH (pHu) of camel muscles (5.5 to 6.6) is a consequence of lactic acid accumulation *via* glycolysis and is considered one of the main factors determining the organoleptic characteristics of meat. Soltanizadeh *et al*. (2008) found that the pH decline was faster in beef meat than camel. A more rapid pH decline may inactivate protease activity, meaning a reduction in the proteolysis and subsequent *post mortem* tenderization. Therefore, there is a possibility that the higher myofibril fragmentation index (MFI) observed in camel meat was due to its higher *post mortem* pH values, and consequently for higher protease activity ( Kadim *et al.* 2006).

### Expressed juice

The importance of moisture in camel meat is in its marked effects on its processing potential, quality characteristics and shelf life optimization during storage. Meat cuts with low water holding capacity (WHC) are drier and would lose more weight during refrigeration, storage, transportation and marketing. Result of this, loss of minerals, salts and vitamins ( Kadim *et al*. 2013). In meat, juiciness and tenderness are closely correlated. The more tender the meat, the faster the juice is released by chewing it. Meat with higher pH value has a greater WHC than that with a low pH value; often referred to as: dark, firm and dry (DFD), and pale, soft, exudative (PSE) meats, respectively ( Liu *et al*. 2021).

### Tenderness

Meat tenderness is often determined by muscle characteristics, collagen content and its solubility, *post mortem* glycogen concentration, proteolytic enzymes content and more likely in the enzyme/inhibitor ratio, a parameter reflecting the efficiency of the proteolytic systems ( Nair *et al*. 2019). Reports that camel meat is less tender than other animals are likely due, at least in part, to the higher average age of slaughtered animals, mainly obtained from old animals (aged >10 years). A number of studies have shown that Warner–Bratzler–Shear Force (WB-SF) values increase with animal age. The values reported in the literature, indicate higher values than those reported for beef.

Kadim *et al*. (2006) suggested that the shear-force value from aged camels (8 years) was 48% higher than that from young camels (1–5 years). The WB-SF values for various camel muscles were determined by Kadim *et al*. (2013). The *infraspinatus*, *triceps brachii*, *longissimus thoracis* muscles have significantly lower shear-force (6.3–6.7 kg) values than *semitendinosus*, *semimembranosus*, and *biceps femoris* muscles (9–12.9 kg), which might be due to less connective tissue. Suliman *et al*. (2019) reported a similar result.

Consumer acceptance of identified individual muscles should be assessed with the aim of developing positive marketing strategies towards consumer perception of camel meat. However, evidence suggests that camel meat tenderness is not significantly different from beef if it is slaughtered at the same ages ( Kadim *et al*. 2011). Many exogenous enzymes have the capacity to degrade muscle proteins, leading to this effect, a significant degradation of the miofibrillary system, and consequently, a better sensory qualities of obtained meat ( Bagheri Kakash *et al*. 2019). The MFI of old camels (6 years) was lower than youngers (<3 years) ( Al-Owaimer *et al*. 2014). The authors observed a strong relationship between physical disruptions of the myofibrils and the tenderness. MFI is one of the most widely used methods to determine *post mortem* proteolysis in meat of various species. This index is a very useful indicator of meat tenderness. Smili *et al*. (2022) reported that MFI of Algerian Sahraoui dromedary meat was significantly affected by both slaughter age and *post mortem* period. The same authors confirmed that the 30-kDa band is a proteolytic product of Troponin-T in camel *longissimus lumborum* muscles. Soltanizadeh *et al*. (2008) observed the appearance, on the third day of storage, of a 30-kDa band in camel meat which was linked to the higher degree of proteolysis. Therefore, various methods have been developed for tenderizing camel meat. Abdel-Naeem and Mohamed (2016) found that addition of *Zingiber officinale* extract in camel meat burgers resulted in higher MFI. The tenderness of meat can also be improved by the use of exogenous enzymes which break down muscle proteins including connective tissue. Nevertheless, the action of proteolytic enzymes on collagen is limited, which makes them less useful for old camels’ muscles with high collagen content. Recently, plant proteases have been used as a sustainable manner to improve the texture of tough camel meat and to develop functional meat products that contain bioactive molecules with antioxidant activities ( Gagaoua *et al*. 2021). From another perspective and to overcome the problem of toughness in camel meat, Smith *et al*. (2016) showed that whatever the age of the animal or the sex, electrical stimulation (ES) could drastically decrease the hardness of camelid meat. ES of carcasses at 20 to 100 V is a proven method to prevent fibers cold shortening and thus to improve tenderness of meat ( Polidori and Vincenzetti, 2017). The effects of *post mortem* ES (90 V)/20 min was assessed by Kadim *et al*. (2009), authors found that muscles from young camel were not affected by cold shortening and ES had a significant effect on meat quality attributes. Muscles from electrically non-stimulated carcasses have a higher WB-SF value (9.47 kg/cm ^2^) compared to those of stimulated carcasses (6.97 kg/cm ^2^) ( Kadim *et al*. 2009).

Carcass suspension methods can also have a direct effect on meat tenderness. Indeed, the “tenderstretching” mode favored the tenderness of camel meat ( Smith *et al*. 2016). The combination of ES/tenderstretching further promotes tenderness of camel meat ( Biffin *et al*. 2020). The key mechanism by which ES improves tenderness is by rapidly decreasing the concentration of adenosine triphosphate (ATP), which reduces the potential for myofibrils to contract and cold shortening if *post mortem* carcasses are immediately refrigerated.

### Color

Camel meat is described as “raspberry red” and sometimes brown in older animals (due to a higher concentration of myoglobin: Mb) ( Kadim *et al*. 2013). Abdelhadi *et al*. (2013) reported that there are differences in the color of camel meat with a seasonal effect linked to the diversity of animal feeding throughout the year. It was significantly darker red in autumn compared with summer and less yellow in autumn than in summer and winter. In addition to season’s differences, storage temperatures were reported to affect muscle biochemical characteristics and color. The color characteristics of camel meat can be influenced by the *post mortem* storage period and the type of muscle ( Suliman *et al*. 2019). A high redness (a*) color component was associated with a lower lightness (L*), which might be due to an increase in Mb content ( Maqsood *et al*. (2015a). However, meat from older camels have lower L* (darker) and higher a* (redder) than that of younger camels ( Kadim *et al*. 2006). *Post mortem* proteolysis increases light scattering properties of meat and thereby affects L*, a* and b* (yellowness) values ( Hughes and Anderson 2020), which is also directly related to the pH _u_. Meat proteins have an isoelectric pH (pH _i_) of 5.5. This results in open muscle structure and as a result lighter scattering between the myofibrils, which makes the surface of the meat lighter. It is obvious that during the retail display, the meat surface color is unstable. This phenomenon is due to several factors such as, *post mortem* biochemical changes in protein and lipid fractions of the meat, surface microbial overgrowth, oxidation of lipids and Mb and finally, the phenomenon of photo oxidation due to the fluorescent tube installed in the refrigerated display cases ( Djenane *et al*. 2001, 2003). In the USA, an average of 2.55% of total red meats sold are discarded annually due to discoloration (13.4 million kg, the equivalent of USD 3.73 billion loss) ( Ramanathan *et al*. 2022).

Strategies for preserving color of camel meat were the application of *post mortem* low voltage ES. Abhijith *et al*. (2020) reported that meat from electrically stimulated carcasses, have a brighter red color than meat from -non-stimulated carcasses. Using appropriate packaging and cold storage conditions (vacuum, modified atmosphere packaging (MAP) and active packaging) can play a major role in color enhancement of meat during storage ( Nerin *et al.* 2006; Camo *et al.* 2011; Lammi *et al.* 2018; Ait Ouahioune *et al.* 2022a). Maqsood *et al*. (2015b) reported that camel meat total haem content expressed as haematin was 92.3 mg/g which is higher than what was found in beef (76.16 mg/g). However, the presence of a high haem content could contribute to fast meat pigment oxidation during its refrigerated storage. Average Mb contents in camel meat was found to be 7.16 mg/g ( Maqsood *et al*. 2015a). In contrast, Gheisari (2011) reported 3.64 mg/g. This difference could be due to several experimental factors such as part of the carcass, solvents and extraction conditions. In general, the quality of camel meat has been assessed using traditional conventional methods, and it will be important for the camel sector to take into account the new technologies already applied to beef.

## Technological aspects of camel meat

### Processing technologies

Camel meat has been processed into burgers, cured or cooked ham, kebabs, meatballs, patties, mortadella, merguez, sausages, and shawarma to add value ( http://www.australiancamelindustry.com.au). Australian processed camel meat has been accepted as international traded meat products (Wu *et al*. 2011). It is now exported to Canada, China, Europe, Saudi Arabia and the USA. The potential of camel meat has received increased attention in recent times. In this context, Kargozari *et al*. (2014) compared the fatty-acid and volatile-compound profiles, sensory qualities and the physicochemical of dry sausages (Sucuk) made from camel meat and hump fat with sausages made from beef and beef fat and from a mixture of both. The authors suggest that the physicochemical properties and some textural attributes of camel meat sausages were comparable to those of beef. Nerveless, the flavor components were quantitatively much higher in camel sausages. The same authors concluded that camel meat can successfully replace beef in sausage manufacture.

The growing concern of consumers regarding the food health and safety issues has led to the development of products that promote health and well-being beyond its nutritional effect. Fermented meat products can be considered as relevant matrix for the delivery of bioactive molecules with potential health benefits ( Mejri *et al*. 2017b; Ayyash *et al*. 2019). In a comparative study, Kargozari *et al*. (2014) found that fermented camel sausages exhibited greater resistance to lipid oxidation (lower thiobarbituric acid reactive substances: TBA-RS) during storage compared to beef sausages. These authors obtained an ω-6/ω-3 ratio of 6.22 for beef sausages and 2.95 for camel sausages. This indicates that camel sausages fit perfectly into the recommendations for this ratio ( BDH, 1994). Abdel-Naeem and Mohamed (2016) found that addition of ginger ( *Zingiber officinale*) and papain ( *Carica papaya*) extracts in camel burger resulted in improvement of the lipid stability and significant increase of sensory scores of treated burgers during storage. Gagaoua *et al*. (2021) showed that marinade with plant proteases has been used to improve the texture of tough camel meat. This can be a valuable strategy to develop locally tender camel and healthier products. Maqsood *et al*. (2015b) studied the effect of phenolic compounds such as tannic acid and catechin on sensory quality and fatty acid profile of camel meat during storage, and found that incorporation of these compounds improved sensory scores and fatty acid profile. Furthermore, authors suggest that tannic acid and catechin could prevent protein breakdown in chilled ground camel meat, which was most likely due to their antimicrobial and antioxidant activity. Despite the low cholesterol content of camel meat, the salt curing process can induce significant amounts of cholesterol in the final products (Mamani-Linares *et al*. 2014). To avoid this problem, solar drying under the open sky of camel meat without salting was carried out by Chaouch *et al*. (2018).

The growing demand for Halal gelatin, and the rejection of gelatin from a mainly porcine source have encouraged scientists to search for alternative sources such as camel skin ( Asif Ahmed *et al*. 2020). Recently, Al-Hassan (2020) demonstrated that gelatin could be extracted from camel skin as a promising source of Halal gelatin.

### Packaging technologies

The evolution of modern retail outlets with better packaging, labeling, and cold chain facilities will address the drawbacks of the current situation in the camel meat sector. Referring to packaging technologies, very little data are available on the application of innovative techniques in camel sector ( Table 1). Microbial spoilage associated with color change and lipid oxidation are the critical factors limiting shelf-life and consumer acceptability of stored camel meat ( Maqsood *et al*. 2015b). Haem pigment is known to be potent catalyst of lipid oxidation in muscle foods. With increasing storage time, damage to haem pigment results in the release of ferrous (Fe ^+2^), which can accelerate lipid oxidation ( Gheisari, 2011). Its role as catalyst of lipid oxidation is because is capable of catalyzing the production of reactive oxygen species (ROS) such as superoxide anion (O _2_^•−^). This process is frequently explained by the Fenton reaction. Alkyl radicals can be formed as a result of the reaction of O _2_^•−^ with fatty acids and hence the onset of lipid oxidation. Moreover, O _2_^•−^ could be transformed into hydrogen peroxide (H _2_O _2_) by dismutation, which react with ferrous iron (Fe ^+2^) to produce the hydroxyl radical (OH•).

**Table 1.** The reports of the last decade (2009-2022) on strategies for enhancing the safety, quality and shelf-life of camel meat.

References	Product and conditions	Results/Conclusions
Osaili *et al*. (2020b)	Fresh ground camel meat	The use of abusive temperatures to preserve meats is common in arid countries. A study on the growth of pathogenic and spoilage bacteria in fresh ground camel meat was conducted during storage at 4°C and 10°C for one week. Meat samples were inoculated with *Escherichia coli* O157: H7 and *Salmonella* spp. and treated in a water bath at 55–65°C. At 4°C, the number of *E. coli* and *Salmonella* spp. decreased drastically in the samples. In contrast, samples stored at 10°C showed significant increases in microbial populations. Regarding the spoilage flora, a significant increase was observed in both cases of storage temperatures. These results can be a tool to help improve the safety and quality of camel meat during storage, and to facilitate the validation of the industrial heat treatment of camel meat products.
Osaili *et al*. (2020a)	Camel meat burgers	Adding camel's hump fat to meat products could result in increased thermal resistance of bacteria in the matrix. The results of this study could help camel meat processors to validate thermal processes for public health purposes.
Osaili *et al*. (2021a)	Marinated camel meat chunks	The antimicrobial effect of a yogurt-based marinade associated with carvacrol, thymol or cinnamaldehyde in camel meat stored at 4 and 10°C for 7 days was studied. At 10°C, the synergistic effect of this association was greater compared to 4°C. This technique may have a complementary effect to the lethal effects of heating, and therefore improve the safety of camel meat against *E. coli* O157: H7 and *Salmonella* spp., without significant changes in the sensory characteristics of cooked camel meat. These results can be used as an effective tool to promote the safety of fresh camel meat, specially in arid rgions.
Osaili *et al*. (2021b)	Packaged Marinated Camel meat	The use of the combined method of vacuum packaging (VP) with EOs and their components (carvacrol, cinnamaldehyde, and thymol) could increase microbial stability of marinated camel meat during storage even at abuse temperature (10°C). The Incorporation of 2% EO into marinated camel meat stored at abusive temperature (10°C) under aerobic conditions improved product shelf-life.
Djenane *et al.* (2020)	Packaged fresh camel steaks	The highly modified oxygen atmosphere (80% O _2_) combined with leaf extract *Olea europaea* Subsp. laperrinei treatment alone, or combined with nisin, can be a promising tool and constitute a relevant strategy to enhancing the shelf-life of packaged camel meat at 1 ± 1°C by control microbial growth and oxidation phenomena without undesirable effects on its sensory acceptability during 30 days of storage.
Jouki and Khazaei (2012)	Vacuum packaged camel meat	Vacuum packaged camel meat can be a promising tool and constitute a relevant strategy to enhancing the shelf-life of packaged camel meat by control lipid oxidation phenomena. Lowest degrees of lipid oxidation value was obtained for vacuum packaging compared to modified atmosphere packaging (60% CO _2_; 40% N _2_) and air packaged during chilled storage.
Khezrian and Shahbazi (2018)	Packaged minced camel meat	To increase the shelf-life (microbial, chemical and sensory properties) and against *Listeria monocytogenes* and *Escherichia coli* O157: H7 growth during chilled storage a novel films based on nanocomposite incorporated with *Ziziphora clinopodioides* EO alone and in combination with *Ficus carica* extract were investigated as active packaging materials for minced camel's meat. Minced camel meat's shelf-life can be extended by using chitosan film containing natural biomolecules separately and in combination with cellulose nanoparticles. The synthesized designated films are biodegradable, thus potential in modern active food packaging for the concern of food protection and environmental problems.
Maqsood *et al.* (2015b)	Fresh camel meat	Upon addition of different natural phenolic compounds at a level of 200 ppm, thiobarbituric acid reactive substances (TBA-RS) as well microbial counts were retarded, especially in samples added with tannic acid and catechin compared to control samples without sensory modification of camel meat on day 6 of storage.
Al-Bachir and Zeinou (2009)	Irradiated Fresh camel meat	Treatment of camel meat with gamma irradiation (2, 4 and 6 kGy) reduced the total bacterial flora in treated meat. Thus, the microbiological shelf-life of camel meat was significantly extended from less than 2 weeks (control) to more than 6 weeks (treated samples). No significant differences in thiobarbituric acid (TBA) values, volatile basic nitrogen (VBN) and sensory properties of camel meat were observed due to irradiation.
Jouki and Khazaei (2012)	MAP and fresh camel meat	By using an anoxic modified atmosphere packaging with higher carbon dioxide (60% CO _2_ + 40% N _2_) and combined with refrigeration (4°C) can be used as an effective tool to promote the physico-chemical attributes of fresh camel meat in arid countries without any noticeable sensory changes during three weeks of storage.
Yehia *et al.* (2021)	Vacuum and Fresh camel meat	The natural biopreservatives (Citrox + chitosan) constitutes a promising tool and a relevant strategy for the control of *Campylobacter jejuni* and also for preserving the quality of camel meat packed under vacuum at 4 and 10°C for 1 month.
Ansarian *et al. *(2022)	Raw minced camel meat	*Mentha spicata* EO was incorporated at different concentrations (0.5 to 1.5% v/w) into raw minced camel meat. The final microbial population decreased 1 to 4 log cfu/g in treated samples compared to control. *M. spicata* EO as a natural substance could successfully extend the shelf-life and control of *Listeria monocytogenes* in fresh minced camel meat stored at refrigerated temperature for 12 days. Color, odor and also appearance were better in treated samples than in control samples. *M. spicata* EO can be used as a replacement to synthetic preservatives as well as synthetic flavorings in minced camel meat.
Hai *et al*. (2020)	Nanoemulsion-based film and minced camel	Minced camel meat was packaged in nanoemulsion-based film with EO during 3 weeks’ storage at 4°C. Furthermore, meat wrapped with nanoemulsion-based film containing EOs showed better oxidative stability and shelf-life results compared with the control group.
Zhao *et al*. (2020); Husain *et al*. (2021)	Authentication of camel meat	Authentication of meat products is important to ensure fair competition, consumer benefit, and food safety. The difference in price between camel and other species may be an incentive to adulterate meat products. With an increased demand for camel meat, camel meat-related food products are susceptible to food fraud. These food frauds can be detected in a very specific way and relatively quantified thanks to triplex real-time polymerase chain reaction PCR assay or real-time PCR-lateral flow immunoassay (LFI) (making it possible for example to determine the percentage of each animal species in relation to the total amount of meat). Overall, this new method could be ideal for government laboratories to detect food fraud of this kind. The designed triplex real-time PCR assay was shown to be a specific, sensitive, and efficient technique for the identification of camel and other species DNA in foodstuffs.
Khan *et al*. (2021)	Biofilm formation and camel meat	Biofilm formation by drug-resistant pathogens poses a major threat to food safety and public health. Camel meat can harbor biofilms formed by the genus *Pseudomonas* spp. producing metallo-β-lactamase (MβL) enzymes. The effect of the flavone naringin on biofilm formation produced by *Pseudomonas* spp. has been determined. Naringin significantly reduced biofilm formation (57% of reduction). Thus, it is envisaged that naringin can be used as a natural food preservative against the formation of biofilms in the meat industry.
Chaouch *et al.* (2018)	Dehydrated camel meat without salting	Experiments were carried out for drying camel meat without salting under desert conditions. Controlling camel meat drying conditions aims to maintain nutritional quality, especially protein, which is very sensitive to temperature and salt. This provides the consumer with a guarantee of a dry product that meets hygiene standards and high nutritional quality. The development of solar drying technology and capacity of solar energy storage technology is made possible in the Saharan regions. This ancestral technology will allow the creation of a camel meat drying industry and promote camel breeding by installing this new mode of consumption, neglected for a long time.
Eom *et al*. (2015)	Tough camel meat and tenderizing agents	The action of proteolytic enzymes on collagen is limited, which makes it less useful for muscles from old camels with high collagen content. Plant proteases have been used as a sustainable manner to improve the texture of tough camel meat and to develop functional meat products that contain bioactive molecules with antioxidant activities. Attempts to include tenderizing agents such as ginger extract and papain and their mixture in the formulation of camel meat patties to improve the physico-chemical and sensory characteristics of the product have been carried out. Treatment with tenderizing agents resulted in a significant reduction in shear-force values and an increase in collagen solubility.
Maqsood *et al*. (2016)	Vacuum packaged camel meat	The Impact of different packaging conditions on various quality attributes of camel meat during 18 days of refrigerated storage was investigated. Camel meat packed under vacuum displayed lower thiobarbituric acid reactive substances (TBA-RS) associated with *off-odor*, lower counts for different microorganisms, higher Redness (a*) values and major scores on odor, color and overall acceptability compared to controls (air and wrapped). Therefore, vacuum packaging was very effective in retarding lipid oxidation, microbial growth and protein degradation, as well as maintaining the sensory quality for the fresh camel meat.

Djenane *et al*. (2020) reported that high oxygen (80%) modified atmospheres associated with olive leaf extracts ( *Olea europaea* Subsp. laperrinei) and nisin, a polycyclic antibacterial peptide produced by *Lactococcus lactis* can be used as a strategy to retain the redness of camel meat during retail display. Jouki and Khazaei (2012) found the lowest lipid oxidation degrees for vacuum packaged camel meat compared to anoxic MAP (60% CO _2_; 40% N _2_) and air packaged meat during chilled storage, and concluded that vacuum packaging can be used as an effective strategy against deleterious consequences of lipid oxidation. In this same perspective, Maqsood *et al*. (2016) reported that the shelf life of chilled vacuum stored camel meat could be extended to 18 days compared to wrapped samples.

Modern food packaging provides a way to make food safe, reliable, shelf-stable and clean. However, the packaging, without departing from its irreplaceable role in food preservation, is accused today of polluting food and polluting the environment ( Ncube *et al*. 2020). Unfortunately, most food packaging is designed to be single use and is not recycled. The world's population continues to grow and so do natural resources demand. Consumers not only care more and more about what they eat, they are also more concerned with the packaging of the food they buy. This increased focus on environmentally friendly consumption leads to the development of more sustainable business practices and a low-cost circular economy ( Baghi *et al*. 2022). Because of the inability of plastics to degrade, the environmental pollution it causes is a major global concern ( Chamas *et al*. 2020). To overcome this serious problem of plastic waste, the modern food industry requires new and effective approaches to preserving perishable food products. The ideal packaging should have a lower carbon and water footprints, be biodegradable and/or compostable, uses waste or by-products, is eco-designed and safe, and has the right preservation properties to minimize food waste ( Moshood *et al*. 2022; Ait Ouahioune *et al.* 2022b). The incorporation of bioactive molecules (antimicrobial and antioxidant) in packaging materials can remarkably help extend the shelf-life of perishable foods by retarding microbial growth and reducing oxidation ( Djenane *et al*. 2016). Nanotechnology can also play a fundamental role in the meat industry, from processing, preservation to packaging of fresh or processed meat products ( Lamri *et al*. 2021).

### Preservation and extended shelf life

**Figure 2.**
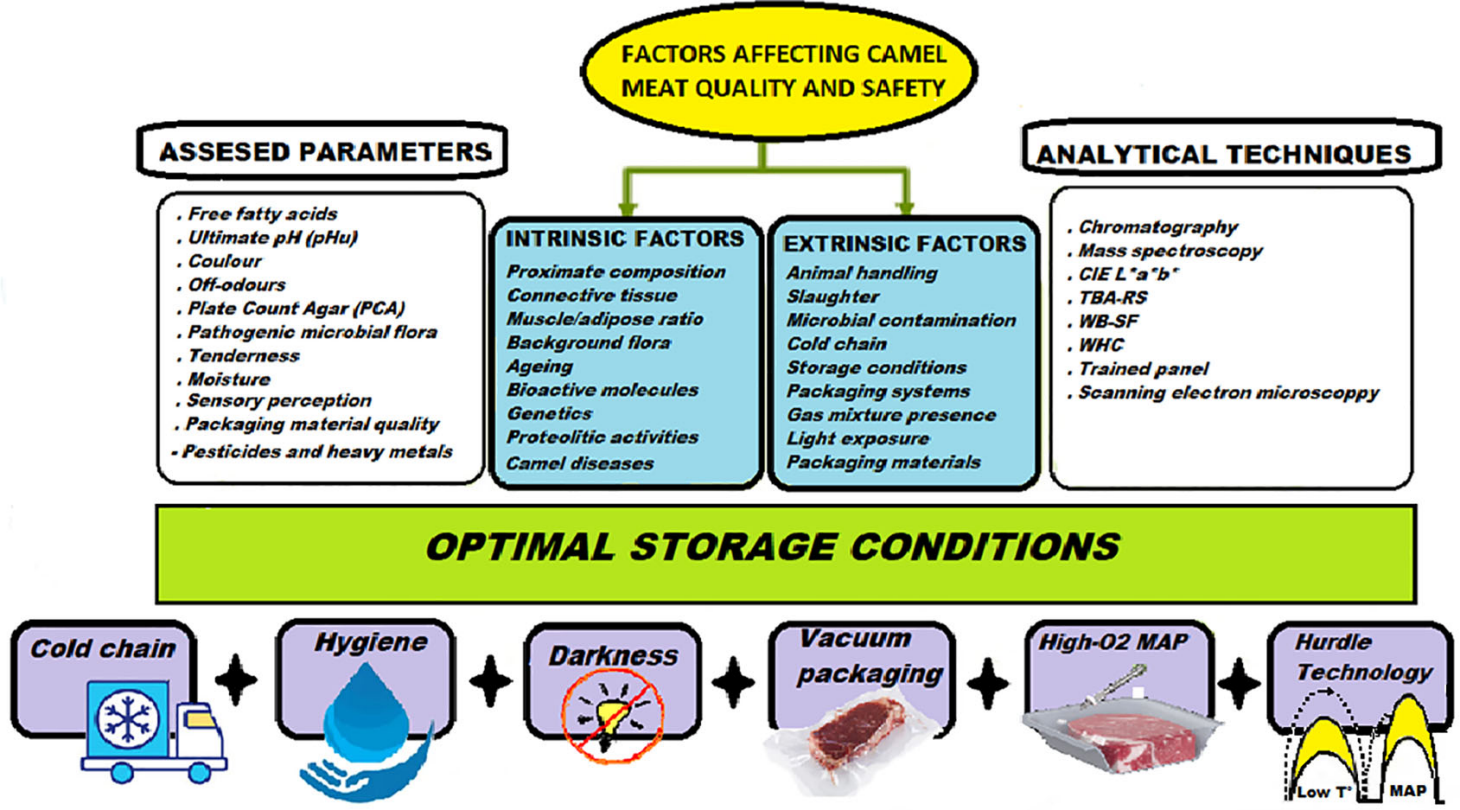
Factors affecting camel meat quality and safety and its preservation.

Over the past decade, meat products commercialization strategies have notably changed all over the world ( Juárez *et al*. 2021). However, as a result of increasing demand for fresh and ready-to-use meats, a need has emerged for adequate preservation techniques to maintain their quality and safety. These technologies include chilled storage, vacuum and MAP, biopreservation, active and biodegradable packaging, and their combination ( Figure 2).

Camel meat is rich in Mb (haem protein) and several authors have reported the pro-oxidant effect of this protein in its oxidized state on lipid oxidation ( Maqsood *et al*. 2015a,b). This makes camel meat more susceptible to oxidation of lipids and therefore to the development of unpleasant odors ( *off odor*) and can limit its shelf life during storage. To overcome this problem, the use of antioxidants is one of the main strategies to extend the shelf life of camel meat ( Djenane *et al.* 2020). Nowadays, frozen storage of meat is broadly utilized to extend its shelf life over one year ( Dang *et al*. 2021). Additionally, most previous studies do not consider the impact of freezing and cooking on varying nutrient amounts in camel meat. The most important phenomenon observed, when cooking meats, is the thermal denaturation of muscle proteins ( Sayd *et al*. 2016), that may cause loss of juice displaying a direct impact on the nutrient content of cooked meats.

Recently, novel strategies are directed towards the use of natural bio preservatives ingredients, which can minimize lipid and pigment oxidation, and *off odor* development with increase of the color stability and consequently the acceptability of camel meat. Therefore, the use of natural plant-based antioxidants, especially phenolic compounds, could be an effective way to extend the shelf life of stored camel meat ( Djenane *et al*. 2020). Thus, plant extracts not only possess antioxidant activity, but also antimicrobial activity against spoilage bacteria and harmful pathogens ( Djenane *et al.* 2012a, b).

It has been reported that camel meat treated with tannic acid and catechin had lowest TBA-RS value, indicating strong inhibitory effect on lipid oxidation during nine days of storage ( Maqsood *et al*. 2015b). Gheisari and Motamedi (2010) found that the presence of high activities of endogenous antioxidant enzymes such as catalase and GSH-Px could lead to a significant decrease in lipid oxidation during camel meat storage. The PUFAs content in camel meat is still desirable for human consumption; unfortunately, these FAs are likely to be susceptible to rapid oxidation. It has been shown that a level of lipid oxidation in the range of 1.5 to 2 mg/kg of malondialdehyde in camel meat caused its rejection by a trained panel for the unpleasant off odors ( Djenane *et al*. 2020). Studies have shown that in some camelids, the level of lipid oxidation remains low ( Smith *et al*. 2019). This is due to the probably high levels in fat soluble vitamins such as vitamin E and other antioxidants reported in camel meat, which is the result of its supply of aromatic and medicinal herbs from arid regions. Maqsood *et al*. (2015b) investigated the effect of phenolic compounds on microbial quality of camel meat during refrigerated storage. The authors suggested that phenolic compounds were effective in inhibiting microbial growth in meat by chelating specific metal ions essential for microbial growth. In previous study, Djenane *et al*. (2020) found that higher oxygenated MAP (80% O _2_) associated with *Olea europaea* extracts treatment combined with nisin can be a promising tool and constitute a relevant strategy to control microbial growth and oxidation phenomena in camel meat. Similarly, Jouki and Khazaei (2012), using an anoxic MAP (60% CO _2_ + 40% N _2_) combined with refrigeration storage (4°C) have obtained significant improvement in physicochemical and sensory properties of camel meat within three weeks of storage. Recently, Yehia *et al*. (2021) reported that the use of combined natural biopreservative agents (Citrox and chitosan) can be a promising tool to control *Campylobacter jejuni* growth and to preserve packaged camel meat even at abuse refrigeration (10°C) storage. A combination of essential oils (EOs) components like carvacrol, thymol or cinnamaldehyde with the yogurt-based marinade exerts a lethal effect against *Escherichia coli* O157: H7 and *Salmonella* spp. without significant changes in the sensory characteristics of the cooked camel ( Osaili *et al*. 2020b). *Mentha spicata* EO was incorporated at different concentrations (0.5 to 1.5% v/w) into camel meat to evaluate its antibacterial activity at refrigerated storage. The final microbial population decreased until 4 log cfu/g. Moreover, during storage, peroxide value (PV), total volatile basic nitrogen (TVBN) remained lower in treated compared to control samples. Similarly, Khezrian and Shahbazi (2018) incorporated of *Ziziphora clinopodioides* EO in combination with *Ficus carica* extract into active packaging films, and found that packed meats exhibited better safety against *Listeria monocytogenes* and *E. coli* O157: H7. Sensory attributes were significantly enhanced in treated camel meat samples.

Camel meat remains an essential source of protein for arid region populations. Nonetheless, various traditional meat products have long been known in the region and prepared for family or religious feasts. For example, during the “ *Al Adha* feast”, Muslim families ought to slaughter a whole camel to be shared equally between seven families. Surplus meat was then transformed into more stable products. This was achieved by combined treatments in an empirical application. However, the sanitary and hygienic importance, and the perishable nature of these products prompted public authorities to establish controlled slaughter structures. Strict compliance with good hygiene practices in slaughterhouses is therefore essential to prevent bacterial contamination of carcasses, with the aim of maintaining optimal meat quality, thus protecting consumer health.

## Camel meat as a substitute for other red meats

**Figure 3.**
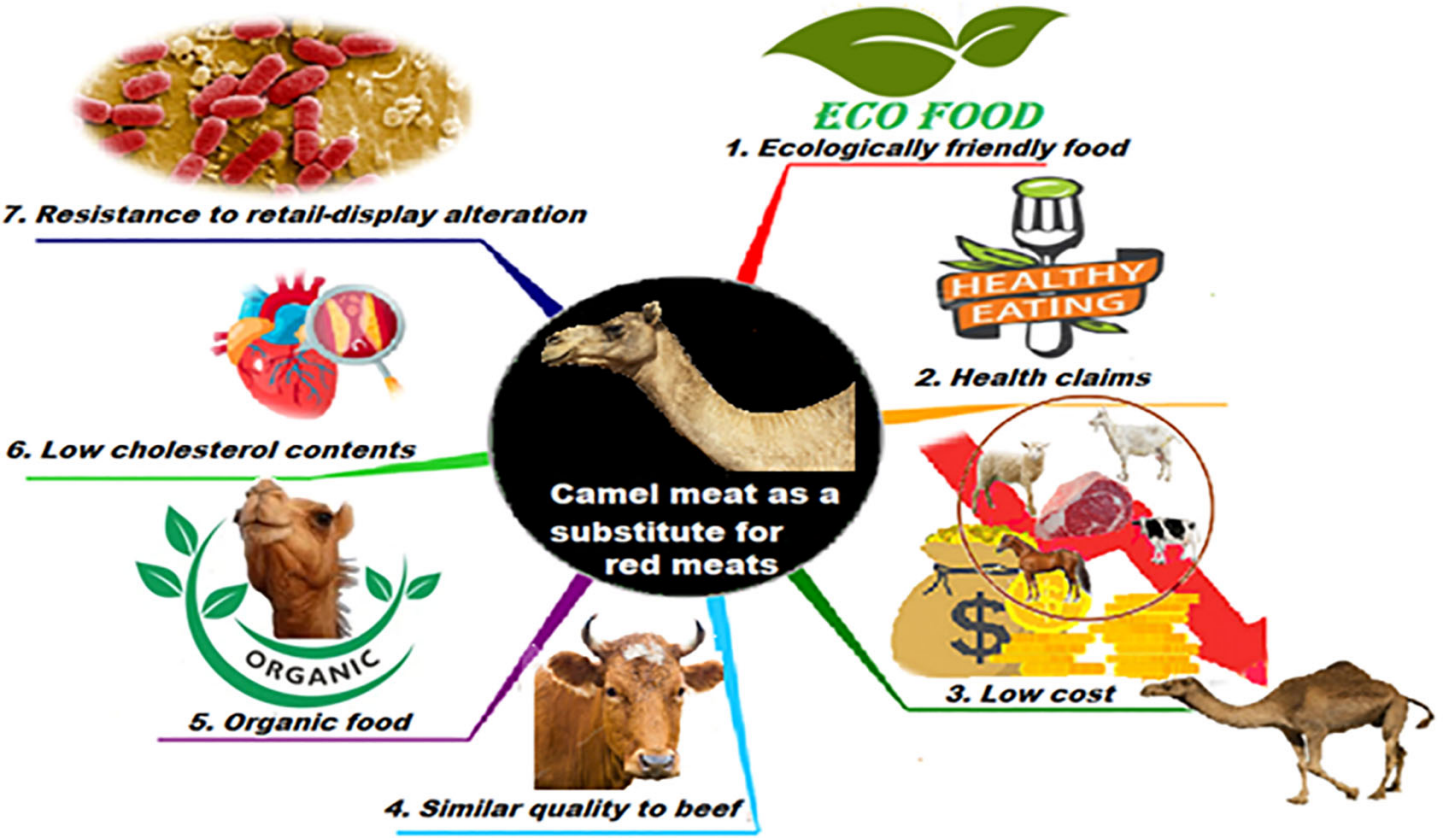
Camel meat as a substitute for red meats.

One of the major challenges for global food security is the high demand for meat products. For these medicinal and nutritional benefits, camel meat can be a great option for sustainable meat supply ( Baba *et al*. 2021). In addition, the price of camel meat is often lower than that of cattle and sheep, due to lower transaction costs and circuits with fewer intermediaries, for a production that also remains very extensive and therefore with few inputs. This allows access to meat proteins for the often most disadvantaged populations and ensures that camel meat has a certain competitiveness. Hence, the one-humped camel as a meat source seems to present a viable alternative to cattle ( Figure 3). The general qualitative characteristics of camel meat are very similar to those of beef. Moreover, it is difficult for an uninformed consumer to tell the difference ( Suliman *et al*. 2020).

During the last decades, the world consumption of camel meat is increasing and the main “camel meat eaters” with more than 3 kg/habitant/year are in Emirates, Mauritania, Mongolia, Oman, Somalia and Western Sahara ( Faye and Bonnet, 2012). The cholesterol content of camel meat (50 mg/100 g) is lower than that of other farm animals ( Raiymbek *et al*. 2015). It appears that camel meat can be used as a substitute for beef due to its reduced cholesterol content and is a strong commercial argument for emphasizing the healthy character of this product.

### Ecological harmlessness of camel meat production

The growing demand for meat cannot be met by conventional meat production alone, because 80% of all arable land is already used directly or indirectly for livestock production ( https://ourworldindata.org/environmental-impacts-of-food), and this is unsustainable as it is, due to its large ecological footprint. By 2050, the world's population will reach around 10 billion people, according to a new report published by the United Nations Department of Economic and Social Affairs ( https://population.un.org). A great challenge awaits policy makers and ensuring food security without compromising the main pillars of sustainability is one of the main objectives of the United Nations for sustainable development. In this optical, the camel sector must adopt sustainable practices in order to become more competitive ( Faye, 2013). Compared to other species such as goats, cattle and sheep, camel is less destructive for the fragile pasturelands. Camel meat is therefore an ecologically friendly food. Camels also have a very efficient feed conversion rate. Nowdays consumers, especially in rich civilizations, tend to favor produces that are environmentally friendly; as a result, this is a very important attribute that needs to be promoted in favor of camel meat.

In arid regions, camel herds are often dispersed over large areas rather than clustered like cattle and sheep. However, camels eat only small amounts at a time and are considered one of the least overgrazing ruminants, unlike sheep and goats. Camel meat can consequently be produced economically compared with other competing meats. According to FAOSTAT, the worldwide meat production has been projected to be double by 2050 ( http://faostat3.fao.org), due mainly to the increase in production and consumption, which is likely to intensify the freshwater crisis in the future. The virtual water content (VWC) for camel production has not been well investigated. Quantification of VWC for the camel production plays an important role in understanding the aspects of national water footprint (WFP) in arid regions and is highly needed to guide the allocation of livestock farming and optimize water use ( Qasemipour and Abbasi 2019). However, the current changes in camel farming practices (the Bedouin system based on camel mobility called “ *H’mil*” *versus* semi or intensive systems) based on intensification of the management could modify this conception. According to Chowdhury *et al*. (2017), the unit VWC of camel is 3.5- and 1.7-times the VWC for sheep and cow, respectively. One kg of cow and camel meat required 19.7 and 14.6 kg of feed supply, respectively. To my knowledge, there are no works on the carbon footprint and on the water consumption required for the production of 1 kg of camel meat. However, Faye (2013) found that the water consumption for producing 1 L of camel milk was multiplied by nine passing from 938 to 8601 liters of water per liter of produced milk. However, all these results remain to be confirmed by future other studies in different arid and semi-arid regions.

### The economic potential of camel meat

Generally, in developed countries, meat production has significant impact on nearly all aspects of the environment, including climate change ( https://ourworldindata.org/environmental-impacts-of-food). Beef is the most popular meat widely produced in the world. Unfortunately, it is also the most inefficient animal to produce meat in terms of the amount of input needed to produce it ( Paris *et al*. 2022). Camel is considered a fundamental pillar of the national economy and food security for many countries in arid regions ( Faye, 2013). Meat professionals see the still virgin camel market as a sure opportunity to do good business and participate in the development of rapidly changing agricultural and agri-food sectors. Camel meat has reduced production costs because camels are usually reared in arid regions. In these harsh environments, poor quality feed is the only source for camels and is not utilized by other domesticated species. As a result, camels can produce meat at a lower cost compared to cattle, goats and sheep. FAO projects the global meat production to more than double from 229 million tons in 1999 to 465 million tons in 2050 ( http://faostat3.fao.org). The average meat consumption worldwide during 2015 was 42.14 kg per capita/year, while in 2019 it was 43.16 kg per capita/year. Thus, the increase in consumption between these years was 2.42%. In the total meat consumption was added beef, sheep, pig, goat, poultry and then other meats (camels, rabbits…). Meanwhile, the global population is also expected to further increase. This will drive up total worldwide meat consumption. Producing more meat to meet the problem of population growth means more animal feed will have to be produced, which also means more land and water will be needed. On the basis of these expected challenges, camel meat is presented as a potential substitute for other red meats because, among other things, camels require fewer resources in terms of land and water.

### Camel meat demand in high-value export markets

Recently, more attention has been paid to the nutritional value of camel meat, with the aim of creating additional value for various camel meat products. Although the marketing systems for camel meat are not well organized. This causes failure in appreciating the importance of these products in contributing to the development of the camel sector. Camel milk, on the other hand, has been marketed and used as a processing aid in several European countries, in particular the Netherlands, Denmark and England. This culminated in 2013 when the European Community authorized the import of camel milk from the United Arab Emirates. This great popularity of camel milk is probably due to the prior knowledge of its nutritional value and human health benefits compared to the more widely consumed cow's milk.

The world’s Muslim population is expected to increase by about 35% in 20 years (2010–2030), by 2030, the global Muslim population is expected to reach 2.2 billion people compared with 1.6 billion in 2010. This huge increase in the Muslim population, coupled with the recent increase in popularity of camel meat in Australia and China, creates an unprecedented potential for camel meat.

## Recommendations

A valid alternative to beef and other red meats could be the camel meat. North African countries are the largest African producer of camel meat and derivatives. The high nutritional properties of camel meat make it suitable for inclusion in the Mediterranean diet in order to adapt it to the needs and conditions of the population. Consumers tend dislike camel meat because they associate meat with the camel itself, and this is often one of the reasons for this disapproval. Under these circumstances, it would be ideal for manufacturers to avoid using promotional labels that show the image of the camel itself. This problem could be addressed through commercial advertising and education. It empowers consumers, helping them acquire the skills and attitudes they need to be able to gear the choices they make to their economic and health interests. Australians have succeeded in promoting kangoro meat and lessons can also be drawn from the same approaches. Consumers describe camel meat as tough even though they agree that it is no different from beef in terms of flavor. The meat consumption behavior by consumers will contribute to the development of livestock camel sector. However, almost all research has proven that the older a camel, the tougher the meat. Research has established, however, that it is generally accepted that younger animals produce more tender meat than older animals. Therefore, age is an important factor in determining the quality of camel meat. There are potential opportunities for camel meat industry through brand development. Future camel research should focus on exploiting its meat potential in the same way as dairy through interdisciplinary research on efficient production systems, improved meat technology and marketing. Finally, the consumer dietary for lower or no meat consumption (e.g. vegetarian or vegan diets) or for cultured meat are assumed to expand slowly and to be adopted by a small part of population concentrated mainly in rich countries, and therefore hardly affect meat consumption over the next decade ( Chriki and Hocquette, 2020).

## Conclusions

**Figure 4.**
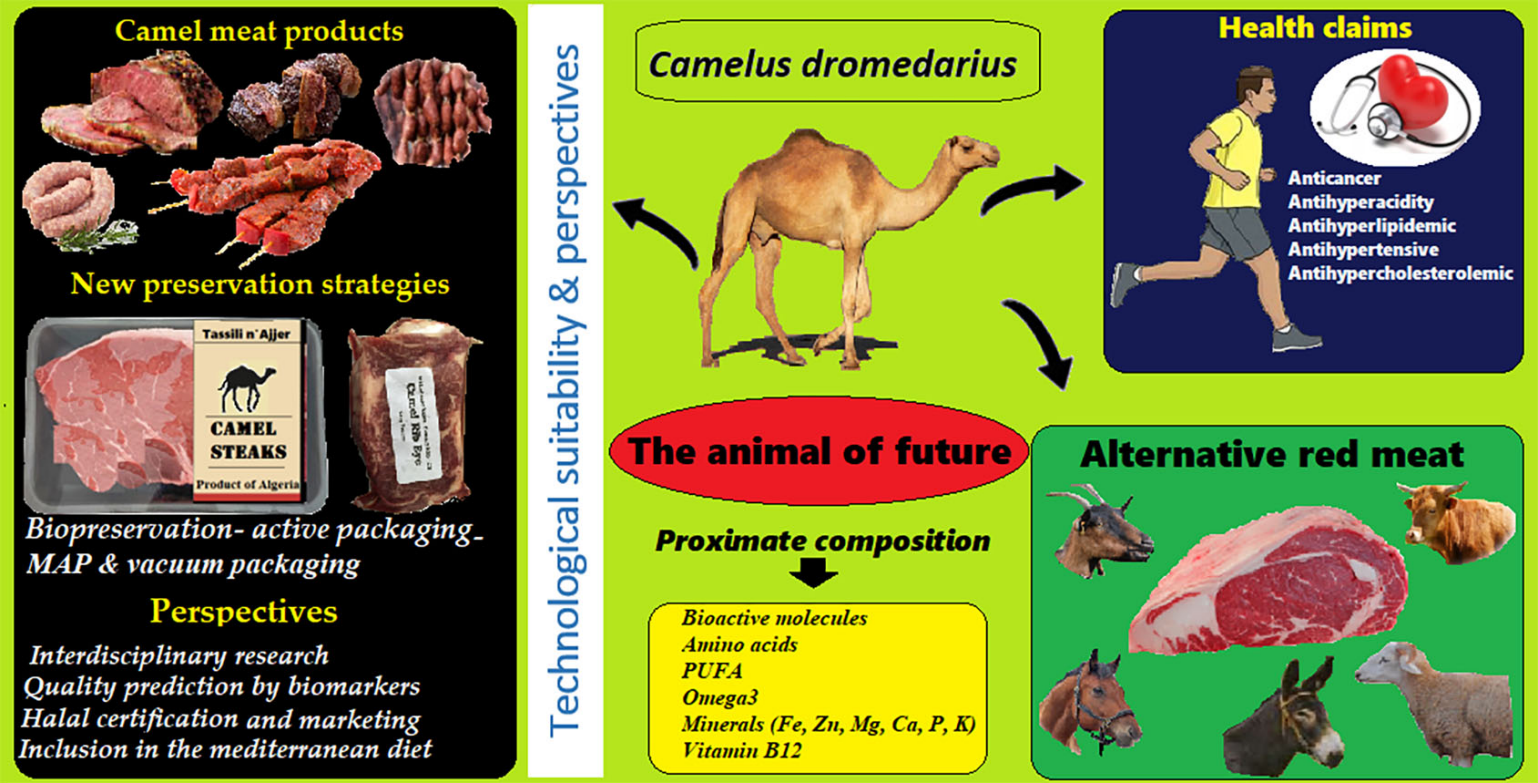
Camel is the sustainable food for the future.

Camel is the animal of the future. Without a doubt, the camel can be a tool to fight against the future challenges of climate change and their consequence on the earth. For these medicinal and nutritional benefits, camel meat can be a great option for sustainable meat worldwide supply, which represents a strong commercial argument to confirm the healthy nature of this product ( Figure 4). Knowledge about camels was traditionally restricted to limited geographical areas, particularly Middle East, Asia, and Africa, the use of camel’s products as a nutrient and for its health benefits is now known worldwide. From an economic profitability point of view, camel meat has a competitive advantage over other meats due to low production costs. However, in camel meat, the information on bioactive compounds is limited and this needs further study to identify other bioactive compounds of interest and compare them with those of other red meats. Camel meat has been processed into limited camel products to add value. It is also interesting to draw the attention of researchers from arid and semi-arid countries as well as stakeholders including decision-makers in these countries to the urgency of assessing or identifying the health risks associated with consumption of camel products and thus take the necessary measures to reduce these risks. In the light of this review, the need to implement a control strategy is obvious. The dynamics of infectious diseases in camels are highly variable. Veterinary laboratories should be provided with accurate diagnostic tools in order to detect infected animals that represent potential reservoirs of infection at an early stage and especially during seasons with abundant vectors.

Camel meat, which is produced naturally and biologically, would occupy important place on the global market. Though the scientific research achievements are modest, they open new up horizons for the modernization of the sector in order to improve the productive performances of camels in arid regions. Suitable chilling and innovative packaging technologies, are required to augment hygienic meat production and could lead to the development of expanded markets for camel meat not only in the Middle East and North Africa but also globally. Slaughterhouse solid waste management and effluent treatment are some of the parts that need better technological involvement.

## Data availability

There are no underlying data associated with this article.
