# The one-humped camel: The animal of future, potential alternative red meat, technological suitability and future perspectives

**DOI:** 10.12688/f1000research.125246.3

**Published:** 2024-07-05

**Authors:** Djamel Djenane, Mohammed Aider

**Affiliations:** 1 Laboratory of Meat Quality and Food Safety, Department of Meat Science and Technology., University of Mouloud MAMMERI, Tizi-Ouzou, 15000, Algeria; 2 Department of Soil Sciences and Agri-Food Engineering, Université Laval, Quebec City, QC, Canada; 3 Institute of Nutrition and Functional Foods (INAF), Université Laval, Quebec City, QC, Canada

**Keywords:** Camelus dromedarius; future food; health claims; alternative red meat, Comprehensive review, Health food

## Abstract

The 2020 world population data sheet indicates that world population is projected to increase from 7.8 billion in 2020 to 9.9 billion by 2050 (Increase of more than 25%). Due to the expected growth in human population, the demand for meats that could improve health status and provide therapeutic benefits is also projected to rise. The dromedary also known as the Arabian camel, or one-humped camel ( *Camelus dromedarius*), a pseudo ruminant adapted to arid climates, has physiological, biological and metabolic characteristics which give it a legendary reputation for surviving in the extreme conditions of desert environments considered restrictive for other ruminants. Camel meat is an ethnic food consumed across the arid regions of Middle East, North-East Africa, Australia and China. For these medicinal and nutritional benefits, camel meat can be a great option for sustainable meat worldwide supply. A considerable amount of literature has been published on technological aspects and quality properties of beef, lamb and pork but the information available on the technological aspects of the meat of the one humped camel is very limited. Camels are usually raised in less developed countries and their meat is as nutritionally good as any other traditional meat source. Its quality also depends on the breed, sex, age, breeding conditions and type of muscle consumed. A compilation of existing literature related to new technological advances in packaging, shelf-life and quality of camel meat has not been reviewed to the best of our knowledge. Therefore, this review attempts to explore the nutritional composition, health benefits of camel meat, as well as various technological and processing interventions to improve its quality and consumer acceptance. This review will be helpful for camel sector and highlight the potential for global marketability of camel meat and to generate value added products.

## Introduction

The one-humped camel breeding represents an important activity for arid and semi-arid regions. The good adaptation of this species to arid conditions is due to its strength and docility. On the one hand, it is an ideal animal which could be used for multiple purposes and, also it is the unique animal which survives and reproduces under such harsh climatic conditions which is not suitable for the survival of other types of domestic mammals on the other hand.

The demand for information on the nutritional compositions of meats is growing and unfortunately, in many arid and semi-arid countries there is little expertise in camels. The “Camelology” had a relatively prosperous period during the colonial periods, thanks to the work carried out by European veterinarians hired on the spot. And after their independence, research institutions and development organizations neglected camel breeding for a long time, for various reasons. Cattle were preferred because they seemed more “modern”: the decline in caravan activity considerably reduced the interest in dromedaries.

The low importance of the consumption of camel meat is linked to the variability of its organoleptic qualities. Camel meat is considered a very tough compared to other meats from other domestic animals. The lack of good quality products can in part be attributed to the fact that camel meat is often obtained from aged males (♂) and females (♀) which have become less efficient.

Generally, red meats are a valuable source of vital micro and macro-nutrients, particularly of bioavailable iron (Fe) and zinc (Zn), proteins, trace elements and B vitamins, especially vitamin B12. Actually, the current enthusiasm for the consumption of camel meat as red meat is based in part on the therapeutic or medicinal virtues that are attributed to it. It is considered lean because it comes from animals fed on natural pastures, and responds to the strong “health claim” trend, which consists of limiting the consumption of red meat and animal fat. Furthermore, in arid regions, the camel is likely to produce animal protein at a comparatively low cost ( Farouk and Bekhit 2013).

Today, multidisciplinary research is carried out in many countries on camel meat for the adoption of efficient production systems, the improvement of its transformation as well as its marketing. The countries concerned must not remain on the sidelines of this renewed interest. For breeders, butchers, and, possibly, manufacturers located in arid regions, the promotion and preservation of the quality characteristics of camel meat constitute a major challenge for camel sector. In recent years, various researchers have implemented new alternatives preservation techniques to maintain the quality of camel meat during storage ( Djenane *et al.* 2020). This strategy would extend shelf-life while ensuring product safety during distribution and subsequent retail display.

The camel sector is currently facing a gradual opening of borders to products of animal origin. These come from countries where technical and regulatory developments have made it possible to promote the meat industry and improve product quality. The camel sector must adapt to the transition towards a competitive economy to design and implement liberalization reforms in a context marked by agreements concluded between the countries. Veterinary control focuses more on animal health compliance for healthy consumption. Despite the efforts made by the veterinary services to ensure safe meat, hygiene conditions remain insufficient ( Yehia *et al.* 2020, 2021).

This review discusses the nutritional and qualitative properties of camel meat, and explains its versatility and positive effects on human health. The current “health claims” situation of this product and the development prospects through a few technological suitability in progress, and future perspectives are also discussed. In addition, this review brings additional elements which I seem wise to summarize here. In addition to the knowledge acquired on camel meat, in view of the most recent works, the current market situation for this product and development prospects will be presented.

### Camel numbers and distribution

The genus *Camelus* is one of the animals mentioned in the Holy Quran “Do they not then look at the camels how they are created (verse17)”: (Surah 88/Al-Ghachiyah), and provided huge economic assets ( Saeed *et al.* 2015). The name of dromedary comes from the Greek “ *dromos*” which means road. Without the presence of camels, many places on the planet would be uninhabitable for humans. Although camel is not native to Europe, but its skeletons have been found at several sites in different Roman provinces, dating from the early 1 ^st^ to 4 ^th^ century AD ( Pigière and Henrotay 2012; Sophie *et al.* 2020). Obviously closely linked to the conquests, especially those of Islam during its expansion to northern Africa, the dromedary was found in southern Spain and Sicily (Italy) throughout the Middle Ages. However, alongside its early warlike use, numerous archaeological testimonies attest to its ancient dairy use. Its strength and docility makes it an ideal animal that could be used for multiple purposes. The image of the dromedary represents a symbol of the survival of man in the desert, which remains attached to the history of the great nomadic civilizations of dry and hot regions, characterized by a long unfavorable period, often greater than eight (08) months, and by rare and weak precipitation. Moreover, *C. dromedarius* is a homoiothermal organism perfectly adapted to extreme conditions of arid zone ( Saeed *et al.* 2015). Many researchers studied and characterized the heat shock proteins ( *Hsps*) including other molecular chaperones and cytoprotective protein of camels ( Saeed *et al.* 2015). Ataya *et al.* (2014) identified camelid genes of antioxidant and detoxification enzymes and their expression that may lead to a better understanding of how camels adapt best to desert conditions. The camel genome is approximately 2.38 Gb in size, and contains more than 20,000 genes ( Amanat *et al.* 2019). One of the best-known characteristics of the camel is its ability to live for long periods without drinking. Its eating behavior is particular. Indeed, its digestive physiology is entirely oriented towards the valuation of fodder with low nutritional value. Camel is a polygastric animal, but it is often referred to as “ *pseudo-ruminant*”. There are two species of camel within the genus *Camelus* of the family Camelidae. The dromedary one-humped camel ( *Camelus dromedarius*) is most widely distributed in the hot arid areas of the Africa and Middle-East. The two humped Bactrian camel ( *Camelus bactrianus*) is found in parts of China and Central Asia. There have been some attempts for crossing between the dromedary and Bactrian ( Ebadi, 2015). Despite advances in molecular biology to specify parentage and identify relevant genetic indicators, camel genetics are in their infancy ( Schulz *et al.* 2010; Mahmoud *et al.* 2020). Consequently, the conservation of local camel breeds consists first of all in making a real inventory of them using modern tools ( Nowier *et al.* 2020). The problem of genetic erosion could arise in the case of *C.* *dromedarius* × *C.* *Bactrianus* hybridization in central Asia, a practice to improve milk production. However, reproductive efficiency in camels under extreme arid conditions would be low due to precarious production conditions, traditional livestock management and the low genetic potential of native camel ( Gherissi *et al.* 2020). Because camels are migrant animals, the number of dromedaries is not known with precision given the drought episodes and the population movements that accompanied it. Numerous events such as the politico-military disturbances and epizootic that have occurred since the year of the census have been able to modify the theoretical evolution of the numbers. And often, the official figures were underestimated compared to reality. World camel population exceeds 35 million herds, according to latest statistics (Faye, 2015). *Camelus dromedarius* is the most frequent and widespread domestic camel species composing 90% of the total camel population ( Mohandesan *et al.* 2017), whereas the two-humped Bactrian camel ( *Camelus bacterium*) represents the remainder (10%). In addition, the Food and Agriculture Organization of the United Nations (FAO) figures do not take into account the wild Australian dromedaries which, according to various estimates, number 1.5 to 2 million head. The slaughter networks play a major role in health control, and better control of flows to regulate the market. A significant number of camels are slaughtered outside official control networks, possibly indicating an underestimation of production. This number is probably underestimated, especially in Sahelian and North African countries (Mauritania, Mali, Niger, Chad, Sudan, Ethiopia and Algeria). Illegal slaughter is often a common practice of occasional butchers who offer uncontrolled and cheaper meat for sale from unregistered animals.

The development of peri-urban camel breeding for both meat and milk supply has disrupted the ancient links between nomads and sedentary people, resulting in an increased interpenetration of the activities carried out by both. The desert regions of the planet, like other global ecosystems, are under strong pressure from urbanization ( Tahir and Huang 2020). The urbanization of oases is such that geographers now speak of “desert cities” rather than real oases. Sustainability issues are of particular importance for the camel sector in arid countries ( Faye 2016), where 35 million camels are raised, ensuring livelihoods for vulnerable populations in arid areas, including those in marginal areas despite the fact that one carries with him the image of an activity opposed to modernity.

Australia plays a special role, as much of the camel herds are kept in the wild. An environmental problem has arisen because of an abundant population that is continuously growing. To solve the problem, the Australian authorities opted for the mass destruction of wild camels. However, another suggested route was the promotion of the meat of this species on local and international markets ( Sultan *et al.* 2018; Brisbane 2021).

It would seem that the exploitation of dromedaries has been largely excluded from development projects in arid and semi-arid countries. However, many works have been carried out in recent years by scientists from the countries concerned and testifies to their awareness of the importance of the camel in their own region with the collaboration of international support and funding for research and development ( Djenane *et al.* 2020; Gagaoua *et al.* 2021a, b). Nevertheless, it is evident that there is now some acquired competence in this area.

### Characteristics and composition of camel meat

Beyond the variability observed according to breeds or types of animals, age, sex, breeding conditions, seasons, type of muscles, sampling and analysis methods, the camel meat composition has been fairly well studied in different countries and therefore an average composition can be reported ( Abdelhadi *et al.* 2012; Al-Owaimer *et al.* 2014; Suliman *et al.* 2019). The literature reported that chemical composition of beef has similar to camel meat (water 72.1%, protein 21.1%, fat 2.7% and ash 0.5%) ( Osaili *et al.* 2020b). Therefore, from a nutritional point of view, camel meat could be a viable alternative source to livestock.

The camel meat water content (70 to 77%) is comparable to that of other farmed species ( Al-Owaimer, 2000; Kadim *et al.* 2006a; Mohammed *et al.* 2020). The dromedary is also an appreciable source of protein, its meat containing between 17 to 23% according to some sources ( Kadim *et al.* 2008; Abdelhadi *et al.* 2017). In general, camel meat is similar or maybe a better source of essential and non-essential amino acids than meats of other species such as beef, lamb, and goat ( Raiymbek *et al.* 2015; Kadim *et al.* 2011). Minerals represent between 1.1 and 1.5% of camel meat ( Al-Owaimer, 2000; Kadim *et al.* 2006a). Other study revealed that camel meat has higher vitamins content but lower total fat and cholesterol than other meats from other species such as mutton, beef, and chicken ( Mohamme *et al.* 2020). Some data concerning the vitamin content are also available ( Ulmer *et al.* 2004): 0.12 mg/100 g for thiamine (B1), 0.18 for riboflavin (B2), 0.25 for pyridoxine (B6) and 0.61 for α-tocopherol (vitamin E). There is limited information available on the vitamin B12 levels found in camel meat. Mohammed *et al.* (2020) and Kadim *et al.* (2006a, 2008) reported that camel meat is an appreciable source of vitamin B12 (0.62 mg/100g) and iron (Fe) (45.5 mg/100), which represents a strong commercial argument to affirm its healthy character. For our nutrition, meat and offal from ruminants are a potential source of vitamin B12 ( Williams, 2007). In these species, this vitamin, strictly of microbial origin ( Oh *et al.* 2021), is synthesized in the rumen before being absorbed and stored in the liver. Vitamin B12, a water-soluble vitamin, is the unique vitamin among the other water soluble vitamins that can be stored in the liver. Considering the importance of this vitamin in human nutrition, its deficiency leads to serious health concerns. Hence, the valuable content in red meat such vitamin B12, also known as cobalamin, cyanocobalamin or also called the pernicious antianemia factor, could be affected by meat processing. It is thus important to identify the production and technological factors which alter (increase or decrease) the vitamin B12 content of meats. Finally, the polyunsaturated fatty acids (PUFAs) composition in camel meat represents nearly 8% of the total fatty acid (FA) content, this can make camel meat susceptible to oxidation during storage ( Maqsood *et al.* 2015a, b).

### Proteins and amino acids

The dromedary is an appreciable source of protein, its meat containing between 17 to 23% according to some sources ( Abdelhadi *et al.* 2017; Kadim *et al.* 2011; El-sharawy *et al.* 2018). Meat from young camels has similar protein content to those found in other animals ( Kadim *et al.* 2013). Suliman *et al.* (2019) found greater differences in protein contents between various camel muscles. In contrast, Kadim *et al.* (2006b, 2013) reported slight differences between various muscles and different age camel groups. To further improve the protein content of camel meat, Ebadi (2015) crossed ( *C.* *dromedarius* × *C.* *bactrianus*) and found a clear improvement in protein levels by this crossing. The composition of the amino acids is an important criterion for assessing the nutritional quality of meats. In general, camel meat is similar or maybe a better source of non-essential amino acids than meats of other species such as beef, lamb, and goat ( Dawood 1995; Kadim *et al.* 2011). The aspartic and glutamic acids are the major non-essential amino acids in camel meat ranged from 11 to 19% of protein, respectively ( Dawood and Alkanhal 1995; Kadim *et al.* 2011). Arginine, alanine and proline have also been described as the highest non-essential amino acids in camel meat ( Abdelhadi *et al.* 2017). Regarding the essential amino acid, camel meat contains a relatively high proportion of lysine, leucine, threonine, methionine and histidine ( Raiymbek *et al.* 2015; Abdelhadi *et al.* 2017). Al-Shabib and Abu-Tarboush (2004) found that the concentration of tryptophan was 1.8% in camel meat, which was higher than the 1.3% reported for beef ( Kadim *et al.* 2008). Therefore, it could be an excellent source of protein of high biological value. In camel meat, the ratio of essential to non-essential amino acids is 0.85 ( Abdelhadi *et al.* 2017), very similar to that reported in beef, lamb and goat (0.86, 0.83 and 0.90, respectively) ( Elgasim and Alkanhal 1992; Dawood and Alkanhal 1995). According to Dawood and Alkanhal (1995), the essential amino acid content of camel meat is not affected by the type, sex or age of the animal. Abdelhadi *et al.* (2017) reported similar contents of essential and non-essential amino acids in female (♀) than in male (♂), except for arginine considered higher in ♀. On the other hand, differences between 0.5 and 9.5% were observed depending on the type of muscle ( Elgasim and Alkanhal 1992; Dawood and Alkanhal 1995). Certain essential amino acids increase in muscle with the age of the animal, but also during heat treatments ( Schonfeldt *et al.* 2010; Sakomura *et al.* 2015). To my knowledge, no studies regarding the effect of various technological factors on the amino acid composition of camel meat have been published. This makes it possible to increase research in this area to preserve a better nutritional quality of this meat.

### Water

The moisture content widely varies in camel meat (63.0 to 79.0%) ( Kadim *et al.* 2006a; Maqsood *et al.* 2015a). This difference between the maximum and minimum moisture contents is due to the age of animals, production system, cut, pH, and season ( Kadim *et al.* 2006a; Suliman *et al.* 2019; Echegaray *et al.* 2021). The water content of meat is inversely related to fat content ( Echegaray *et al.* 2021), whereas it is unaffected by protein content. In the camel meat, variation in expressed juice between *Longissimus thoracis*, *Infraspinatus*, *Triceps brachii*, S *emitendinosus*, *Semimembranosus*, and *Biceps femoris* muscles ranged from 28.5 to 33.5% ( Kadim *et al.* 2013). These differences may be due to position of muscle, its activity, fiber types, pH, intramuscular fat and the ratio of water to protein of muscle. However, Kadim *et al.* (2013) and Gheisari *et al.* (2009) reported that muscles within the same camel carcass appear to have similar moisture contents. Moisture content usually plays an important role in maintaining the quality characteristics of meat, its processing potential, affects its shelf-life and has strong impact on the sensory parameters and nutritional values ( Leygonie and Hoffman 2020). To study the qualitative stability of camel meat during storage, Mohammed *et al.* (2020) measured changes in the moisture content of different meat samples. The results indicated that moisture content of beef, lamb and chicken changed slightly after 7 days of storage. However, there was no obvious change in the moisture content of camel meat during the same period of storage.

### Fat and fatty acids

#### Fat

According to the scientific literature, the fat content of camel meat ranges from 1.4 to 7% ( Dawood and Alkanhal 1995), and up to 10.5% in older animals ( Kadim *et al.* 2006a). Fresh camel meat is composed of 3.2% fat ( Khezrian and Shahbazi 2018). In another study, a fat content in Arabian camel meat was examined by Al-Owaimer *et al.* (2014). These authors reported that the fat meat content was 1.14 to 4.63%. In a comparative study, Gheisari and Motamedi (2010) reported that the camel meat fat content (= 8%), higher than beef (5.35%) and poultry meat (4.2%). Maqsood *et al.* (2015a) found that camel meat contains ≃ 5% of total fat. Kadim *et al.* (2006a), El-sharawy *et al.* (2018) and Osaili *et al.* (2020b) found ≃1.3% and confirmed that camel meat could be much leaner than meat produced by other species especially if it is slaughtered at a young age. However, the maximum value 10.5% of fat recorded in camel meat indicates that older animals deposit more body fat. This implies that the meat industry should target young camels (1-3 years old) for the production of premium meat. These differences could be attributed to the muscle type and feeding systems, which are suggested by Pethick *et al.* (2021) who stated that fat content varies depending on species, origin, feeding system and the cut. It is also important to point out that camel meat is low in intramuscular fats (3.3%) ( Maqsood *et al.* 2016a, b). However, it can vary considerably. Kadim *et al.* (2006a) and Al-Owaimer (2000) reported a value of 6.4% and 5.2%, respectively, which is comparable to the 7% reported by Dawood and Alkanhal (1995).

#### Fatty acids

Fatty acids (FAs) are currently the subject of an abundant literature. Their qualifier of “polyunsaturates”, “omega 3” are all terms with positive connotations used in human health. The FA composition of meats is of great importance as it is associated with human health ( Roopashree *et al.* 2021). Reduction of saturated fatty acid (SFA) intake is very important to prevent human health diseases such as obesity, hypercholesterolemia and to decrease the risk of cancer ( Lunn and Theobald 2006). Often in the scientific literature it is indicated that animal fat is composed mainly of unsaturated and SFA, headed by oleic (C18: 1n-9), palmitic (C16: 0) and stearic acid (C18: 0) ( Juárez *et al.* 2021). Beyond the variability observed according to breeds or types of animals, age, sex or breeding conditions, the composition of camel meat has been fairly well studied in different countries. The main reason for the difference in camel meat composition might be due to animal age, nutrition, genetics, breed and muscle location ( Mamani-Linares and Gallo 2014; Suliman *et al.* 2019). Mamani-Linares and Gallo (2014) studied the effect of diet on fatty acid composition in camelid’s and reported an increase in linoleic (C18: 2n-6) and linolenic (C18: 3n-3) acids in the animals receiving hay supplement. In other hand, Suliman *et al.* (2019) reported that fat content was higher in the Pakistani than in the Baladi Saudi and Somali breeds. The comparison by sex and race revealed very significant effects on the parameters studied ( Sahraoui *et al.* 2014). Thus, the contents of FA, SFA, monounsaturated fatty acids (MUFAs), PUFAs, Omega 6 (ω6) and Omega 3 (ω3) are higher in Sahraoui male. However, Sahraoui and Tergans females exhibited significantly higher PUFA/SFA and ω6/ω3 ratios (deemed favorable) than male but also a fairly low fat content ( Sahraoui *et al.* 2014). These results suggest overall better nutritional characteristics, mainly in female.

Abdelhadi *et al.* (2017) found that PUFA/SFA ratio in camel meat was closer to 0.45 which is recommended value for human nutrition. Also, the ω6/ω3 ratio was < 4.0 which is recommended value for human diets ( Abdelhadi *et al.* 2017). Similarly, Sahraoui *et al.* (2014) and Rawdah *et al.* (1994) found a ratio of the PUFA/SFA ratio equal to 0.25 and that of ω3/ω6 equal 0.1 to 0.24. Kargozari *et al.* (2014) obtained an n-6/n-3 ratio = 6.22 in sausages made with beef, compared to 2.95 in those made with camel meat. This indicates that camel sausages fit perfectly into the recommendations for this ratio. The cholesterol content in camel meat was reported at 57.56 mg/100 g (≈20% of the recommended daily intake less than 300 mg) ( Mohammed *et al.* 2020). According to a study by Sahraoui *et al.* (2014), camel meat contains 54.6% SFAs, 35.0% MUFAs and 10.4% PUFAs. Regarding PUFAs, arachidonic (20:4 ω-6), docosahexaenoic (22:5 ω-3), eicosapentaenoic (20:5 ω-3) and linoleic acid (18:2 ω-6) were the major PUFAs in camel meat ( Abdelhadi *et al.* 2017). Similarly, Maqsood *et al.* (2015a) and Kadim *et al.* (2013) found that camel meat was dominated by SFAs with considerably high amounts of unsaturated fatty acids (UFA): Content of total SFAs and unsaturated fatty acids (UFAs) were 58.46 and 41.5 mg/100 g, respectively. As a limiting factor, PUFAs are sensitive to oxidation reactions and can play a major role in the oxidative deterioration of stored meats. SFAs in camels of both sexes were dominated by myristic (14:0), palmitic (16:0) and stearic (18:0) acids and to a lesser extent by pentadecanoic fatty acid (15:0) ( Abdelhadi *et al.* 2017). Similar trends have been reported by Kadim *et al.* (2011). The SFAs in meat, in particular lauric (12: 0), myristic (14: 0) and palmitic (16: 0) acids, are widely considered to have negative effects on human health, as they are linked to an increased risk of cardiovascular disease ( Briggs *et al.* 2017). The results reported by Kadim *et al.* (2013) for oleic acid (26.2%) and MUFAs (37.2%) in camel *Longissimus thoracis* (LT) muscle correspond approximately to the values reported by Abdelhadi *et al.* (2017). These authors also found that SFAs in camels of both sexes were dominated by myristic acid (14:0), palmitic (16:0) and stearic (18:0) acids and to a lesser extent by pentadecanoic fatty acid (15:0). Maqsood *et al.* (2015a) reported that the dominant PUFAs in the camel meat was linoleic acid (Cis) (C18:2n6c) and found that predominant fatty acids in camel meat were oleic acid (C18:1 n-9) (32.72%), followed by palmitic acid (C16:0) (31.86%) and stearic acid (C18:0 n-6) (17%). Unfortunately, in the scientific literature, there are no reports comparing FAs of meat of different species in the same study and since diet plays an important role in modulating the fatty acid profile and a comparison with the literature values would not be appropriate.

### Mineral composition

Red meat is an important source of minerals, and camel meats can be considered a good source. Minerals are classified as essential elements and their deficiency can be detrimental to human health. They represent between 1.1 and 1.5% of camel meat ( Al-Owaimer, 2000; Kadim *et al.* 2006a). When compared to beef, veal, lamb and mutton, camel meat contains higher amounts of calcium (Ca) (4.7-11.50 mg/100 g). The Fe content in camel meat (1.2-3.4 mg/100 g) varied among cuts due to the different physiological requirements of myoglobin (Mb) of different muscles ( Dawood 1995; Kadim *et al.* 2009a). As with other red meat species, camel meat cuts containing oxidative muscles (e.g. leg and neck) have higher Fe content than glycolytic muscles. Similarly, camel meat is an important source of zinc (Zn) and contains about 3.07 to 4.80 mg/100 g fresh weight ( Dawood and Alkanhal 1995) but a higher percentage of variation (47-56%) has been reported in other studies ( Rashed 2002). The level of variation of Fe, magnesium (Mg), sodium (Na), phosphorus (P) and potassium (K) contents reported by various authors indicates that factors such as physiological, genetics, age and cuts can play a major role in determining the mineral contents in camel meat ( Kadim *et al.* 2013; Smith *et al.* 2017a, b). It should be mentioned that even within the same herd with a similar agricultural history, the biological variation of the elements could be high ( Kadim *et al.* 2006a).

### Ash content

Ash content in camel meat is between 1.1 and 1.5% ( Kadim *et al.* 2006a; Gheisari *et al.* 2009; Kadim *et al.* 2013) which is within the range of values reported for other animals such as beef, lamb and goat meats and varies between muscles. Suliman *et al.* (2019) reported that ash content was higher in the Pakistani than in the Somali and Baladi Saudi breeds. Gheisari *et al.* (2009) found that age had a significant effect on camel meat ash content, while Kadim *et al.* (2006a, 2008) found no effect of age.

Variation in nutritional composition of camel meat such as effect of Age at which the camel is slaughtered plays a significant role in determining the meat’s composition and in turn, affects consumer acceptance. Also, the nutritional composition of camel meat shows considerable diversity based on the specific muscle being studied. Different muscle portions have distinct compositions, influencing the overall nutritional profile of the meat. In addition, variations in the chemical composition of camel meat have been observed due to differences in breeds. Different breeds of camels may exhibit varying nutritional properties in their meat. Understanding these variations is essential for researchers, meat processors, and consumers to make informed decisions regarding camel meat consumption and utilization. By considering these factors, stakeholders can optimize the production and marketing of camel meat products, meeting the preferences and demands of consumers while ensuring the meat’s overall quality and nutritional value.

## Camel meat terminology and specification

Camel meat is the relatively least demanded red meat internationally ( Wu and Zhou 2021). In many cases, is viewed as a by-product of the dairy systems. It is often regarded as inferior to other meats. This could be attributed to the fact that meats from older camels are often slaughtered at the end of their productive life. However, with increasing demand for meat in terms of both quantity and quality, it is likely to stimulate the emergence of better organized livestock systems. For long periods, camel meat was considered a powerful marker of social, ethnic, and religious identities ( Baba *et al.* 2021). Thus, among the ancient sedentary people of the Middle East, it was reserved for poor people. If it is prohibited for Jews, its status remains certain in the eyes of Muslims (Halal) ( Wu and Zhou 2021). However, the definition of the terminologies and the specifications of camel meat is considered the future tool for marketing and international trade. Adopted from the beef specifications, the central Australian camel industry association Inc. (CACIA), presents the camel meat specifications at its website ( CACIA, 2022). The adoption of these specifications greatly facilitates the international trade of camel meat ( Brisbane, 2021).

Many traditional meat products characterize the countries of Africa and the Middle East. However, only a few of them have been reported and characterized scientifically. On the other hand, regional cultural differences differ greatly between these peoples and the meat products brought by these peoples will have a strong symbolic value by contributing to the sustainability and development of rural areas ( Gagaoua and Boudechicha 2018). Branded meats are becoming increasingly popular within the food-service sector. Consumers are increasingly interested in the origin of food and its production systems ( Pearson *et al.* 2011). For camel meat brand development, out-of-home catering seems to be a good indication of the added value of this product. In recent years, there has been a proliferation of points of sale offering new and very attractive products that better meet modern standards. This shows that the consumption of camel meat is no longer the identity expression of a social group. The role of out-of-home catering in the development of the sector has already been underlined by several authors. The low meat production performance of the camel in its natural environment is mainly due to the poor feeding behavior of the young before weaning ( Kadim *et al.* 2008). Overall, for the camel sector the most relevant product in economic terms is milk, followed by meat products ( Abrhaley and Leta 2018). Meat production systems are poor organized to produce quality meat. However, Intensive “feed-lot” type systems based on a diet rich in concentrates are still underdeveloped, with the exception of Tunisia where fattening workshops supply the market with animals weighing 250 kg less than 2 years old ( Bessalah *et al.* 2016). The inadequacy of livestock production that most arid areas have witnessed in recent years is due to increased demand, climate change and a decline in feed resources. In the face of these limitations, camel farming, although marginal, can make a great credit for the sustainable development of the economy and country environment.

### Consumer perception

Although camel meat is consumed in several areas, there are taboos in some cultures and religions. Camel meat is not consumed in some developed countries (Europe, U.S.A., Canada, New Zealand) except Australia and eating camel is prohibited in the Holy Torah for Jews but not for Muslims in the Holy Coran. SWOT analysis was conducted to identify the camel meat commodity Strengths, Weaknesses, Opportunities and Threats. The results of this analysis showed that one of the identified weaknesses is the lack of consumer awareness towards camel meat ( Mbaga 2013). Often, consumers are unaware that camel meat is a healthy meat product. Consumers will not buy a product unless they have heard of it before and therefore their awareness is very important. Consumers tend to dislike camel meat because they associate meat with the camel itself, and this is often one of the reasons for this disapproval. Under these circumstances, it would be ideal for manufacturers to avoid using promotional labels that show the image of the camel itself. This problem could be addressed through commercial advertising and education. It empowers consumers, helping them acquire the skills and attitudes they need to be able to gear the choices they make to their economic and health interests. Australians have succeeded in promoting kangaroo meat and lessons can also be drawn from the same approaches ( Australian Government, 2020).

Consumer motivations and barriers for buying camel meat were further investigated via the survey approach. Previous study is available in the literature regarding small ruminants’ meat consumer preferences ( Brahimi *et al.* 2020). This study was designed to deepen the understanding of consumers’ perceptions of different meats, as well as the motivations and barriers faced by consumers when approaching these products. Consumers often mentioned price as an important quality attribute. The health aspect also emerged among participants. Some consumers believed that camel meat had a lower fat content compared to other meats, and was particularly suitable for people with diseases. However, all of the consumers perceived camel meat as not expensive, and for this reason as suitable for daily consumption. Reducing prices could be considered a good strategy to increase the consumption of camel meat among the population. Further studies are needed to confirm these findings and to explore the antecedents of these attitudes in larger samples in different countries and a possible approach for growing the consumption of this meat would be to communicate the benefits related to its consumption, and to offer more detail on food labels.

## Camel meat as a substitute for other red meats

**Figure 1.**
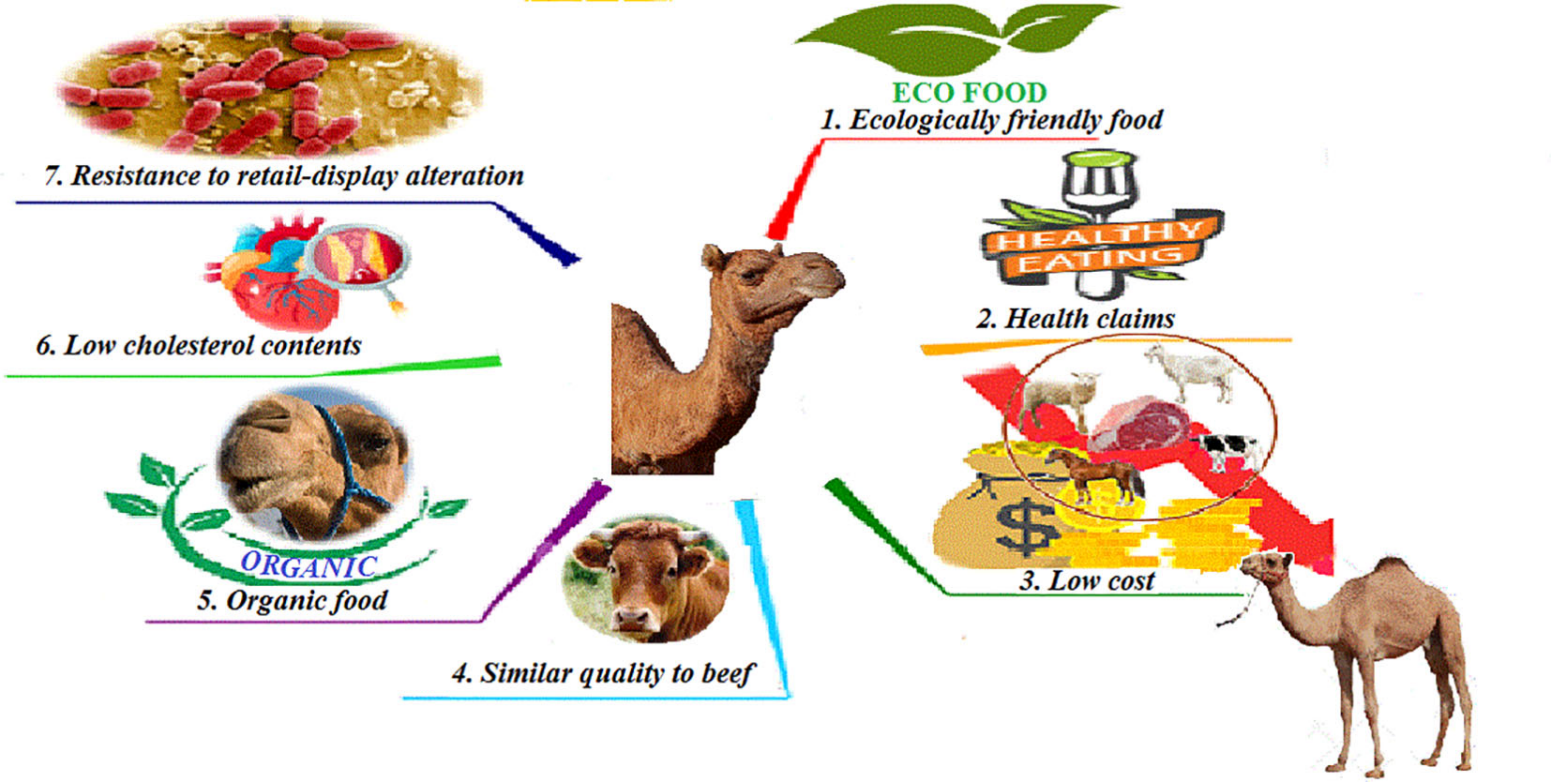
Camel meat as a substitute for red meats.

One of the major challenges for global food security is the high demand for meat products ( CCFASR, 2015). For these medicinal and nutritional benefits, camel meat can be a great option for sustainable meat supply and its production under sustainable extensive systems should be encouraged ( Kadim *et al.* 2014; Baba *et al.* 2021). In Africa, the Middle East and some Asian countries, especially in arid and semi-arid regions, camel is regarded as a main source of animal protein that equals than other meats in commercial importance. Long ago, the production of camel meat is secondary due to the lack of beneficial outlets. Some consumers mistakenly reject camel meat believing it to be tough, difficult to digest and without much nutritional value ( Baba *et al.* 2021). However, the current development of society through modernization and increasing urbanization has caused the dromedary to lose its polyfunctionality, and has accentuated its role as an essential supplier of animal proteins for the arid and semi-arid population, the most affected by malnutrition ( Faye, 2016). This deficiency of animal proteins is most pronounced in desert areas where animal products are generally more expensive and climatic conditions hamper the efficient production of slaughter animals. The interest in examining the evaluation of the various breeding facilities available is therefore significant. Hence the one-humped camel as a meat source seems to present a viable alternative to cattle ( Figure 1). In addition, the price of camel meat is often lower than that of cattle and sheep ( Kadim *et al.* 2014), due to lower transaction costs and circuits with fewer intermediaries, for a production which is also still very extensive and therefore with few inputs. This allows access to meat proteins for the often most disadvantaged populations and ensures camel meat a certain competitiveness. In a recent study, Brahimi *et al.* (2020) confirmed that the consumption of camel meat is less efficient in its environment (Algerian northern Sahara). These authors suggested in-depth studies on the sector to identify the bottlenecks invalidating the promotion of the consumption of camel meat. Like all Algerian Muslim families, those in the southern regions of the country sacrificed the sheep during Aïd El-Adha ( Feast of sacrifice). However, several of them have fallen back on the sacrifice of the camel, locally called “El-Hachi” or “El-Houar”. This is not only valid for camel, but also for beef. These are animals eligible for sacrifice, according to the Islamic Shari’a Council, the sacrifice can only be made by seven (07) people for one (01) camel or beef.

The general qualitative characteristics of camel meat are very similar to those of beef. Moreover, it is difficult for an uninformed consumer to tell the difference ( Suliman *et al.* 2020). It has indisputable dietary advantages due to its low cholesterol content, and its relative richness in mono and PUFAs, vitamins and calcium ( Baba *et al.* 2021). However, the sector still appears to be poorly organized as a whole to make it possible to make meat of high taste quality available to consumers ( Brahimi *et al.* 2020). During the last decades, the world consumption of camel meat is increasing and the main “camel meat eaters” with more than 3 kg/habitant/year are in Emirates, Mauritania, Mongolia, Oman, Somalia and Western Sahara ( Faye and Bonnet 2012). The cholesterol content of camel meat (50 mg/100 g) is lower than that of other farm animals ( Raiymbek *et al.* 2015). It appears that camel meat can be used as a substitute for beef due to its reduced cholesterol content and is a strong commercial argument for emphasizing the healthy character of this product. However, the variability of the cholesterol content between different muscles is depending on various factors related to the animal (age, sex, dietary factors, etc.) or to the methods of analysis. It is important to emphasize that cholesterol intake is minimally related to human health compared with genetic inheritance or other factors influencing health and disease. For some people, diet and lifestyle (diet rich in meat and dairy products) cause high cholesterol, but for others, high cholesterol is genetic: It runs in the family: called familial hypercholesterolemia (FH), relatively common and constitutes a serious public health concern such as cardiac problems ( Dlouha *et al.* 2021). With the ancestral breeding methods, the meat producing ability of camels is limited by modest growth rates (500 g/day) ( Kadim *et al.* 2008). On the one hand, the application of new technologies used for cattle meats are also being considered for evaluating camel meat (fiber optic spectrophotometry, video image analysis, probes optics, electromechanical probe) ( Craigie *et al.* 2012; Aït-Kaddour *et al.* 2020; Dlouha *et al.* 2021). In other hand, emerging meat-processing technologies (High-pressure processing, electronic nose, Ohmic heating, pulsed electric field …) are some of these new technologies perfectly applicable to the meat of camelids ( Varghese *et al.* 2014; Smetana *et al.* 2019; Sarno *et al.* 2020; Bhat *et al.* 2019). Similarly, the search for genetic markers that predict beef quality ( Picard *et al.* 2019; Sun *et al.* 2022) may be adopted in camel meat in the near future.

Since the horse meat scandal, awareness of food fraud has risen sharply. However, with an increased demand for camel meat, camel meat-related food products are susceptible to food fraud ( Zhao *et al.* 2020). These food frauds can be detected in a very specific way and relatively quantified thanks to real-time polymerase chain reaction (PCR)-lateral flow immunoassay (LFI) (making it possible for example to determine the percentage of each animal species in relation to the total amount of meat). Overall, this new method could be ideal for government laboratories to detect food fraud of this kind.

A valid alternative to beef and other red meats could be the camel meat. North African countries are the largest African producer of camel meat and derivatives. The high nutritional properties of camel meat make it suitable for inclusion in the Mediterranean diet in order to adapt it to the needs and conditions of the population.

## Is there ecological harmlessness of camel meat production?

The growing demand for meat cannot be met by conventional meat production alone, because 80% of all arable land is already used directly or indirectly for livestock production ( Ritchie, 2017) and this is unsustainable as it is, due to its large ecological footprint. By 2050, the world’s population will reach around 10 billion people, according to a new report published by the United Nations Department of Economic and Social Affairs (2019). A great challenge awaits policy makers and ensuring food security without compromising the main pillars of sustainability is one of the main objectives of the United Nations for sustainable development ( UN, 2019). In this optical, the camel sector must adopt sustainable practices in order to become more competitive ( Faye, 2013). In comparison to other species such as goats, cattle and sheep, camel is less destructive for the fragile pasturelands, thus contributing to the environmental sustainability of the production systems. Camel meat is then an ecologically friendly food. Camels also have a very efficient feed conversion rate ( Abdelhadi *et al.* 2017). Nowadays consumers, especially in rich civilizations, tend to favor produces that are environmentally friendly ( Hojnik *et al.* 2019); as a result, this is a very important attribute that needs to be promoted in favor of camel meat.

Camel herds disperse over huge areas instead of clustering together like cattle and sheep. In North Africa (Algeria) for example, the Nomads are sometimes led to adopt a new form of exploitation of the pastures surrounding the oases thanks to the free grazing system (called H’mil). This system is characterized by extensive movements towards the water points of the animals without the control of the camel driver. However, it eats only small amounts at a time and is considered one of the least overgrazing ruminants, unlike sheep and goats. Camel meat can consequently be produced economically compared with other competing meats ( Kadim *et al.* 2014; Abrhaley and Leta 2018). However, free mating on pastures is a common practice, lack of sanitary control; drought and deterioration of vegetation cover are all obstacles to the optimal development of production. Camelids, as *pseudo-ruminant* herbivores, also contribute to greenhouse gas emissions (Methane emissions) in significant proportions on an individual scale due to their ration based on roughage ( Dittmann *et al.* 2014). On the other hand, the concentration of production units around Saharan areas entails the risk of overgrazing and requires the provision of food supplements (concentrated foods), which reduces greenhouse gas emissions, but creates a competition with the man ( Zarrin *et al.* 2020). In other words, the intensification of camel production can have environmental effects comparable to those of pig and cattle farming, but to a lesser degree.

According to FAO (2006) and Gerber *et al.* (2005), the worldwide meat production has been projected to be double by 2050, due mainly to the increase in production and consumption, which is likely to intensify the freshwater crisis in the future ( Chapagain and Hoekstra 2003). The virtual water content (VWC) for camel production has not been well investigated. Quantification of VWC for the camel production plays an important role in understanding the aspects of national water footprint (WFP) in arid regions and is highly needed to guide the allocation of livestock farming and optimize water use ( Papadopoulou *et al.* 2016; Qasemipour and Abbasi 2019). However, the current changes in camel farming practices (the Bedouin system based on camel mobility called H’mil *vs.* semi or intensive systems) based on intensification of the management could modify this conception. According to Chowdhury *et al.* (2017), the unit VWC of camel is 3.5 and 1.7 times the unit VWC for sheep and cow, respectively. One kg of cow and camel meat required 14.6 and 19.7 kg of feed supply, respectively. To my knowledge, there are no works on the carbon footprint and on the water consumption required for the production of 1 kg of camel meat. However, Faye (2013) found that the water consumption for producing one-liter camel milk was multiplied by 9 passing from 938 to 8601 liters of water per liter of produced milk. However, all these results remain to be confirmed by future other studies in different arid and semi-arid regions.

Heavy metals are the main soil contaminants worldwide, which are up taken by crops grown on such contaminated fields which affect both animals and plants ( Ataya *et al.* 2014). *Camelus dromedarius* comprises a major livestock, generating waste bones. The use of a new sustainable approach for the production of nanoparticles from camel bones and their use for the removal of heavy metals from water has been developed ( Alqadami *et al.* 2018).

## The economic potential of camel meat

Generally, in developed countries, meat production has significant impact on nearly all aspects of the envirnonment, including climate change ( Ritchie and Roser 2020). Beef is the most popular and widely produced in the world. Unfortunately, it is also the most inefficient animal meat to produce in terms of the amount of input needed to produce it ( Paris *et al.* 2022). Camel is considered a fundamental pillar of the national economy and food security for many countries in arid regions ( Faye, 2013). Meat professionals see the still virgin camel market as a sure opportunity to do good business and participate in the development of rapidly changing agricultural and agri-food sectors. Camel meat has reduced production costs because camels are usually reared in arid regions ( Kadim *et al.* 2014). In these harsh environments, poor quality feed is the only source for camels and is not utilized by other domesticated species. As a result, camels can produce meat at a lower cost compared to other domestic animals such as cattle, goats and sheep. FAO projects the global meat production to more than double from 229 million ton in 1999 to 465 million ton in 2050 ( FAO, 2006). The average meat consumption worldwide during 2015 was 42.14 kg per capita/year, while in 2019 it was 43.16 kg per capita/year. Thus, the increase in consumption between these years was 2.42%. In the total meat consumption was added beef, sheep, pig, goat, poultry and then other meats (camels, rabbits...). Meanwhile, the global population is also expected to further increase. In combination this will drive up total worldwide meat consumption. Producing more meat to meet the problem of population growth means more animal feed will have to be produced, which also means more land and water will be needed ( Broom, 2019). On the basis of these expected challenges, camel meat is presented as a potential substitute for other red meats because, among other things, camels require fewer resources in terms of land and water ( Watson *et al.* 2016).

Compared to other meats, camel meat is characterized by strengths such as low production costs; be a healthy meat product; a strong consumer base of 1.8 billion in the Muslim world and environmentally friendly production systems ( Mbaga, 2013).

## Camel meat demand in high-value export markets

Recently, more attention has been paid to the nutritional value of camel meat, with the aim of creating additional value for various camel meat products. Although the marketing systems for camel meat are not well organized, there is evidence of a high demand for camel meat. One contributing factor is lack of data and information on marketing of camel products. This causes failure in appreciating the importance of these products in contributing to the development of camel sector. Camel milk, on the other hand, has been marketed and used as a processing aid in several European countries, in particular the Netherlands, Denmark and England. This culminated in 2013 when the European Community (EU, 2013) authorized the import of camel milk from the United Arab Emirates (UAE). This great popularity of camel milk is probably due to the prior knowledge of its nutritional value and human health benefits compared to the more widely consumed cow’s milk ( Konuspayeva *et al.* 2009; Zibaee *et al.* 2015).

According to the Pew Research Center’s Forum on Religion and Public Life (2021), the world’s Muslim population is expected to increase by about 35% in 20 years (2010-2030), by 2030, the global Muslim population is expected to reach 2.2 billion people compared to 1.6 billion in 2010. This huge increase in the Muslim population, coupled with the recent increase in popularity of camel meat in Australia and China ( Wu *et al.* 2011), creates an unprecedented potential for camel meat. For their convenience, consumers are increasingly demanding ready-to-use meat ( Montero *et al.* 2021). This has led to a dramatic development of ready-to-cook/ready-to-eat food product markets. Therefore, these new products have a competitive advantage. Unfortunately, the camel industry suffers from a lack of modernization which results in a lack of access to new meat processing and packaging equipment necessary for the production of denuded cuts.

## The worldwide camel meat supply

The emergence of globalization and like most developing countries around the world, those in arid and semi-arid regions are at the crossroads between many strategic choices regarding food security ( FAO, 2017). Nevertheless, in face of the increasingly competitive global trade, arid and semi-arid countries should undoubtedly improve the quality of local camel meats to take advantage of an opportunity of globalization and to promote these products either internationally or in domestic markets. This can only happen when these countries take into account regional and global regulatory constraints such as sanitary and technical barriers to trade agreements ( WTO, 2021). Camel products in their diversity, including organic and local products currently have no recognized name and no certified origin. These are products that are difficult to recognize and therefore cannot be exported to foreign markets which are demanding in terms of standards and certification of products likely to cross their borders and “appear” on the shelves of their markets. Indeed, the application of Good Manufacturing Practices (GMP) and Hazard Analysis Critical Control Points (HACCP) in the camel sector as mandatory measures must considerably improve the safety and quality of products, and consequently, their access to the European and American markets ( Caswell and Hooker 1996). Controlling the slaughter link in producer countries poses a challenge to the public authorities who have increased the number of equipped slaughterhouses nationwide. They seek to ensure healthy control and flow control to protect the market. Neverless, Service slaughterhouses are very old and lack facilities for the hygienic slaughter and dressing of camels ( Djenane *et al.* 2020). They do not have cold storage, packaging techniques or effluent treatments. This inevitably leads to misuse of animal by-products and poor disposal of slaughterhouse waste, causing environmental pollution.

## Health constraints in camel breeding

Contrary to old ideas, recent data have confirmed the susceptibility of camels to a large number of pathogens ( Hughes and Anderson 2020), and there are currently considered as vectors or reservoirs of transmission of many zoonoses and transhumance animal diseases ( Table 1). As with all production animals, health problems represent a major constraint on the development of animal husbandry. From this point of view, the dromedary has weak points, but differs notably from other animals. The development of dromedary breeding comes up against various zootechnical and health problems, especially since the breeding method does not facilitate veterinary monitoring. The health situation must be examined with attention because it is one of the main obstacles to overcome before being able to expedite meat to developed countries such as Europe.

**Table 1.** The main health problems in camel husbandry worldwide.

Disease	Causative agents	Country	Year	Reference
Camels imported from Sudan and slaughtered at the Cairo slaughterhouse were infected with *Mycoplasma arginini* ( *M. arginini*) causing pneumonia. All isolates were 100% susceptible to florfenicol and streptomycin and 100% resistant to ciprofloxacin.	*Mycoplasma arginini*	Egypt	2020	( Abdelazeem *et al.* 2020)
This infestation is often associated with irritation and traumatic injuries to the skin and with rough hair coat, anaemia, reduced milk production, growth problem and calf mortality.	Ticks ( *Hyalomma dromedarii*) and tick-borne pathogens ( *Anaplasma phagocytophilum*)	Saudi Arabia	2020	( Alanazi *et al.* 2020)
Camels with Rift Valley fever phlebovirus-linked to the torrential rains caused by the effects of the El Niño climatic phenomenon are resistant and the infection remains clinically asymptomatic and misdiagnosed. Disease severity ranged from high rate of abortion, mortality among young animals and fall in production. This mosquito-borne zoonosis is often associated with frequent abortions and sometimes transmitted to humans.	Rift Valley fever Phlebovirus (mosquito-borne zoonosis)	Tunisia, Nigeria	2020 2021	( Selmi *et al.* 2020; Musa *et al.* 2021)
*Coxiella burnetii* is often associated with Q fever, reduction of milk and meat production and infertility. The highest seroprevalence was in female (♀) camels with a previous history of abortion. *This agent* is also associated with its possible long-term persistence in camel hump adipocytes, and consequently represents a threat for herds and breeding farms.	*Coxiella burnetii*	Tunisia	2018	( Selmi *et al.* 2018)
*Coxiella burnetii* is the causative agent of Q fever. Ticks and tick-borne pathogens are major constraints to camel health and production and constitute a public health threat to pastoralist communities.	Ixodidae ticks ( *Coxiella burnetii*)	Egypt	2020	( Ghoneim *et al.* 2020)
*Chlamydophila abortus* as a potential agent to cause abortion in female *camel*s and reduce reproductive efficiency.	*Chlamydophila abortus*	Algeria	2020	( Benaissa *et al.* 2020)
Camel is important sources of infection with *Toxoplasma gondii* (toxoplasmosis) and representing an important role in public health by ingestion of contaminated and undercooked animal products.	*Toxoplasma gondii*	Algeria	2020	( Mohamed-Cherif *et al.* 2020)
Camels and their ticks may play an important role as a reservoir for *C. burnetii* and can be considered as a significant source of Q fever transmission to other animal species and humans.	*Coxiella burnetii*	Algeria	2020	( Bellabidi *et al.* 2020)
Shiga toxin-producing *E. coli* (STEC) is a bacterium that can cause severe foodborne disease. Dromedary camels may be reservoirs of STEC O156: H25 and represent a health hazard for humans with close contact to camels or to consumers of camel derived foodstuffs, such as raw or undercooked ground meat products and raw milk.	Shiga toxin-producing *Escherichia coli* (STEC)	Kenya	2019	( Baschera *et al.* 2019)
Antibiotic resistance is constantly monitored in food-producing animals around the world. *Extended-spectrum beta-lactamases (*ESBLs) are enzymes that confer resistance to most beta-lactam antibiotics. Camels, which provide meat and milk for human consumption, and which attract tourists to arid regions, should be potential reservoirs of these determinants of resistance.	*Klebsiella pneumoniae* *E. coli*	Tunisia	2019	( Saidani *et al.* 2019)
In many countries, camel is important sources of infection with *Trypanosoma evansi* ( *T. evansi*) causes trypanosomosis called “surra”. In Algeria, El Bayadh province was the most affected province with 12% positives. *T. evansi* as a potential agent to cause anaemia associated with intravascular hemolysis with high mortality when the animal is under stress.	*Trypanosoma evansi* type A (Surra)	Algeria	2019	( Boushaki *et al.* 2019)
Q fever caused by *Coxiella burentii*, an obligate intracellular Gram-negative bacterium. It is usually asymptomatic in animals but causes abortion and reduces reproductive efficiency. Appropriate control measures should be taken to reduce cross transmission of infection.	*Coxilla burnetii* ( *C. burnetii*)	Egypt South America North Africa The Near and Middle East	2020 2021 2020	( Devaux *et al.* 2020; Selim and Ali 2020; Rüfli *et al.* 2021)
The endemic nature of the disease demonstrates a complicated epidemiological situation that requires standard serological tests for the diagnosis of brucellosis in camelids. Raw camel milk and products are commonly contaminated with *Brucella* in endemic regions and can be the main vectors of human brucellosis. Consumption of animal products in nomadic areas has resulted in high number of human brucellosis cases compared to cattle, the prevalence of brucellosis in camels could be sometimes insidious and often underestimated because it shows few clinical signs.	Brucella spp. ( *B. abortus*, *B. suis*, *B. melitensis*)	-Egypt -Somalia -Endemic regions -Iran	2020 2019 2021 2019	( Dadar *et al.* 2019; Alamian *et al.* 2019; Khan *et al.* 2020)
The hepatitis E virus (HEV) poses a major health problem due to lack of hygiene in arid areas. The pathogen is mainly transmitted by ingesting contaminated foods. The disease is asymptomatic from the time of infection, but as it manifests, symptoms appear which might include abdominal pain, nausea, jaundice and vomiting. The disease needed more surveillance in arid regions	Hepatitis E Virus	Saudi Arabia	2020	( El-Kafrawy *et al.* 2020)
Camels play a critical role in the development and transmission of MERS-CoV. The possibility of MERS-CoV shedding in the saliva and eye secretions of infected Arabian camels is not ruled out. This explains, at least in part, the transmission mechanism of the Corona virus from animals to humans. Further studies are needed to better understand cross-transmission of MERS-CoV. Coronavirus surveillance and control measures must be regular between camel herds as well as in slaughterhouses. The risk of mutation of the Corona virus can become very contagious for humans and spreads easily from person to person.	Middle East Respiratory Syndrome Coronavirus (MERS-CoV)	Saudi Arabia Jordan	2021 2021	( Hemida *et al.* 2021; Ababneh *et al.* 2021)
Camel trypanosomiasis caused by endemic *T. evansi* poses a major health problem due to the disease in camel species and is responsible for serious economic losses either in milk or meat productions.	*Trypanosoma evansi*	Algeria	2019 2020 2021	( Boushaki *et al.* 2019; Benfodil *et al.* 2020; Boutellis *et al.* 2021)
47.6% of the camels harbored sarcoptic mange infections. The camels showed clear clinical signs of disease caused by mange. Further research is needed for the management of this zoonotic disease among animals.	*Sarcoptes scabiei* var cameli	Egypt	2020	( Ahmed *et al.* 2020)
The causative agent of babesiosis is a parasite of the genus Babesia transmitted by Ixodidae. In infected camels, the infection usually causes fever and intravascular haemolysis leading to progressive anemia, haemoglobinuria and jaundice. It sometimes leads to death, which induces significant economic losses in the camel industry. Early diagnosis and early treatment can prevent further spread of the disease among camels.	*Babesia caballi* (zoonotic parasitic disease)	Iran	2022	( Mirahmadi *et al.* 2022)
Human infection is dramatic, especially when infected camels are slaughtered or when their meats are eaten raw or undercooked.	*Yersinia pestis*	Algeria	2020	( Barbieri *et al.* 2020)
Hepatitis E virus is detected in faecal samples from camels which raises concern about its importance to public health, as camel meat and camel milk are sometimes consumed raw or undercooked by the population of arid regions. This information is of crucial importance for the implementation of appropriate control and/or prevention measures. Chronic infection with the camelid hepatitis E virus is also observed in patients receiving liver transplants who regularly consume camel meat and milk.	hepatitis E virus (HEV)	Ethiopia Middle East	2021 2016 2021	( Lee *et al.* 2016; Nishizawa *et al.* 2021; Bari *et al.* 2021)
Very useful information regarding bovine herpesvirus-1 (BHV-1) infection of camels in Algeria was provided. On the one hand, the seroprevalence of the herd of 21.4% and on the other hand, the mixing of animals is a risk factor which contributes to the spread of the infection. In this case, new animals should be tested or quarantined to reduce the risk of spreading the disease. In arid region, often dromedaries kept in mixed herds with sheep and goats and 41.4% of the camels tested were positive. Therefore, and as a measure to prevent and control the spread of BHV-1 infection among animal populations, separate breeding of each animal species is recommended.	Herpesvirus-1 (BHV-1)	Algeria	2021 2018	( Saidi *et al.* 2018; Benaissa *et al.* 2021)

Camels reportedly represent a reservoir for a variety of potentially zoonotic diseases and pathogens, including *Coxiella burnetii* ( Selmi *et al.* 2018; Devaux *et al.* 2020), *Brucella* spp. ( Khan *et al.* 2020), *Escherichia coli* (STEC) ( Baschera *et al.* 2019), Hepatitis E virus ( El-Kafrawy *et al.* 2020), *Trypanosoma evansi*, which has held the attention of international health authorities for several years, and was added in 2008 to the list of diseases affecting international trade by the World Organization for Animal Health (WOAH), hence the importance of developing methods of disease control ( Antoine-Moussiaux and Desmecht 2008). The Middle East Respiratory Syndrome Corona-Virus (MERS-CoV) ( Hemida and Alnaeem 2019; Hemida *et al.* 2021) a zoonotic disease was also first identified (2012) in humans in Kingdome Saudi Arabia ( Pan *et al.* 2021). It is a camel-to-human spread coronavirus that can originate throughout the slaughter chain and contribute to the virus spread ( Bold *et al.* 2021). Mortality in camel populations has been found to be higher in young camels ( Al-Ruwaili *et al.* 2012). Many factors contribute to this problem, one of which is neonatal diarrhea in dromedary calves which is an economically important disease causing great losses among the herd. Often, the infectious diseases in camel herds are treated with antibiotics. The problem of antimicrobial resistance is of great interest because the extent of this problem is very poorly understood in most arid and semi-arid countries ( Bessalah *et al.* 2016; Abdelazeem *et al.* 2020). According to tradition, in some areas camel products are believed to have therapeutic effects and are often eaten raw, which could put this population at possible risk of zoonotic infection ( Zhu *et al.* 2019). Premraj *et al.* (2020) describe for the first time the interferons in camels (cytokines) playing an important role in the host’s innate immune response against viruses and could be used to better understand the immune system of camels against viral infections.

## Consumption of camel meat and health risks

In one hand, often camels change owners several times during their life before being slaughtered; but the last sale before slaughter usually takes place on the popular market. Illegal slaughter is often the work of occasional butchers who offer uninspected meats for sale. On the other hand, traditional fresh meat products can be contaminated with microorganisms of health and spoilage significance ( Osaili *et al.* 2020a, b). Camel meat safety is of primary importance to both producers and consumers. Therefore, hygienic quality of meat is very important for public health as consumption of poor quality meat may cause several infections and illnesses ( Habib *et al.* 2021). Arid and semi-arid regions have a rich tradition in camel products. They are often prepared in poor sanitary conditions and marketed through informal channels without any official control regarding their health regulatory compliance ( Cook *et al.* 2017; Waldman *et al.* 2020). During slaughter conditions in these countries, the camel meat could potentially be contaminated if there is a rupture of the gastrointestinal tract during carcass dressing and storage is practiced in poor refrigeration. Whereas, in most cases, the retail cutting of raw materials is directly exposed to environmental pollution and thus to increased bacterial contamination ( Djenane *et al.* 2020). The *ante*- *mortem* handling of the animal at the slaughterhouse differs from country to other. In some countries, the animal is stunned before bleeding ( Abdullah *et al.* 2019). In others, stunning is not a common practice. Traditionally, camel slaughter takes place on the ground, with the skin spread under the animal in a prone position role of protection. The risks associated with the consumption of these products have never been estimated on a scientific basis due to the lack of reliable data on the appropriate consumption and epidemiological patterns. The scarcity of scientific studies on the impact of different hazards (biological and chemical) in camel-derived products makes it difficult to conduct scientifically sound profiling or risk assessment studies. Recently, camel meat has received increasing attention as it has caused unrecognized food-borne diseases in developing countries ( Zhu *et al.* 2019).

### Bacteria, yeasts and molds

Osaili *et al.* (2020a, b) observed that during storage, the populations of total flora, lactic acid bacteria, yeasts and molds, *Pseudomonas* spp., as well as *Enterobacteriaceae* increased significantly in stored camel meat. The epidemiology of the newly emerged resistant strains of *Staphylococcus aureus* (vancomycin-resistant) was detected in both abattoir workers (55%) and in camel meat (14.5%) ( Al-Amery *et al.* 2019), which reflects both a public health threat and a food safety concern. Similarly, Methicillin-Resistant *Staphylococcus aureus* (MRSA) was also isolated from camel meat ( Raji *et al.* 2016).

Djenane *et al.* (2019, 2020) showed that the load of psychrotrophic bacteria was kept very far from the critical microbiological threshold (≈ 7 log _10_ cfu/g) in both raw Halal minced beef and camel meat during display. The Microbiological safety against inoculated *Salmonella enterica* ser. Enteritidis and Shiga toxin-producing *Escherichia coli* O157: H7 was explained by the inhibitory effect of the Algerian oleaster, locally called A’hachad ( *Olea europaea* subsp. europaea var. sylvestris) and by combined nisin and *Olea europaea* subsp. laperrinei leaf extract, respectively used as natural biopreservative agents. The same authors showed that after 30 day of storage, *Pseudomonas* spp. count was significantly lower in camel steaks treated with a combination of laper. OLE and nisin suggesting that *Pseudomonas* spp. is the main index for microbiological shelf-life estimation of the product during storage. Similar to these findings, Osaili *et al.* (2021) found that camel meat stored at abusive refrigeration temperatures (10 °C) provides a more favorable environment for the proliferation of spoilage and pathogenic microorganisms, while storage at 4 °C retards microbial development. Thomas *et al.* (2020) studied the prevalence of *Salmonella* in African feed animals and meat by a systematic and meta-analysis review and found that *Salmonella* was detected in 9.3% of 2988 camel samples. Similarly, the prevalence of *Campylobacter* sp. in Iran isolated from camel meat was detected in 0.9% (Rahimi *et al.* 2010). Against all odds, Mohammadpour *et al.* (2020) revealed that camel meat was less contaminated compared to beef, chicken and mutton and free from pathogenic bacteria. Moreover, in a study on the incidence of *Listeria monocytogenes*, Yehia *et al.* (2020) observed that in camel meat from local retail supermarkets located in Riyadh, Saudi Arabia, *Listeria monocytogenes* easily proliferated; such a discrepancy could be explained by the sampling procedure and the limited number of samples studied. Nossair *et al.* (2016) isolated *Escherichia coli* from 44% of camel samples and among the latter 4% were found to be *Escherichia coli* O157: H7, while about 16% of samples were contaminated with *Salmonella* spp. Another study conducted by Al-Sharary ( Al-Sharary, 2014) also reported that 40% of camel meat were contaminated with *Escherichia coli* and *Salmonella* Typhimirium (2%) from different abattoirs in Egypt. Several other studies have been carried out in Iran by Momtaz *et al.* (2013) on camel meat showed that the prevalence of *Escherichia coli* producing Shiga toxin was 13%. While, Hajian *et al.* (2011) and Rahimi *et al.* (2012) reported that in camel meat, the isolation of *Escherichia coli* O157: H7 were only 1.3% and 2%, respectively. Osaili *et al.* (2020a) found that maintaining the refrigeration temperature ≤4 °C during all period of storage is crucial to minimize the growth of spoilage flora, increase shelf-life and decrease the risk of *Escherichia coli* O157: H7 and *Salmonella* spp. in minced camel meat. Fat can be increased thermal resistance of microorganisms ( Osaili *et al.* 2020b). Thus, the low fat content of camel meat products may have acted as a conductor, accelerating the penetration of heat through the meat and thus have very good thermal efficiency against microbes. However, the enthusiasm toward camel meat could have negative repercussions if good practices are not followed, as fresh meat has always been considered a favorable medium for the growth of various pathogenic microorganisms.

### Mycotoxins

The risk of contamination by mycotoxins is certainly underestimated in camel production. Due their ubiquity, the presence of molds in meat and meat products is well documented, and under certain conditions, they may grow and produce mycotoxins, which raise concerns about the consumer’s safety ( Pleadin *et al.* 2021; Kępińska-Pacelik and Biel 2021a, b). Mycotoxins can reach meat and meat products in two ways ( Pleadin *et al.* 2021): Through consumption of camel products from camels which have consumed mycotoxins contaminated feed. Also, mycotoxins ingested by animals can be accumulated in their different organs and tissues ( Tolosat *et al.* 2021), which may be very dangerous for consumers, since mycotoxins cannot be destroyed during the various technological treatments ( Suman 2021), thus, effective controls must be put in place to prevent its development in feed; or through the growth toxigenic molds on the surface of camel products if the storage conditions are not adequate (dry or intermediate-moisture of traditional camel products). Generally, to improve the quality of products made from fresh meats, the meat industry also still makes use of various types of ingredients (natural spices) for making processed meat products ( Montanha *et al.* 2018; Owusu-Ansah *et al.* 2022). The consumption of these probably contaminated final products may pose a risk to the consumer and some studied samples were heavily contaminated with aflatoxins at levels higher than the limits prescribed by European Union (EU) regulations (4 μg/kg) (EU, 2010). The implications of aflatoxins go beyond the public health aspect but also commercial and economic ramifications ( Belasli *et al.* 2023; Ben Miri *et al.* 2023). Mitchell *et al.* (2016) estimate that aflatoxin contamination could result in losses to the cereal industry up to US$1.68 billion per year in the United States (U.S.) due to climate change. Camel feed from different regions of Saudi Arabia were collected and screened for the presence of fungi and mycotoxins ( Bokhari 2010). Authors concluded that feed samples used for camel feeding were highly contaminated with ochratoxin A (85%), aflatoxin (70%) and zearalenone (45%). Tan *et al.* (2020) studied the levels of indospicine (natural plant toxins; *Indigofera* genus) in camel meat and the potential health risks to the Australian population associated with the consumption of wild camel meat and found a high risk. Based on these results, these authors advised to implement a risk management program for indospicine residues to ensure the food safety of Australian camel meat. Indospicin has excellent bioavailability during digestion of contaminated cooked camel meat ( Sultan *et al.* 2018; Tan *et al.* 2020). The consumption of undercooked meat is considered a risk factor for another infection.

### Organochlorine pesticides and heterocyclic amines

Many developing countries still face significant challenges related to the use of organochlorine in agriculture and animal husbandry, which consequently leads to health problems. Camels are, however, mostly grown in arid and semi arid regions where the use of pesticides is scarce and the bioaccumulation of pesticides in camel meat is low and particulary of interest ( Sallam and Morshedy 2008). However, camel meat is often displayed for sale on major highways; extra care should be taken to ensure that the meat is sufficiently cooked and also the taste should not be compromised by the use of high temperatures cooking (especially charcoal grilling) which in turn can lead to the formation of carcinogenic heterocyclic amines ( Li *et al.* 2020). It has been estimated that the relative risk of disease could be multiplied by the hundreds due to premature browning of the hamburger during cooking operations ( Boqvist *et al.* 2015; Lahou *et al.* 2015).

### Heavy toxic elements

Monitoring the trace and heavy toxic elements levels in camel products should pay special attention to toxic compounds in offal, as these are consumed by low-income people as a source of animal protein. Camels are less efficient than other ruminants at detoxifying themselves from these elements in their body ( Al-Qarawi and Ali 2003). Animal nutrition and breeding management play an important role in determining the level of these elements in camel blood and meat ( Faye *et al.* 2008).

## Medicinal value of camel meat

The long-standing practice of using camel products for medicinal purposes was without scientific rationale for centuries. Camel meat has been used since the late sixteenth century in traditional Chinese medicine. The cis-configuration of MUFA in camel meat plays a protective role against coronary heart disease by lowering cholesterol levels and does not reduce high density lipoproteins (HDL). These lipoproteins are responsible for transporting cholesterol to the liver where it can be eliminated. This function makes it possible to prevent the accumulation of cholesterol in the blood vessels and therefore to avoid the risk of atherosclerosis ( Schoeneck and Iggman 2021). Over the past decade, significant attention has been focused on conjugated linoleic acid (CLA) due to its health benefits. Scientists found that CLA appeared to reduce tumor growth in several types of cancer and against atherosclerosis ( Koba and Yanagita 2014; Kim *et al.* 2016). Also, camelid’s meat is characterized by a considerable content of CLA ( Popova *et al.* 2021). For this reason, the main objective of new animal feeding strategies is to increase the concentration of PUFAs and the levels of CLA in forages (especially those of Omega-3 (n-3) FAs). From a nutritional standpoint, the meat of ruminants suffers from an image of a fatty product, rich in SFAs. ω _3_ FAs hold an important place. They include α-linolenic acid (C18: 3 n-3), which is essential because the body cannot synthesize it, as well as its longer chain derivatives such as docosahexaenoic acid (DHA) and eicosapentaenoic acid (EPA). This family of n-3 PUFAs is recognized as beneficial for human health, among other things because of its preventive properties against cardiovascular diseases. In addition, DHA plays a positive role in the inhibition of the carcinoma cells in human colon by decreasing tumor cell growth ( Fluckiger *et al.* 2016). EPA and DHA were detected in camel muscles with higher proportions than those reported in cattle muscles ( Abdelhadi *et al.* 2017). Importantly, the n-6/n-3 ratio in camel meat was lower (≈3) ( Maqsood *et al.* 2015a; Abdelhadi *et al.* 2017) than the recommended values of human health diets: ≤4.0 by the British Department of Health (1994) and lower than that of concentrate-fed bovines (>7) ( Scollana *et al.* 2014).

**Figure 2.**
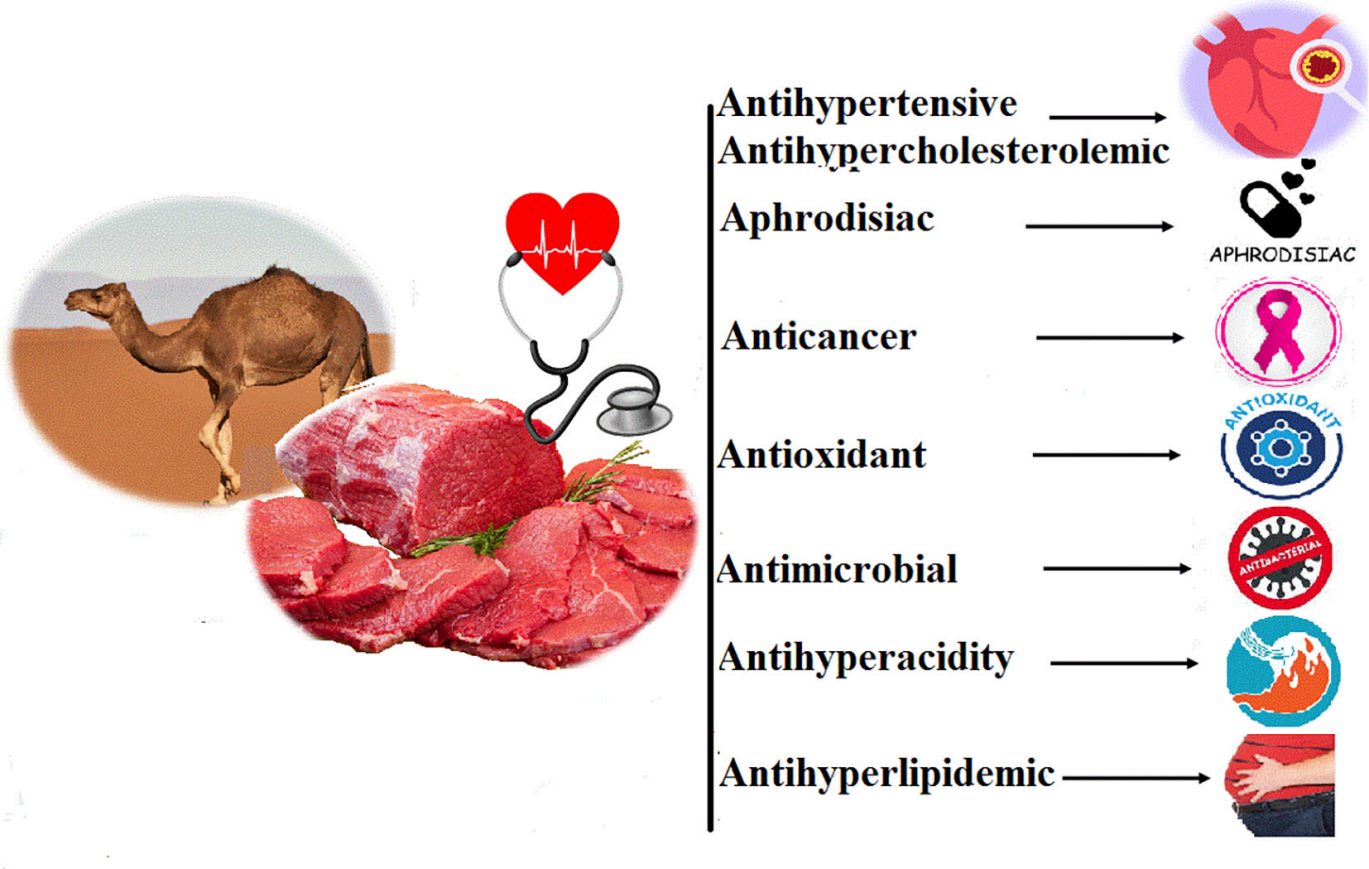
Scheme of the potential medicinal value of camel meat.

Kurtu (2004) and Ayyash *et al.* (2019) claim that camel meat is traditionally used as a remedy for some diseases such as pneumonia, hypertension, hyperacidity, and respiratory disease as well as an aphrodisiac ( Figure 2). However, each human is a different individual with individual genetic, epigenetic, and environmental influences upon their health. Recently, an *in-vitro* comparative study, investigated the health-promoting benefits (anticancer) of semi-dry fermented camel sausages fortified by two novel probiotic *Lb. plantarum* compared with fermented beef sausage ( Ayyash *et al.* 2019). Fermented camel sausages showed greater anticancer activity than beef sausages. Among the traditional therapies used in the Saharan regions is the dromedary fat hump which has been used in molten form alone or mixed with medicinal aromatic plants in the case of rheumatoid arthritis, anti-inflammatory, asthma and eczema. Care must be taken regarding the significance of the results, but in any case, all comparative studies have confirmed the advantage of camel meat on this point. Ayyash *et al.* (2019) found that the antihypertensive via inhibition of angiotensin-converting enzyme (ACE), greater cytotoxicity capacities and antioxidant activities in fermented camel sausages were more pronounced than in beef sausages.

Knowledge about camels was traditionally restricted to limited geographical areas, particularly the Middle East, Asia, and Africa, but the use of camel’s products as a nutrient and for its health benefits is currently known worldwide. However, during the 20 ^th^ century when the intercultural migration of people became very important, knowledge about camels and their products began to reach countries beyond the Middle East, Asia and Africa. However, based on the existing information about camel meat as a functional food, the later, in a similar way, could serve as a source of nutrients and of bioactive agents with therapeutic properties. Scientific efforts continue around the world to identify more therapeutic constituents. Thus, the camel can be “a real gold mine” for researchers to explore. Additionally, it is important to add that reports of the therapeutic benefits associated with non-fatty foods consumption add credibility to the idea that camel meat would have similar benefits. Paradoxically, the role of meat in human nutrition has been widely discussed in recent times. Some scientists have associated the consumption of red and processed meat with the occurrence of common diseases in humans (type 2 diabetes, certain types of cancer) ( Demeyer *et al.* 2016; Libera *et al.* 2021).

## Bioactive Compounds in Camel Meat

In recent times, there has been an increased demand for so-called “organic meats” ( Kamihiro *et al.* 2015). The main reason for this increase is the perception of consumers that organic farmed products generally contain higher concentrations of nutritionally desirable compounds, which makes them “healthier”. Developed countries have adopted the “One Health” approach as the future of animal production ( Silbergeld 2019). Indeed, animal welfare associated with sustainable development is a tool for ensuring the quality of animal products. The breeding techniques (animals space availability and different diets) combined with prolonged maturation system during storage could enhance biomolecules levels in stored obtained red meats ( Salva *et al.* 2009). Therefore, meat composition is an important indicator of its biofunctionality. Mejri *et al.* (2017a) found that in a fermented meat product made from camel meat, several bioactive molecules were identified during ripening. The results showed that identified peptides below 3 kDa have an antioxidant and antihypertensive effect. Gheisari and Motamedi (2010) studied the antioxidant enzymes stability in refrigerated camel meat and confirmed the catalase stability in refrigerated camel meat. However, glutathione peroxidase (GSH-Px) activity decreased in camel meat during refrigerated storage. Several bioactive compounds have been investigated in meats ( Pogorzelska-Nowicka *et al.* 2018; Karwowska *et al.* 2021; Smaoui *et al.* 2021) that are nutritionally and qualitatively important and can potentially be useful in marketing meat products. L-carnitine (β-hydroxy-γ-trimethyl amino butyric acid) plays a crucial biological role. As an antioxidant, carnitine fights off-harmful free radicals, which cause severe damage to cells, but the evidence is still conflicting, and more researches are needed. The L-carnitine concentrations in meat of various species have been reported as 0.69-11.36 μmol/g fresh weigh ( Shimada *et al.* 2004) and the animals which higher concentration had the highest amounts of Mb as an index to the redness of the meat. Similarly, a study evaluated the concentrations of free carnitine, acylcarnitine and total carnitine from camel meat ( Alhomida *et al.* 1995). This assessment indicated 5.17, 2.60 and 7.77 μmol/g fresh weight of free carnitine, acylcarnitine and total carnitine, respectively in camel meat. Similarly, Kadim and Sahi (2018) indicated that camel meat could be a significant source of taurine and carnitine. Taurine has many fundamental biological roles in meats, such as antioxidant ( Selmi *et al.* 2020; Musa *et al.* 2021). However, a higher proportion of acyl carnitine in plasma and skeletal muscle of the camel than in other animal species suggests an adaptive mechanism that could be common to camelids, which may provide energy to various tissues during scarcity of water and food for long periods.

Consumption of red meat provides a small amount of carnitine. It is estimated that a normal diet provides between 20 and 200 mg per day. But it is taking a nutritional supplement of at least 1 g of carnitine that has shown positive effects on the health of people who have already suffered from a myocardial infarction ( Lee *et al.* 2014, 2015). A meta-analysis of 13 clinical studies conducted until 2007 for a total of 3629 patients has shown that carnitine supplementation in patients who have had a first myocardial infarction reduces overall mortality by 27% (due to a cardiovascular accident). More specifically, carnitine is associated with a 65% decrease in ventricular arrhythmias and 40% in heart attacks ( DiNicolantonio *et al.* 2013).

An important dipeptides such as Carnosine (β-alanyl-L-histidine) and its derivative anserine (β-alanyl-L-methyl-L-histidine) are found in high concentration in the muscle of mammalian species. Dromedary camel meat contains 164.9 mg carnosine/100 g and 236.9 mg anserine/100 g fresh weight ( Kadim and Sahi 2018). Carnosine has been proven to act as antioxidant in various meat systems ( Sánchez-Escalante *et al.* 2001; Djenane *et al.* 2004) and also as anticancer actions in various models systems by the restoration of normal cellular homeostasis ( Turner *et al.* 2021). Variations in carnosine and anserine have been found between camel muscles. The average levels of carnosine and anserine in camel muscles has 182 mg and 269 mg/100 g fresh weight, respectively ( Dunnett *et al.* 1997). Little has been known about antioxidant enzymes in camel meat. Chafik *et al.* (2020) suggested a new carbonic anhydrase enzyme that was purified and differentiated from camel liver for atmospheric CO _2_ sequestration. Other endogenous antioxidants enzymes were found in camel muscles such as superoxide dismutase (SOD), coenzyme Q10, catalase and glutathione peroxidase (GSH-Px) (selenium-containing enzyme) contribute to oxidative defense and stimulates the reduction of harmful free radicals ( Gheisari and Motamedi 2010).

## Organoleptic properties of camel meat

In addition to its nutrient value, camel meat also depends on its sensory and organoleptic properties, which are an important criterion for consumers to continue to purchase this meat. It must be said that in addition to the reasonable price, consumers can be benefit from the subtle taste of camel meat, flavored with aromatic and medicinal herbs from the arid regions. Generally, the camel meat marketed in large cities which passes through controlled circuits can only come from aged camels whose meat is mediocre ( Kadim *et al.* 2006a). Thus, city dwellers memorize dromedary meat, tough, unattractive and with inferior qualities in comparison with beef and that of small ruminants, hence the unfavorable prejudices. Thus, to valorize the image of camel meat, and the search for new outlets for this meat, it must promote these organoleptic qualities as other red meats ( Halagarda and Wójciak 2022), but also, strengthen its positive therapeutic image with additional nutritional benefits. Fans of this meat prefer that of small, barely weaned dromedaries, that is to say aged between 6 and 18 months. Camelids meat quality traits are mostly influenced by animal nutrition, animal age and processing methods ( Popova *et al.* 2021).

The ultimate pH (pHu) of camel muscles (5.5 to 6.6) is a consequence of lactic acid accumulation via glycolysis and is considered one of the main factors determining the organoleptic characteristics of camel meat - like drip loss, color, flavor, tenderness, and water-holding capacity, all of which influences consumer acceptance of product - It is related to *post-mortem* biochemical changes during the transformation of muscle into meat ( Abdelhadi *et al.* 2012; Suliman *et al.* 2019; Kadim *et al.* 2013) and is influenced by many factors including muscle physiology, pre-slaughter handling and *post-mortem* treatments. A high pH _u_ is a consequence of low muscle glycogen as a result of pre-slaughter stress ( Loudon *et al.* 2019; Chauhan *et al.* 2019). Soltanizadeh *et al.* (2008) found that the pH decline was faster in beef meat than camel. A more rapid pH decline may inactivate protease activity, meaning a reduction in the proteolysis and subsequent *post-mortem* tenderization. Therefore, there is a possibility that the higher myofibril fragmentation index (MFI) observed in camel meat was due to its higher *post-mortem* pH values, and consequently for higher protease activity ( Kadim *et al.* 2006a).

Storage time is one of the key factors in improving the tenderness of meat. The effect of aging on the organoleptic quality of meat for different species has already been studied by several authors, taking into consideration the periods and conditions of storage. During prolonged storage, the tenderness of the meat improves thanks to changes in the myofibrillar structure due to the activity of endogenous proteolytic enzymes ( Djenane *et al.* 2016). Suliman *et al.* (2019) reported that the improvement in camel meat quality attributes were achieved by 7 days’ storage. Meat quality attributes are influenced by processing treatments such as enzymatic treatment, electrical stimulation, age and method of carcass suspension such as tender stretching *vs.* Achilles hung ( Popova *et al.* 2021). Several authors have shown that the season, the rest of camelid’s in accordance with standards and directives in terms of transport and accommodation, technological processing factors influence the tenderness of the meat obtained from these animals ( Biffin *et al.* 2020a). These authors were able to observe that during the direct shipment of camelids to the slaughterhouse exhibited lower shear-force values than those of animals which had remained at rest for 7 days after transport.

### Expressed Juice

Expressed juice reflects the ability of meat to retain its water content. Generally, consumers prefer juicy over dry meat. The importance of moisture in camel meat is in its marked effects on its processing potential, quality characteristics and shelf-life optimization during storage because of its influence on nutritional value, appearance and palatability.

Meat cuts with low water holding capacity (WHC) are drier and would lose more weight during refrigeration, storage, transportation and marketing. Result of this, loss of minerals, salts and vitamins ( Kadim *et al.* 2013). In meat, juiciness and tenderness are closely correlated. The more is tender the meat the faster the juice is released by chewing and the more juicy the meat appears. Kadim *et al.* (2009a) reported that, the expressed juice in camel meat is higher than in other studies with probably due to age difference between camels and also, higher in than other camelidae such as the alpaca and llama probably because of its lesser fat content ( Cristofanelli *et al.* 2004). Meat with higher pH value has a greater WHC than that with a low pH value; often referred to as: Dark, Firm and Dry (DFD) and Pale, Soft, Exudative (PSE) meats, respectively ( Liu *et al.* 2021).

### Tenderness

Generally, the adult camel remains the most slaughtered category during all periods of the year except the Holy months of Ramadhan and both important Muslim religious feasts when the young camel is in high demand. The female is only slaughtered if she is unproductive or reformed. The effect of age on camel meat quality can be seriously discussed between all stakeholders in the sector in order to optimize the best age for animal slaughtering. The relationships between the contractile and metabolic properties of camel muscle fibers have received little attention. Tenderness is the most important organoleptic characteristic of meat compared to other attribute’s ( Warner *et al.* 2021). Meat tenderness is often determined by muscle characteristics, collagen content and its solubility, *post-mortem* glycogen concentration, proteolytic enzymes content and more likely in the enzyme/inhibitor ratio, a parameter reflecting the efficiency of the proteolytic systems ( Nair *et al.* 2019). Reports that camel meat is less tender than other animals are likely due, at least in part, to the higher average age of slaughtered animals and partly attributed to the fact that camel meat is usually considered a by-product for many years, mainly obtained from old females and males (> 10 years) ( Kadim *et al.* 2008). To remedy this drawback, the conversions of this toughest camel meat to sausages or minced meat eliminate toughness and reduce the required cooking time. Djenane *et al.* (2020) suggested that the tenderness in camel meat is not one of the main quality criteria for consumers in arid regions for several reasons: On the one hand, it is often older animals that are used during the slaughter; on the other hand, people in these regions have a low tendency to consume camel meat “as is”. However, its higher connective tissue content makes it a tougher meat ( Kadim *et al.* 2008). Kadim *et al.* (2006a) recommended the slaughter of male camels between one and three (1–3) years of age to obtain tender meat. A number of studies have also shown that shear–force (opposite to tenderness) values increase with animal age ( Djenane *et al.* 2016). Any differences due to age may be directly related to histological changes in muscle structure and composition and in the nature and quantity of connective tissue. In regard to the tenderness of camel meat, the values reported in the literature, measured by the same method as beef meat, point to higher values of Warner–Bratzler–shear force (WB-SF) than reported for beef meat. Shear–force of camel meat was positively correlated with total collagen content ( Al-Owaimer *et al.* 2014) as it is the case in cattle ( Battaglia *et al.* 2020).

Kadim *et al.* (2006a) suggested that the shear-force value of meat from aged camels (8 years) was 48% higher than that from young camels (1–5 years). The WB-SF values for various camel muscles were determined by Kadim *et al.* (2013). The *Infraspinatus* (IS), *Triceps brachii* (TB), *Longissimus thoracis* (LT) muscles had significantly lower shear–force (6.3–6.7 kg) values than *Semitendinosus* (ST), *Semimembranosus* (SM), and *Biceps femoris* (BF) muscles (9–12.9 kg), which might be due to less connective tissue. Suliman *et al.* (2019) reported a similar result for camel muscles. Interestingly, Babiker and Yousif (1990) found that the total collagen content is greater in camel *Longissimus dorsi* (LD) muscle than other muscles, possibly due to the stabilizing the hump attached morphologically to this muscle. All of these muscles have proven to be the best quality muscles for marketing. Consumer acceptance of identified individual camel muscles should be assessed with the aim of developing positive marketing strategies towards consumer perception of camel meat. However, evidence suggests that the tenderness of camel meat is not significantly different from that of beef if slaughtered at the same ages ( Kadim *et al.* 2011). Many exogenous enzymes have the capacity to indiscriminately degrade muscle proteins, leading to this effect, a significant degradation of the miofibrillary system of the latter and, consequently, the sensory qualities of obtained meat ( Eom *et al.* 2015; Bagheri Kakash *et al.* 2019). The MFI of old camels (6 years) was lower than youngers (< 3 years) ( Kadim *et al.* 2008, 2009a). The authors observed a strong relationship between physical disruptions of the myofibrils and the tenderness. MFI is one of the most widely used methods to determine *post-mortem* proteolysis in meat of various species. This index is a very useful indicator of meat tenderness (shear–force and sensory panel tenderness). Smili *et al.* (2022) reported that MFI of Algerian Sahraoui dromedary meat was significantly affected by both slaughter age and *post*– *mortem* period. The same authors confirmed that the 30–kDa band is a proteolytic product of Troponin–T in camel *Longissimus* lumborum muscles. Soltanizadeh *et al.* (2008) observed the appearance on the 3 ^rd^ day of storage of a 30 kDa band in camel meat which was linked to the higher degree of proteolysis. Therefore, various methods have been developed for tenderizing camel meat. Abdel-Naeem and Mohamed (2016) found that addition of ginger ( *Zingiber officinale*) extract in the formulation of camel meat burger patties resulted in extensive fragmentation of myofibrils; however, papain extract caused noticeable destructive effect on connective tissue. Besides tenderizing properties, antioxidant characteristics of ginger extract have been reported by different workers ( Wen *et al.* 2020). The tenderness of meat can also be improved by the use of exogenous enzymes which break down muscle proteins including connective tissue ( Bhat *et al.* 2018; Gagaoua *et al.* 2021a, b).

However, the action of photolytic enzyme on collagen is limited, which makes it less useful for muscles from old camels with high collagen content. Recently, plant proteases have been used as a sustainable manner to improve the texture of tough camel meat and to develop functional meat products that contain bioactive molecules with antioxidant activities ( Gagaoua *et al.* 2021a). Abdel-Naeem and Mohamed (2016) intended to include tenderizing agents such as ginger extract and papain and their mixture in the formulation of camel meat burger patties to improve the physico-chemical and sensory characteristics of the product. Treatment with tenderizing agents resulted in significant reduction of the shear–force values and increase of the collagen solubility. From another perspective and to overcome the problem of toughness in camel meat, Smith *et al.* (2016) showed that whatever the age of the animal or the sex, electrical stimulation (ES) could drastically decrease the hardness of camelid meat, expressed in WB-SF. ES of carcasses at low voltage (20 to 100 V) is a proven method to prevent the onset of cold contracture ( *cold*– *shortening*) and for improving tenderness of meat (Polidori and Vincenzetti 2017). In excised camel muscles that are cooled while still a pre-rigor condition, *cold*– *shortening* might take place ( Kadim *et al.* 2009b). In the case of camel meat, the effects of *post*– *mortem* ES (90 V) 20 min was assessed by Kadim *et al.* (2006a, 2009b), muscles from young camel were not affected by *cold-shortening* and ES had a significant effect on meat quality attributes. Muscles from electrically non-stimulated carcasses had a significantly higher WB-SF value (9.47 kg/cm ^2^) compared to stimulated carcasses (6.97 kg/cm ^2^) ( Kadim *et al.* 2009b). Also the method of suspending camelid carcasses using a technique called “tenderstretching” favored the tenderness of the meat ( Smith *et al.* 2016). The combination of the two techniques (ES/tenderstretching) produced an additional effect in improving the tenderness of the meat of camelid’s ( Biffin *et al.* 2020b). The key mechanism by which ES improves tenderness is by rapidly decreasing the concentration of adenosine triphosphate (ATP), which reduces the potential for myofibrils to contract and *cold-shortening* if *post-mortem* carcasses are immediately refrigerated.

Research on the effect of prolonged storage to improve the tenderness of camelid meat has shown a marked improvement between 10 and 25 days of ageing ( Smith *et al.* 2016, 2017a). Ageing is one of the *post-mortem* treatments which increase camel meat tenderness. The time required for ageing varies with the type and size of muscles, species, age of the animal, level of endogenous enzymes, contraction status and connective tissue content ( Suliman *et al.* 2019). As in the case of arid countries, moderate temperature storage (> 10 °C) may accelerate the ageing process and, this with all the microbiological risks involved. This is useful knowledge for the meat industry for optimizing processing and storage procedures for camel meats.

### Color

Meat color is one of most important quality characteristics since it determines the consumer acceptability of meat, it is often the only attribute that guides the consumer to purchase, especially in retail display stores ( Djenane *et al.* 2001; Djenane and Roncalés 2018). In the US, an average of 2.55% of total red meat sold is discarded annually due to discoloration (13.4 million kg, the equivalent of $3.73 billion loss) ( Ramanathan *et al.* 2022). Many factors such as muscle fiber composition, anatomical muscle location and physical activity, feeding diets, proportion of intramuscular fat, pH _u_, as well as the technological processes of conversion of muscles to meat have an influence on the degradation of meat color. It is now possible to act on these, in particular thanks to packaging and controlled atmosphere in retail display markets ( Martínez *et al.* 2005). Camel meat is described as “ *raspberry red*” and sometimes brown in older animals (due to a higher concentration of Mb) with a slight sweet taste which would be due to a relative richness in glycogen ( Kadim *et al.* 2013). Abdelhadi *et al.* (2013) reported color differences on camel meats with a seasonal effect associated with varying animal’s nutrition throughout the year. Similarly, Abdelhadi *et al.* (2012) found significant effect of seasons on color camel meat. It was significantly darker red in autumn compared to summer and less yellow in autumn than in summer and winter. In addition to season’s differences, ambient temperatures were reported to affect muscle biochemical characteristics and color. The color characteristics of camel meat can be influenced by the *post-mortem* storage period and the type of muscle ( Suliman *et al.* 2019). The characterization of the total haem pigment, Mb and haemoglobin (Hb) in camel meat and the associated changes during refrigerated storage was studied by Maqsood *et al.* (2015a). Authors noticed a decrease in these compounds content with concomitant decrease in redness (a* value) during the storage. A high redness (a*) color component in the camel meat was associated with a lower lightness (L*), which might be due to an increase in Mb content. Camel muscle lightness L* values indicated that the muscle had the lightest lean color, which was possibly due to high fat content ( Kadim *et al.* 2013). However, meat from older camels are lower L* (darker) and higher a* (redder) than that of younger camels ( Kadim *et al.* 2006a). *Post-mortem* proteolysis increases light scattering properties of meat and thereby influence L*, a* and b* values ( Hughes *et al.* 2020), which is also directly related to the pH _u_. Meat proteins have an isoelectric pH (pH _i_ = 5.5). This results in open muscle structure and as a result lighter scattering between the myofibrils, which makes the surface of the meat lighter. It is obvious that during the retail display, the meat surface color is unstable. This phenomenon is due to several factors such as, *post-mortem* biochemical changes in protein and lipid fractions of the meat, surface microbial overgrowth, oxidation of lipids and Mb and finally, the phenomenon of photo oxidation due to the fluorescent tube installed in the refrigerated display cases ( Djenane *et al.* 2003).

Strategies for preserving color camel meat were the application of *post-mortem* low voltage ES ( Smith *et al.* 2016). Abhijith *et al.* (2020) reported that meat from electrically-stimulated carcasses, have a brighter red color than meat from non-stimulated carcasses. Using an appropriate packaging and storage conditions (vacuum, MAP and active packaging) without the use of any synthetic additives can play a major role in color enhancement and preservation of meat during storage with significant lag of microbial growth, lipid oxidation and *off*– *odor* development ( Ait Ouahioune *et al.* 2022a; Camo *et al.* 2011). Maqsood *et al.* (2015b) and Maqsood and Benjakul (2010) reported that camel meat contains total haem content expressed as haematin was 92.3 mg/g which higher than what was found in beef (76.16 mg/g). However, the presence of a high heme content could contribute to fast meat oxidation during its refrigerated storage. Average Mb contents in camel meat was found to be 7.16 mg/g ( Maqsood *et al.* 2015a). In contrast, Gheisari (2011) reported 3.64 mg/g. This difference could be due to several experimental factors such as part of the carcass, solvents and extraction conditions. In general, the quality of camel meat has been assessed using traditional conventional methods, and it will be important for the camel sector to take into account the new technologies already applied to beef (optical sensors, electromechanical probe, video image analytics, predictive genomic markers).

## Technological aspects of camel meat

Comprehensive data describing the technologically aspects of camel meat are lacking. However, in recent times, several Australian researchers have taken an interest of camelids as a valuable source of meat ( Logan *et al.* 2019; Biffin *et al.* 2019, 2020b). Arid countries will have a good opportunity to exploit camel meat for the production of many meat products ( Djenane *et al.* 2020). Camel meat can be processed in similar ways to beef, producing similar products with similar acceptability. The “industrial” development of camel meat still requires some significant improvements in terms of slaughter conditions, carcass cutting and classification. The study methodologies already developed by the various research groups for other species can be used for the camel species for a better appreciation of its interest in the sector. The various studies carried out on camel meat have focused on evaluating its content in cholesterol, fatty acids, amino acids, vitamins and minerals. The qualitative and technological characteristics of meat are also strongly influenced by husbandry methods which include animal feedings.

### Processing technologies

Due to the climate conditions in arid areas, it is virtually impossible to keep meat or meat products fresh for longer periods of time. In some countries, the meat processing industry has taken an interest in upgrading camel carcasses based on the characteristics of the different muscles ( Kadim and Purchas 2019). In contrast, in other countries, the transformation of camel meat is almost non-existent or by natural drying in the sun ( Boqvist *et al.* 2015). The range of camel meat products is limited, and is characterized mainly by dried meat products, made by crude methods, smoking, brining, curing and marinades. In addition, the different combinations of these treatments are very common at the local artisanal scale in Africa and Asia for many years and to gain maximum acceptability ( Ulmer *et al.* 2004; Farouk and Bekhit 2013). For the fresh camel meat processing, the WHC or drip loss is known to influence its technological quality and is an essential quality parameter for both the industry and the consumer. WHC is affected by degradation of myofibrillar proteins ( Maqsood *et al.* 2018; Djenane *et al.* 2023) and is considered to be a serious quality problem. WHC affects the retention of minerals, vitamins and volume of water ( Apple 2007) and is influenced by muscle pH because of the electrostatic effects of meat proteins. Maqsood *et al.* (2015a) observed a continuous increase in drip loss in camel meat when the storage time increased. Similarly, Abdelhadi *et al.* (2013) also reported that the drip loss in the camel meat continuously increased during storage. Dawood (1995) and Kadim *et al.* (2006a) reported that meat from young camels had higher WHC values than older camels, probably due to variations in fat content and the binding ability of meat proteins. Camels younger than three (3) years had greater pH than camels older than six (6) years. Many studies have examined the possibilities for reformulation of meat products using camel meat due to their nutritional value and their low fat content. Camel meat has been processed into burgers, cured or cooked ham, kebabs, meatballs, patties, mortadella, merguez, sausages, and shawarma to add value ( Kadim *et al.* 2008; Sbihi *et al.* 2013; Kadim and Sahi 2018). Australian processed camel meat has been accepted as international traded meat products. It is now exported to Canada, Europe, China, United States and Saudi Arabia. In recent years, the market and demand for ready-to-eat (RTE) food products has increased due to lifestyle changes. Camel meat-made burgers are currently a growing trend, especially in Arab countries, as a replacement for conventional beef patties. Tasting tests have shown better acceptability of camel-burger compared to the same product prepared with beef ( Kadim *et al.* 2008). In other study, Babiker and Tibin (1986) compared the physical, chemical and palatability aspects of camel sausage and beef and they stated that camel sausage made with 10% and 15% fat was acceptable by the panelists and not significantly different from the beef sausage and concluded that camel meat can successfully replace beef in sausage manufacture. In other terms, the functional properties of camel meat are very similar to those of beef. Therefore, it can be a potential competitor of beef for the production of meat products especially for its low price ( Mbaga, 2013). The reformulation of meat products based on innovation strategies that promote better consumer health and to obtain better nutritional compositions and better product quality is the main tool for any development of the meat sector. The potential of camel meat has received increased attention. In this context, Kargozari *et al.* (2014) compared the fatty-acid and volatile-compound profiles, sensory qualities and the physicochemical of dry sausage (Sucuk) made from camel meat and hump fat with sausages made from beef and beef fat and from a mixture of both. The authors suggest that the physicochemical properties and some textural attributes of camel meat sausages were comparable to those of beef sausages. Nerveless, the flavor components were quantitatively much higher in sausage made with camel meat.

The demand for foods that can improve health status and provide therapeutic benefits is expected to increase as a result of population growth. Sausages fermented with probiotic bacteria are considered a type of functional food that may provide several health benefits ( Ayyash *et al.* 2019; Mejri *et al.* 2017b). In comparative study, Kargozari *et al.* (2014) found that camel fermented sausages exhibited greater resistance to lipid oxidation (lower thiobarbituric acid-reactive substances: TBA-RS) during storage than beef sausages. Authors obtained an n-6/n-3 ratio = 6.22 in sausages made with beef, compared to 2.95 in those made with camel meat. This indicates that camel sausages fit perfectly into the recommendations for this ratio. Abdel-Naeem and Mohamed (2016) found that addition of ginger ( *Zingiber officinale*) and papain (cysteine protease acquired from papaya plant: *Carica papaya*) extracts in the formulation of camel meat burger patties resulted in improvement of the lipid stability and significant increase of sensory scores (juiciness, tenderness and overall acceptability) of treated burger patties during storage. A recent study by Gagaoua *et al.* (2021b) showed that marinade with plant proteases has been used to improve the texture of tough camel meat. This can be a valuable strategy to develop locally tender camel and healthier food products that contain bioactive compounds with antioxidant activities. Maqsood *et al.* (2015b) studied the effect of phenolic compounds especially those of tannic acid and catechin on sensory quality and fatty acid profile of camel meat during storage and found that incorporation of phenolic compounds improved sensory scores for all attributes and found the effect of lipid oxidation on fatty acid profile. The same authors studied the effect of these compounds on the degradation of proteins by the SDSePAGE method. There was a drastic change in different protein bands. On the 9 ^th^ day of storage at refrigeration temperature, myosin heavy chain, actin, α-tropomyosin, β-tropomyosin and troponin T suffered greater degradation in camel meat when compared between fresh camel meat at day 0 and those stored for 9 days. Furthermore, authors suggest that tannic acid and catechin could prevent protein breakdown in chilled ground camel meat, which was most likely due to their antimicrobial and antioxidant activity. Yarmand *et al.* (2013) studied the optimization of the various cooking processes such as (braising, roasting and microwave) on camel meat quality. The scanning electron microscopy (SEM) showed that roasting caused more structural damage than either braising or microwave heating.

The solar drying under the open sky is very widespread in the Saharan areas due to ancestral practices and the lack of adequate access to the electrical means. Further, it should be noted that despite the low cholesterol content of fresh camelid meat, the salt curing process can induce significant amounts of cholesterol in the finished product ( Mamani-Linares and Gallo 2014). To avoid this problem, experiments of drying camel meat without salting were carried out by Chaouch *et al.* (2018).

The growing demand for Halal gelatin and the rejection of gelatin mainly porcine sources have encouraged scientists to search for alternative sources such as camel skin ( Ahmed *et al.* 2020). Recently, Al-Hassan demonstrated that gelatin could be extracted from camel skin as a promising source of Halal gelatin that might be acceptable for Muslims for religious reasons ( Al-Hassan, 2020).

### Packaging technologies

The evolution of modern retail outlets with better packaging, labeling, and cold chain facilities will address the drawbacks of the current situation in the camel meat sector. Referring to previous studies in the packaging technologies, very little data is available on the application of innovative techniques in shelf-life extension and preservation of camel meat ( Table 2). Microbial spoilage coupled with color change and lipid oxidation are the critical factor limiting the shelf-life and consumer acceptability of the camel meat ( Maqsood *et al.* 2015a, b). Haem pigment is known to be potent catalyst of lipid oxidation in muscle foods. With increasing storage time, the damage of the haem pigment results in the release of Fe which can accelerate lipid oxidation ( Maqsood and Benjakul 2010). Recently, processing of modified atmospheres (80% O _2_, 20% CO _2_) camel steaks stored under refrigeration conditions incorporated with nisin and olive leaf extracts ( *Olea europaea* Subsp. laperrinei) have been reported by Djenane *et al.* (2020). Authors reported that redness (a*) values in camel steaks tended to decrease during chilled storage. High oxygen (80%) packing with added natural antioxidants can be used as a strategy to retain the redness of camel meat during retail display. Jouki and Khazaei (2012) also found the lowest degrees of lipid oxidation value for vacuum packaged camel meat compared to MAP (60% CO _2_; 40% N _2_) and air packaged during chilled storage and concluded that vacuum packaging can be used as an effective packaging strategy against deleterious consequences of lipid oxidation. In this same perspective, Maqsood *et al.* (2016a) reported that the shelf-life of chilled vacuum stored camel meat could be extended to 18 days compared to packed and wrapped samples.

**Table 2.** The reports of the last decade (2012-2023) on strategies for enhancing the safety, quality and shelf-life of camel meat.

References	Product and conditions	Results/Conclusions
( Osaili *et al.* 2020a)	Fresh ground camel meat	The use of abusive temperatures to preserve meat is common in arid countries. A study on the growth of pathogenic and spoilage bacteria in fresh ground camel meat was conducted during storage at 4 °C and 10 °C for one week. Meat samples were inoculated with *Escherichia coli* O157: H7 and *Salmonella* and treated in a water bath at 55–65 °C. At 4 °C, the number of *E. coli* O157:H7 and *Salmonella* spp. decreased drastically in the samples. In contrast, samples stored at 10 °C showed significant increases in microbial populations. Regarding the spoilage flora, a significant increase was observed in both cases of storage temperatures. These results can be a tool to help improve the safety and quality of camel meat during storage and to facilitate the validation of the heat treatment of camel meat products.
( Osaili *et al.* 2020b)	Camel meat burgers	Adding camel's hump fat to meat products could result in increased thermal resistance of bacteria in the matrix. The results of this study could help camel meat processors to validate thermal processes.
( Osaili *et al.* 2021)	Packaged Marinated Camel meat	The use of the combined method of vacuum packaging (VP) with EOs and its components (carvacrol, cinnamaldehyde, and thymol) could increase microbial stability against spoilage-causing microorganisms and increase the shelf-life of marinated camel meat during storage at 4 and 10 °C. Increasing the temperature from 4 to 10 °C under aerobic conditions increased the rate of microbial growth in both control and marinated camel meat; however, EOs were more effective at 10 °C. The Incorporation of 2% EO into marinated camel meat stored at abusive temperature (10 °C) under aerobic conditions improved product shelf-life.
( Djenane *et al.* 2020)	Packaged fresh camel steaks	The highly modified oxygen atmosphere (80% O _2_) combined with leaf extract *Olea europaea* Subsp. laperrinei treatment alone, or combined with nisin, can be a promising tool and constitute a relevant strategy to enhancing the shelf-life of packaged camel meat (1 ± 1 °C) by control microbial growth and oxidation phenomena without undesirable effects on its sensory acceptability during 30 days of storage.
( Khezrian and Shahbazi 2018)	Packaged minced camel meat	To increase the shelf-life (microbial, chemical and sensory properties) and against *Listeria monocytogenes* and *Escherichia coli* O157: H7 growth during storage at refrigerated condition a novel films based on nanocomposite incorporated with *Ziziphora clinopodioides* EO alone and in combination with *Ficus carica* extract were investigated as active packaging materials for minced camel's meat. Minced camel meat's shelf-life can be extended by using chitosan film containing natural biomolecules separately and in combination with cellulose nanoparticle. The synthesized designated films are biodegradable, thus potential in modern active food packaging for the concern of food protection and environmental problems.
( Maqsood *et al.* 2015b)	Fresh camel meat	Upon addition of different natural phenolic compounds at a level of 200 ppm, thiobarbituric acid reactive substances (TBA-RS) as well as microbial counts were retarded, especially in samples added with tannic acid and catechin compared to control samples without sensory modification of camel meat on day 6 of storage. Therefore, natural phenolic are also effective for sensory stability of camel meat during refrigerated storage.
( Al-Bachir and Zeinou 2009)	Irradiated Fresh camel meat	Treatment of camel meat with gamma irradiation (2, 4 and 6 kGy) reduced the total bacterial flora in treated meat. Thus, the microbiological shelf-life of camel meat was significantly extended from less than 2 weeks (control) to more than 6 weeks (treated samples). No significant differences in thiobarbituric acid (TBA) values, volatile basic nitrogen (VBN) and sensory properties of camel meat were observed due to irradiation.
( Jouki and Khazaei 2012)	Packaged fresh camel meat	By using an anoxic modified atmosphere packaging with higher carbon dioxide (60% CO _2_ + 40% N _2_) and combined with refrigeration (4 °C) can be used as an effective tool to promote the physico-chemical attributes of fresh camel meat in arid countries without any noticeable sensory changes during three weeks of storage.
( Yehia *et al.* 2021)	Fresh camel meat	The combination of natural biopreservatives (Citrox + chitosan) constitutes a promising tool and a relevant strategy for the control of *Campylobacter jejuni* and also for preserving the quality of camel meat packed under vacuum at 4 and 10 °C for 1 month.
( Shahbazi *et al.* 2018)	Raw minced camel meat	*Mentha spicata* EO was incorporated at different concentrations (0.5 to 1.5% v/w) into raw minced camel meat. The final microbial population decreased 1 to 4 log cfu/g in treated samples compared to control. *M. spicata* EO as a natural substance could successfully extend the shelf-life and control of *Listeria monocytogenes* in fresh minced camel meat stored at refrigerated temperature for 12 days of storage. Color, odor and also appearance of the treated samples were better than control samples. *M. spicata* EO can be used as a replacement to synthetic preservatives as well as synthetic flavorings in minced camel meat.
( Ansarian *et al.* 2022)	Packaged minced camel meat	Minced camel meat was packaged in nanoemulsion-based film with EO during 3 weeks’ storage at 4 °C. Furthermore, meat wrapped with nanoemulsion-based film containing EOs showed better oxidative stability and shelf-life results compared with the control group.
( Zhao *et al.* 2020; Hai *et al.* 2020)	Fraud and camel meat	Authentication of meat products is important to ensure fair competition, consumer benefit, and food safety. The difference in price between camel and other species may be an incentive to adulterate meat products. With an increased demand for camel meat, camel meat-related food products are susceptible to food fraud. These food frauds can be detected in a very specific way and relatively quantified thanks to triplex real-time polymerase chain reaction PCR assay or real-time PCR-lateral flow immunoassay (LFI) (making it possible for example to determine the percentage of each animal species in relation to the total amount of meat). Overall, this new method could be ideal for government laboratories to detect food fraud of this kind. The designed triplex real-time PCR assay was shown to be a specific, sensitive, and efficient technique for the identification of camel and other species DNA in foodstuffs.
( Husain *et al.* 2021)	Fresh camel meat	Biofilm formation by drug-resistant pathogens poses a major threat to food safety and public health. Camel meat can harbor biofilms formed by the genus *Pseudomonas* spp. producing metallo-β-lactamase (MβL) enzymes. The effect of the flavone naringin on biofilm formation produced by *Pseudomonas* spp. has been determined. Naringin significantly reduced biofilm formation (57% of reduction). Thus, it is envisaged that naringin can be used as a natural food preservative against the formation of biofilms in the meat industry.
( Chaouch *et al.* 2018)	Dehydrated camel meat	Experiments were carried out for drying camel meat without salting under desert conditions. Controlling camel meat drying conditions aims to maintain nutritional quality, especially protein, which is very sensitive to temperature and salt. This provides the consumer with a guarantee of a dry product that meets hygiene standards and high nutritional quality. The development of solar drying technology and capacity of solar energy storage technology is made possible in the Saharan regions. This ancestral technology will allow the creation of a camel meat drying industry and promote camel breeding by installing this new mode of consumption, neglected for a long time.
( Khan *et al.* 2021)	Marinated camel meat	Toxic products from the Maillard reaction that form from the reaction of sugars, amino acids and creatine/creatinine when cooking protein rich food poses a major threat to food safety and public health. The effect of marinades was tested in camel meat at different times (1, 6, 12, 24 h) during refrigeration (4 °C) before frying. The marinades caused an overall reduction of Heterocyclic amines (HCAs) and the decrease was more noticeable with long marination time ≥ 6h. The reduction of HCAs, after 24h marinades was 40–100%. Therefore, longer marinating times are recommended.
( Abdel-Naeem and Mohamed 2016)	Tough camel meat	The action of photolytic enzyme on collagen is limited, which makes it less useful for muscles from old camels with high collagen content. Plant proteases have been used as a sustainable manner to improve the texture of tough camel meat and to develop functional meat products that contain bioactive molecules with antioxidant activities. Attempts to include tenderizing agents such as ginger extract and papain and their mixture in the formulation of camel meat patties to improve the physico-chemical and sensory characteristics of the product have been carried out. Treatment with tenderizing agents resulted in a significant reduction in shear-force values and an increase in collagen solubility.
( Maqsood *et al.* 2016a)	Packaged camel meat	The Impact of different packaging conditions on various quality attributes of camel meat during 18 days of refrigerated storage was investigated. Camel meat packed under vacuum displayed lower thiobarbituric acid reactive substances (TBA-RS) associated with *off-odor*, lower counts for different microorganisms, higher Redness (a*) values and major scores on odor, color and overall acceptability compared to controls (air and wrapped). Therefore, vacuum packaging was very effective in retarding lipid oxidation, microbial growth and protein degradation, as well as maintaining the sensory quality for the fresh camel meat.
( Bahwan *et al.* 2023)	Cooking camel meat	The impact of four different cooking techniques viz: boiling, grilling, microwave, and frying; on the physicochemical characteristics of camel meat was investigated. Boiled camel meat had lower hardness values compared to the other treated samples. Consequently, boiling was the more suitable cooking technique for producing camel meat with a reduced hardness value and lower lipid oxidation level.

Packaging is often seen as an unwanted ingredient associated with food, yet it is an essential and inseparable ally. However, the topic is very serious because the packaging, without departing from its irreplaceable role in food preservation, is accused today of polluting food and polluting the environment. The world’s population continues to grow and so do natural resources. Consumers not only care more and more about what they eat, they are also more concerned with the packaging of the food they buy. This increased focus on environmentally friendly consumption leads to the development of more sustainable business practices and a low-cost circular economy ( Baghi *et al.* 2022). This aims to conserve materials and resources as long as possible. Because of the inability of plastics to degrade, the environmental pollution it causes is a major global concern ( Chamas *et al.* 2020). To overcome this serious problem of plastic waste, the modern food industry requires new and effective approaches to preserving perishable food products. The incorporation of bioactive molecules (antimicrobial and antioxidant) in packaging materials can remarkably help extend the shelf-life of perishable foods by retarding microbial growth and reducing oxidation ( Chaouch *et al.* 2018; Ait Ouahioune *et al.* 2022a, b; Camo *et al.* 2011; Lammi *et al.* 2018a, b). Nanotechnology can play a fundamental role in the meat industry, from processing, preservation to packaging of fresh or processed meat products. Nanoparticles have gained momentum as a smart tool in several fields such as medicine, pharmacy and agri-food ( Singh *et al.* 2016; Lamri *et al.* 2021).

### Preservation and extended shelf-life

**Figure 3.**
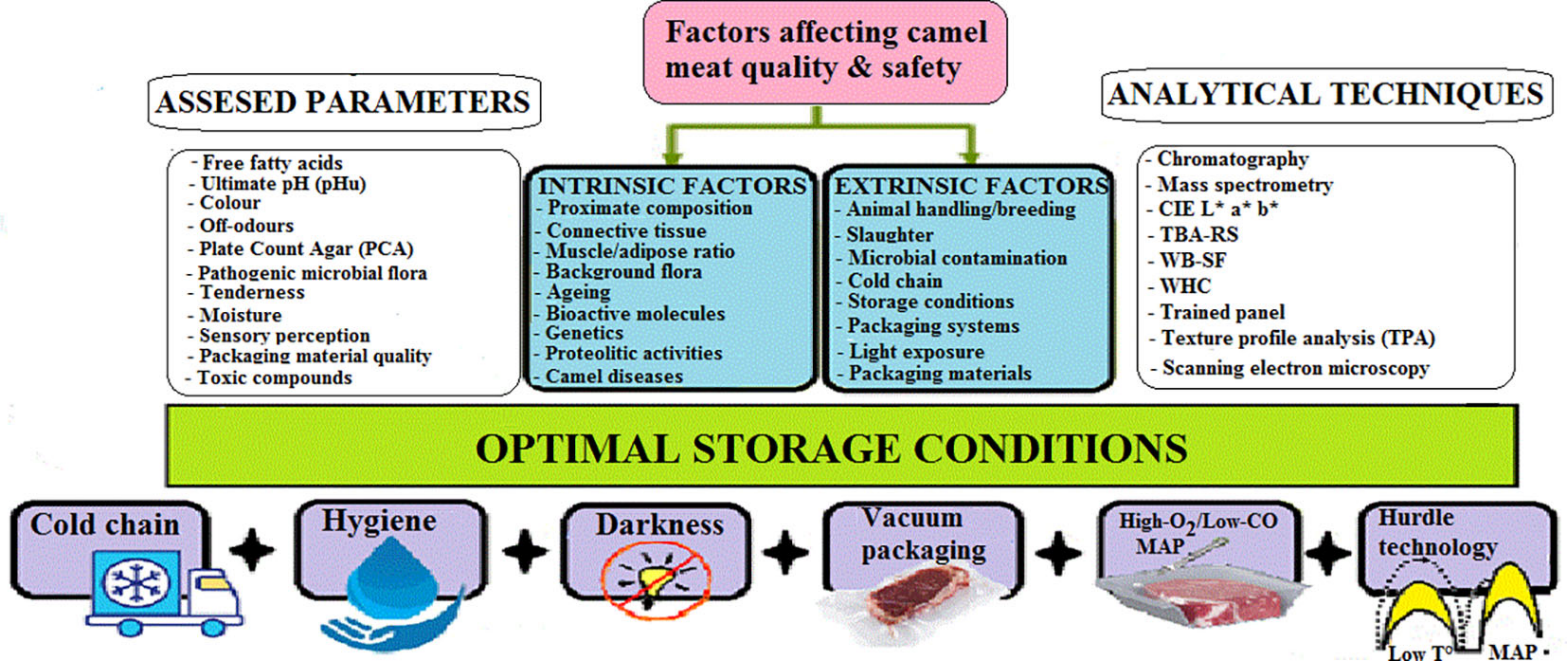
Factors affecting camel meat quality and safety and its preservation.

Fresh meat properties are important in determining shelf-life include, lipid, pigment and microbial stability, and finally its acceptability ( Djenane *et al.* 2023). Deterioration of meat quality during storage, may include discoloration, *off-flavor* and *off-odor* development, nutrient loss, texture changes, and possibly become unsafe for consumers. Over the past decade, fresh meat commercialization strategies have notably changed all over the world ( Juárez *et al.* 2021). Likewise, retail/display meat oxidation and photo-oxidation not only influence the eating quality of the products, but also have harmful effects on the health of consumers by the formation of carcinogenic substances ( Djenane *et al.* 2001, 2003; Domínguez *et al.* 2019; Huang and Ahn 2019). However, as a result of increasing demand for fresh and ready-to-use meats, a need has emerged for adequate preservation techniques to maintain quality and safety ( Djenane and Roncalés 2018). These technologies include chilled storage, vacuum and modified atmosphere packaging (MAP), biopreservation, active packaging and their combination ( Figure 3).

Camel meat is rich in Mb (heme protein) and several authors have reported the pro-oxidant effect of this protein in its oxidized state on lipid oxidation ( Maqsood *et al.* 2015a, b). This makes camel meat more susceptible to oxidation of lipids and therefore to the development of unpleasant odors ( *off-odor*) and can limit its shelf-life during storage ( Maqsood and Benjakul 2010), and reduce its sensory quality, consumer acceptability and marketing of retail displayed fresh camel meats. To overcome this problem, the use of antioxidants is one of the main strategies to prevent the oxidation of proteins and lipids and, consequently, to extend the shelf-life of camel ( Djenane *et al.* 2004, 2016). Nowadays, frozen storage of meat is broadly utilized to extend its shelf-life over one year ( Dang *et al.* 2021) . In addition, most of the previous studies do not take into consideration the impact of freezing storage on nutrient amounts variation in camel meat. Although the preservation of nutrients is a major concern for consumers. Recently, novel strategies are directed towards the use of natural bio preservatives ingredients, which can minimize lipid, pigment oxidation and *off-odor* development with increase of the color stability and consequently the acceptability of camel meat. Therefore, use of natural plant based antioxidants, especially phenolic compounds could be an effective means to prevent lipid and pigment oxidation and it has been well documented to have potential preventive effect on meat alteration during storage ( Djenane *et al.* 2019, 2020). Therefore, plant extracts not only possess antioxidant activity, but also antimicrobial activities against spoilage bacteria and harmful pathogens in camel meat ( Maqsood *et al.* 2015b).

It has been reported that tannic acid and catechin treated camel meat had lowest TBA-RS value, indicating strong inhibitory effect on lipid oxidation in the product during 9 days of storage than caffeic and gallic acids ( Maqsood *et al.* 2015b). Gheisari and Motamedi (2010) found that lipid oxidation in beef muscle was lower than that in camel meat. Camel meat showed the highest mono and polyunsaturated fatty acids content and it might be a reason for its higher lipid oxidation. To remedy this inconvenience, the presence of higher activity of the endogenous antioxidant enzymes such as catalase and GSH-Px could lead to a significant decrease in lipid oxidation during camel meat storage. The PUFAs content is always desirable for human consumption; unfortunately, these FAs are potentially subject to rapid oxidation. It has been shown that a level of lipid oxidation of the order of 1.5 to 2 mg/kg of malondialdehyde in camel meat caused its rejection by a trained panel because of the unpleasant *off-odors* ( Djenane *et al.* 2020). Studies have shown that in some camelid’s, the level of lipid oxidation remains low ( Smith *et al.* 2017a, b; Smith *et al.* 2019). This is due to the probably high levels of vitamin E and other antioxidants reported in the meat of camel which is the result of its supply of aromatic and medicinal herbs from arid regions ( Salva *et al.* 2009; Smith *et al.* 2019). Maqsood *et al.* (2015b) investigated the effect of phenolic compounds on microbial quality of camel meat during refrigerated storage. Results suggested that these compounds were effective in retarding microbial growth in meat by chelating some metal ions that required for microbial growth. In previous study, Djenane *et al.* (2020) found that higher oxygenated MAP (80% O _2_ + 20% CO _2_) combined with effects of refrigerated (1 ± 1 °C) long-term storage and treatment with *Olea europaea* Subsp. *laperrinei* leaf extract alone, or preferably combined with nisin, can be a promising tool and constitute a relevant strategy to control microbial growth and oxidation phenomena of packaged camel meat without undesirable effects on its sensory acceptability during 30 days of storage. These results suggest that the use of these methods could be sustainable for the preservation and promotion of camel meat in arid regions. Equally, Jouki and Khazaei ( Jouki and Khazaei 2012), using anoxic MAP (60% CO _2_ + 40% N _2_) combined with refrigeration (4 °C) storage improved physicochemical attributes and sensory acceptability during three weeks of storage. Recently, Yehia *et al.* (2021) reported that the use of combined natural biopreservative agents (Citrox and chitosan) can be a promising tool and constitute a relevant strategy to control *Campylobacter jejuni* growth and to preserve vacuum packaged camel meat quality during refrigerated storage (4 °C) and at abuse refrigeration (10 °C) for 30 days.

A combination of EOs components like carvacrol, thymol or cinnamaldehyde with the yogurt-based marinade would be complementary to the lethal effects of heating, and consequently improved camel meat safety against *E. coli* O157: H7 and *Salmonella* spp., without significantly changes in the sensory characteristics of the cooked camel meat after storage under normal and at abusive refrigeration ( Osaili *et al.* 2020b). *Mentha spicata* EO was incorporated at different concentrations (0.5 to 1.5% v/w) into raw minced camel meat to extend shelf-life and evaluate its antibacterial activity for 12 days at refrigerated storage. The final microbial population decreased 1 to 4 log cfu/g in treated samples compared to control. Moreover, during storage, peroxide value (PV), total volatile basic nitrogen (TVBN) remained lower in treated minced camel meat than control samples. Color, odor and also appearance of the treated samples were better than control samples ( Shahbazi *et al.* 2018). Similarly, Khezrian and Shahbazi (2018) incorporated of *Ziziphora clinopodioides* EO alone and in combination with *Ficus carica* extract into active packaging films and observed that packed meats with nanocomposite films tended to increases safety (against *Listeria monocytogenes* and *Escherichia coli* O157: H7) and shelf-life of product during refrigerated storage. Sensory attributes were significantly enhanced in treated camel meat samples. Al-Bachir and Zeinou (2009) have evaluated the effect of gamma irradiation (0, 2, 4 and 6 kGy) on chemical, microbial and sensory characteristics of camel meat stored at 1-4 °C. The results indicated that the microbiological shelf-life of irradiated camel meat was significantly extended to more than 6 weeks. Irradiation showed no adverse effects on other meat quality attributes. Referring to previous studies in the literature, few data is available on the use of hurdle technology in preservation of camel meat.

Camel meats remain an essential source of protein for arid region populations. Nonetheless, various traditional meat products have long been known in the region and prepared for family or religious feasts. For example, during “Al Adha feast” Muslim family ought to slaughter a whole camel shared equally between seven (07) families. Surplus meat was then transformed into more stable products. This was achieved by combined treatments such as curing, salting, drying, and fermentation, in an empirical application of the hurdle technology. However, the sanitary and hygienic importance, and the perishable nature of these products prompted public authorities to establish controlled slaughter structures (slaughterhouses). Strict compliance with good hygiene practices in slaughterhouses is therefore essential to prevent bacterial contamination of carcasses, with the aim of maintaining optimal meat quality, thus protecting consumer health ( Wambui *et al.* 2017). It is recommended to use slaughter lines that allow the slaughter process to continue and avoid any contact between carcasses and ground and cross contamination ( Alvseike *et al.* 2019).

## Traceability requirements in the export market

Meat sector traceability is the comprehensive concept of tracking the movement of identifiable products along the marketing chain (source animal: birth or ancestry; through growth and feeding, slaughter, processing and distribution, to the point of sale or consumption) ( Hobbs 2003). Traceability requirement in the export market is one of the serious challenges to the camel meat industry. Today, food safety is worldwide concerns due to a number of food safety scandals have given rise to increased worldwide consumer concern over meat safety and desire for information about the meat products they purchase ( Bánáti 2011). Breeding conditions in intensive systems, where animals live in confined spaces, lead to cross-transmission between animals ( Espinosa *et al.* 2020). Since the crises experienced by the meat sector related to African swine fever, avian flu in poultry, bovine spongiform encephalopathy (BSE), foot and mouth disease in livestock, dioxin, and micro-organisms like *Campylobacter*, *Clostridium*, *Escherichia coli*, *Salmonella*, *Listeria*, Norovirus, all legislation attaches significant importance to:

• Identification and traceability of meat and meat products.
• The establishment of a system of self-control and localization in the supply circuit the origin of the country or production unit ( Schroeder and Tonsor 2012).

At European level, the situation is better. EU actions have certainly led to progress, particularly in the veterinary field, but there is currently evidence to confirm that the health burden of antimicrobial resistance has been reduced. The General Food Law, the EU, has issued a regulation on the labeling of meat and its products to enforce the health monitoring system ( EU, 2011). For camel and camel meat, due to the structure of its supply chain, it will be important for producing countries to develop a tracking system, in order to be able to export camel meat to the European Union and other high value-added markets. With the help of well-targeted processing technologies and information on the quality characteristics of meat from various muscles, it enables the camel meat sector to develop an efficient marketing system based on more attractive cuts with better quality characteristics ( Kadim *et al.* 2013).

The herd’s fertility ( Gherissi *et al.* 2020) and the lack of specialized state health services for camelids, coupled with the difficulties for non-specialist veterinarians to reach remote areas, and no cooperative breeders lead to a high incidence of fetal wastage due to slaughtering of pregnant females. The lack of specific and reliable identification and traceability system; it is also difficult to record herd’s because of pastoralists’ illiteracy. Although these statistics are far from reflecting the actual consumption given the large number of illegally slaughtered camels (sterile females and sick camels). Large numbers of camels are also slaughtered in emergency situations due to frequent road accidents when the herds of camels are left free.

### Halal certification

The Muslim world is the most important market for camel meat. Therefore, the lack of Halal (permissible to use in according to the Islamic law: Shari’a) certification is a serious weakness. It should also be noted that the world market for camel meat is closely linked to the Arab-Muslim world and therefore the meat available in the markets must be Halal ( Bux *et al.* 2022). This effectively limits the flow of products from countries that cannot issue Halal certification. Under traditional conditions, for example in Sudan, the animal is sometimes stunned before bleeding, and then standard procedures are implemented to allow Halal certification in Muslim countries. Adulteration of meat products with cheaper other meats species of different origin during their preparation is a common practice around the world ( Scarano and Rao 2014). Adulteration and mislabeling of meat raises many economic, health, and religious concerns. The 2013 horse meat fraud is an example of food fraud perpetrated across Europe by passing on horse meat as beef. In this context, the detection of animal species in meat products essentially meets religious and competitive requirements ( Zhao *et al.* 2020) and allows the enforcement of labeling legislation and prevention of unfair competition in meat sector.

## Recommendations

A valid alternative to beef and other red meats could be the camel meat. North African countries are the largest African producer of camel meat and derivatives. The high nutritional properties of camel meat make it suitable for inclusion in the Mediterranean diet in order to adapt it to the needs and conditions of the population. Consumers tend dislike camel meat because they associate meat with the camel itself, and this is often one of the reasons for this disapproval. Under these circumstances, it would be ideal for manufacturers to avoid using promotional labels that show the image of the camel itself. This problem could be addressed through commercial advertising and education. It empowers consumers, helping them acquire the skills and attitudes they need to be able to gear the choices they make to their economic and health interests. Australians have succeeded in promoting kangoro meat and lessons can also be drawn from the same approaches. Consumers describe camel meat as tough even though they agree that it is no different from beef in terms of flavor. The meat consumption behavior by consumers will contribute to the development of livestock camel sector. However, almost all research has proven that the older a camel, the tougher the meat. Research has established, however, that it is generally accepted that younger animals produce more tender meat than older animals. Therefore, age is an important factor in determining the quality of camel meat. There are potential opportunities for camel meat industry through brand development. Future camel research should focus on exploiting its meat potential in the same way as dairy through interdisciplinary research on efficient production systems, improved meat technology and marketing. Finally, the consumer dietary for lower or no meat consumption (e.g. vegetarian or vegan diets) or for cultured meat are assumed to expand slowly and to be adopted by a small part of population concentrated mainly in rich countries, and therefore hardly affect meat consumption over the next decade ( Chriki and Hocquette 2020). Main constraints of camel production such as presence of camel disease, poor overall knowledge of producer, lack or insufficient market infrastructure, lack of market information and lack or insufficient overall support. Addressing these constraints through targeted interventions, such as disease control programs, capacity-building initiatives, improved market linkages, and policy support, can help to enhance the sustainability and profitability of camel production systems.

## Conclusions

Variation in nutritional composition of camel meat such as effect of age at which the camel is slaughtered plays a significant role in determining the meat’s composition and in turn, affects consumer acceptance. Also, the nutritional composition of camel meat shows considerable diversity based on the specific muscle being studied. Different muscle portions have distinct compositions, influencing the overall nutritional profile of the meat. In addition, variations in the chemical composition of camel meat have been observed due to differences in breeds. Different breeds of camels may exhibit varying nutritional properties in their meat.

Camel meat has been processed into limited camel products to add value. It is also interesting to draw the attention of researchers from arid and semi-arid countries as well as stakeholders including decision-makers in these countries to the urgency of assessing or identifying the health risks associated with consumption of camel products and thus takes the necessary measures to reduce these risks. In the light of this review, the need to implement a control strategy is obvious. Veterinary laboratories should be provided with accurate diagnostic tools in order to detect infected animals that represent potential reservoirs of infection at an early stage. Especially during the seasons with abundant vectors.

The camel meat, which is produced naturally and biologically, would occupy important place on the global market. Though the scientific research achievements are modest, they open new up horizons for the modernization of the sector in order to improve the productive performances of camels in arid regions. Suitable chilling and innovative packaging technologies, are required to augment hygienic meat production could lead to the development of expanded markets for camel meat not only in the Middle East and North Africa but also globally. Slaughterhouse solid waste management and effluent treatment are some of the parts that need better technological involvement.

## Data Availability

Not applicable.
