# Supplementary material for: The one-humped camel: The animal of future, potential alternative red meat, technological suitability and future perspectives
Source: F1000Res. 2024 Jul 5;11:1085. Originally published 2022 Sep 22. [Version 3] doi: 10.12688/f1000research.125246.3 (PMC11128057; doi:10.12688/f1000research.125246.3)
Supplement: Peer review history

# Reviewer report: Reviewer response for version 2

**DOI:** 10.5256/f1000research.141626.r236504

**Published:** 2024-01-30

**Authors:** Waleed Al-Marzooqi

**Affiliations:** 1 Sultan Qaboos University, Muscat, Muscat Governorate, Oman

The authors responded adequately to the raised points. The manuscript has been significantly improved.

Is the review written in accessible language?

Yes

Are all factual statements correct and adequately supported by citations?

Yes

Are the conclusions drawn appropriate in the context of the current research literature?

Partly

Is the topic of the review discussed comprehensively in the context of the current literature?

Partly

Reviewer Expertise:

Research Interests:·       Animal responses to nutrients·       Assessment of nutritional value of feedstuffs.·     Quantification the impact of anti-nutritional factors together with processing conditions.·     Meat production and science

I confirm that I have read this submission and believe that I have an appropriate level of expertise to confirm that it is of an acceptable scientific standard.

# Reviewer report: Reviewer response for version 1

**DOI:** 10.5256/f1000research.137529.r153807

**Published:** 2024-05-25

**Authors:** Mounir Hassani Zerrouk, Ouiam El Galiou

**Affiliations:** 1 Research team in Biotechnology and biomolecular engineering, FSTT, Abdelmalek Essaadi University, Tetouan, Morocco

**Notes: **This particular article offers an interesting insight on the possible applications and consideration of the camel meat as a potential source for meat production. The article discusses in great detail the important compounds contained in camel meat which could represent a significant source for healthy active principals, therefore proving the beneficial aspects of the camel meat consumption, as well as highlighting several properties of the mentioned product in order to further support the claims and validity of the use of this product as a healthier alternative. The authors have equally ensured to support the data mentioned in the article with relative and recent research as references to the statements made. Another interesting aspect of the offered study is the technological aspects section which represents the potential strategies for the exploitation of camel meat as an environment friendly substitute to red meats as well as potential methods for its storage/shelf life extension.

 The following list contains minor corrections which need to be taken into consideration:

**Abstract**

1. Replace: "the arid regions" with "arid regions".
2. Replace: "can be benefit" with "can benefit".
3. Replace: "Research on the camel meat" with "Research on camel meat".
4. Replace: "the limited information available on this area" with "the limited information available **in** this area".
5. Replace: "developing new preservation techniques as well the development of various products" with "as well **as** the development of various products".
6. The word 'future' should be represented without a hyphen.
7. Replace: "The camel meat as an alternative source to red meats" with " **Camel** meat as an alternative source to red meats".

**Introduction:**

1. Replace: "This come from countries where technical and regulatory developments have made it possible to promotion the camel meat industry and improve product quality" with "This **comes** from countries where technical and regulatory developments have made it possible to **promote** the camel meat industry and improve product quality".
2. Replace: "This review discusses the nutritional and qualitative properties of camel meat, explains its versatility and positive effects on human health….., in the view of the most" with "This review discusses the nutritional and qualitative properties of camel meat, **and **explains its versatility and positive effects on human health…….., **in view** of the most..."
3. Replace: "Consumers tend dislike camel meat because they associate meat with the camel itself", with "Consumers tend **to **dislike camel meat because they associate meat with the camel itself".
4. Replace: "The Australian meat industry has been successfully promoted" with "The Australian meat industry has successfully promoted".
5. "Bioactive compounds in **camel meat Camel meat** is characterized by a considerable amount": the word camel meat seems to be repeated twice at the beginning of the sentence.
6. Replace: "by British Department of Health (BDH 1994). Ayyash et al. (2019) claim that camel meat was traditionally used" with "by **the** British Department of Health (BDH 1994). Ayyash et al. (2019) claim that camel meat was traditionally used".
7. Replace: "particularly Middle East, Asia, and Africa, but the use of" with "particularly **the** Middle East, Asia, and Africa, but the use of".
8. Replace: "However, during the 20th century, when intercultural migration of people became very important, knowledge about camels and their products began to reach countries beyond Middle East, Asia and Africa" with "However, during the 20th century, when **the ** intercultural migration of people became very important, knowledge about camels and their products began to reach countries beyond **the** Middle East, Asia and Africa".
9. Replace: "a higher proportion of acyl carnitine in plasma and skeletal muscle of the camel than other animal species suggests an adaptive mechanism that" with "a higher proportion of acyl carnitine in plasma and skeletal muscle of the camel than **in** other animal species suggests an adaptive mechanism".
10. Replace: "that may cause loss of juice displaying a direct impact on the nutrient content of cooked meats" with " **which** may cause loss of juice displaying a direct impact on the nutrient content of cooked meats".
11. Replace: "Camels also have a very efficient feed conversion rate. Nowdays consumers" with "Camels also have a very efficient feed conversion rate. **Nowadays** consumers".

In general, this article offers a concise and complete study around the use and application of camel meat as a novel source of meat products and their derivatives while respecting the appropriate steps to introducing a review article and offering an open invitation towards potential studies with clear and relevant perspectives and recommendations.

Is the review written in accessible language?

Yes

Are all factual statements correct and adequately supported by citations?

Yes

Are the conclusions drawn appropriate in the context of the current research literature?

Yes

Is the topic of the review discussed comprehensively in the context of the current literature?

Yes

Reviewer Expertise:

Food science

We confirm that we have read this submission and believe that we have an appropriate level of expertise to confirm that it is of an acceptable scientific standard.

# Response

**Published:** 2024-06-24

**Authors:** djamel djenane

**Affiliations:** unversity of tizi-ouzou, Algeria

**Abstract**

1. Replace: "the arid regions" with "arid regions". It's done
2. Replace: "can be benefit" with "can benefit". It's done
3. Replace: "Research on the camel meat" with "Research on camel meat". It's done
4. Replace: "the limited information available on this area" with "the limited information available **in** this area". It's done
5. Replace: "developing new preservation techniques as well the development of various products" with "as well **as** the development of various products". It's done
6. The word 'future' should be represented without a hyphen. OK, the hyphen is removed.
7. Replace: "The camel meat as an alternative source to red meats" with " **Camel** meat as an alternative source to red meats". It's done

**Introduction:**

1. Replace: "This come from countries where technical and regulatory developments have made it possible to promotion the camel meat industry and improve product quality" with "This **comes** from countries where technical and regulatory developments have made it possible to **promote** the camel meat industry and improve product quality". It's done
2. Replace: "This review discusses the nutritional and qualitative properties of camel meat, explains its versatility and positive effects on human health….., in the view of the most" with "This review discusses the nutritional and qualitative properties of camel meat, **and **explains its versatility and positive effects on human health…….., **in view** of the most...". It's done
3. Replace: "Consumers tend dislike camel meat because they associate meat with the camel itself", with "Consumers tend **to **dislike camel meat because they associate meat with the camel itself". It's done
4. Replace: "The Australian meat industry has been successfully promoted" with "The Australian meat industry has successfully promoted". It's done
5. "Bioactive compounds in **camel meat Camel meat** is characterized by a considerable amount": the word camel meat seems to be repeated twice at the beginning of the sentence. it's corrected
6. Replace: "by British Department of Health (BDH 1994). Ayyash et al. (2019) claim that camel meat was traditionally used" with "by **the** British Department of Health (BDH 1994). Ayyash et al. (2019) claim that camel meat was traditionally used". It's done
7. Replace: "particularly Middle East, Asia, and Africa, but the use of" with "particularly **the** Middle East, Asia, and Africa, but the use of". It's done
8. Replace: "However, during the 20th century, when intercultural migration of people became very important, knowledge about camels and their products began to reach countries beyond Middle East, Asia and Africa" with "However, during the 20th century, when **the ** intercultural migration of people became very important, knowledge about camels and their products began to reach countries beyond **the** Middle East, Asia and Africa". It's done
9. Replace: "a higher proportion of acyl carnitine in plasma and skeletal muscle of the camel than other animal species suggests an adaptive mechanism that" with "a higher proportion of acyl carnitine in plasma and skeletal muscle of the camel than **in** other animal species suggests an adaptive mechanism". It's done
10. Replace: "that may cause loss of juice displaying a direct impact on the nutrient content of cooked meats" with " **which** may cause loss of juice displaying a direct impact on the nutrient content of cooked meats". It's done
11. Replace: "Camels also have a very efficient feed conversion rate. Nowdays consumers" with "Camels also have a very efficient feed conversion rate. **Nowadays** consumers". It's done

# Reviewer report: Reviewer response for version 1

**DOI:** 10.5256/f1000research.137529.r187921

**Published:** 2023-07-26

**Authors:** Waleed Al-Marzooqi

**Affiliations:** 1 Sultan Qaboos University, Muscat, Muscat Governorate, Oman

The manuscript requires substantial reworking due to the presence of repetitive sentences, particularly between the recommendation and conclusion sections. To improve the clarity and quality, the recommendation should be condensed into a concise paragraph that highlights the primary assumptions and limitations. On a positive note, the language and style meet the required standards.

 Moreover, it is crucial to ensure a clear and concise statement of the objective in the abstract to provide readers with a comprehensive understanding of the study's focus.

 Furthermore, the authors should dedicate more attention to elaborating and discussing the points mentioned below. These crucial pieces of information are currently absent from the manuscript and their inclusion through thorough examination is necessary to enhance the overall quality of the article.

  

1. Variation in nutritional composition of camel meat such as effect of Age at which the camel is slaughtered plays a significant role in determining the meat's composition and in turn, affects consumer acceptance. Also, the nutritional composition of camel meat shows considerable diversity based on the specific muscle being studied. Different muscle portions have distinct compositions, influencing the overall nutritional profile of the meat. In addition, variations in the chemical composition of camel meat have been observed due to differences in breeds. Different breeds of camels may exhibit varying nutritional properties in their meat.
  Understanding these variations is essential for researchers, meat processors, and consumers to make informed decisions regarding camel meat consumption and utilization. By considering these factors, stakeholders can optimize the production and marketing of camel meat products, meeting the preferences and demands of consumers while ensuring the meat's overall quality and nutritional value.
2. Main constraints of camel production such as presence of camel disease, poor overall knowledge of producer, lack or insufficient market infrastructure, lack of market information and lack or insufficient overall support. Addressing these constraints through targeted interventions, such as disease control programs, capacity-building initiatives, improved market linkages, and policy support, can help to enhance the sustainability and profitability of camel production systems.
3. Camel rearing and production and slaughtering and processing. Both camel rearing and production, as well as slaughtering and processing, play pivotal roles in determining the overall quality, safety, and value of camel meat in the market.

Is the review written in accessible language?

Yes

Are all factual statements correct and adequately supported by citations?

Yes

Are the conclusions drawn appropriate in the context of the current research literature?

Partly

Is the topic of the review discussed comprehensively in the context of the current literature?

Partly

Reviewer Expertise:

Research Interests:·       Animal responses to nutrients·       Assessment of nutritional value of feedstuffs.·     Quantification the impact of anti-nutritional factors together with processing conditions.·     Meat production and science

I confirm that I have read this submission and believe that I have an appropriate level of expertise to state that I do not consider it to be of an acceptable scientific standard, for reasons outlined above.

# Response

**Published:** 2023-11-21

**Authors:** djamel djenane

**Affiliations:** unversity of tizi-ouzou, Algeria

**Responses to Reviewer Comments**

 The authors appreciated the careful review and greatly acknowledge the comments and manuscript overall evaluation provided. The authors made the recommended changes and responded in detail to the questions raised by the reviewer.

**Reviewer#1:** The manuscript requires substantial reworking due to the presence of repetitive sentences, particularly between the recommendation and conclusion sections. To improve the clarity and quality, the recommendation should be condensed into a concise paragraph that highlights the primary assumptions and limitations. On a positive note, the language and style meet the required standards.

***Response:*** *Many thanks for your consideration and valuable comments. The recommendation has been modified according to the reviewer suggestions.*

**Reviewer#1:** Moreover, it is crucial to ensure a clear and concise statement of the objective in the abstract to provide readers with a comprehensive understanding of the study's focus.

***Response:*** *We have carefully considered comments and revised the manuscript. We add more information about main objectives of the paper.*

 Reviewer#1: Furthermore, the authors should dedicate more attention to elaborating and discussing the points mentioned below. These crucial pieces of information are currently absent from the manuscript and their inclusion through thorough examination is necessary to enhance the overall quality of the article.

***Response:*** *We have carefully considered your comments and revised the manuscript. We hope that you will be pleased with this revision. New references were added to the text regarding the points added.*

 Reviewer#1: Variation in nutritional composition of camel meat such as effect of Age at which the camel is slaughtered plays a significant role in determining the meat's composition and in turn, affects consumer acceptance. Also, the nutritional composition of camel meat shows considerable diversity based on the specific muscle being studied. Different muscle portions have distinct compositions, influencing the overall nutritional profile of the meat. In addition, variations in the chemical composition of camel meat have been observed due to differences in breeds. Different breeds of camels may exhibit varying nutritional properties in their meat.

***Response:*** *Thank you. Additional data on the chemical composition of camel meat and its variation factors have been added to the manuscript.*

 Reviewer#1: Main constraints of camel production such as presence of camel disease, poor overall knowledge of producer, lack or insufficient market infrastructure, lack of market information and lack or insufficient overall support. Addressing these constraints through targeted interventions, such as disease control programs, capacity-building initiatives, improved market linkages, and policy support, can help to enhance the sustainability and profitability of camel production systems.

***Response:*** *We appreciate your good comments. We have carefully revised the manuscript by introducing the main constraints of camel production such as camel disease, poor overall knowledge of the producer, lack or insufficiency of market infrastructure, lack of information on the market and the lack or insufficiency of overall support. Effectively addressing these constraints can help improve the sustainability and cost-effectiveness of camel production systems.*

**Reviewer#1:** Camel rearing and production and slaughtering and processing. Camel rearing and production, as well as slaughtering and processing, play pivotal roles in determining the overall quality, safety, and value of camel meat in the market

***Response:*** *The discussion regarding these issues was improved.*

*Thank you very much*

*Pr Dr Djenane Djamel*
